# Validity of the MPTP-Treated Mouse as a Model for Parkinson’s Disease

**DOI:** 10.1007/s12035-015-9103-8

**Published:** 2015-02-13

**Authors:** Cornelius J. H. M. Klemann, Gerard J. M. Martens, Geert Poelmans, Jasper E. Visser

**Affiliations:** 1 Department of Molecular Animal Physiology, Donders Institute for Brain, Cognition and Behaviour, Radboud University, Nijmegen, The Netherlands; 2 Department of Cognitive Neuroscience, Donders Institute for Brain, Cognition and Behaviour, Radboud University Medical Center, Nijmegen, The Netherlands; 3 Department of Human Genetics, Radboud University Medical Center, Nijmegen, The Netherlands; 4 Department of Neurology, Donders Institute for Brain, Cognition and Behaviour, Radboud University Medical Center, P.O. Box 9101, 6500 HB Nijmegen, The Netherlands; 5 Department of Neurology, Amphia Hospital, Breda, The Netherlands

**Keywords:** Parkinson’s disease, MPTP mouse model, Genome-wide mRNA expression, Molecular landscape

## Abstract

Parkinson’s disease (PD) is characterized by dopaminergic (DA) neuron death in the substantia nigra (SN) and subsequent striatal adaptations. Mice treated with the neurotoxin 1-methyl-4-phenyl-1,2,3,6-tetrahydropyrimidine (MPTP) are widely used as a model for PD. To assess the validity of the MPTP mouse model for PD pathogenesis, we here identify the biological processes that are dysregulated in both human PD and MPTP-treated mice. Gene enrichment analysis of published differentially expressed messenger RNAs (mRNAs) in the SN of PD patients and MPTP-treated mice revealed an enrichment of gene categories related to motor dysfunction and neurodegeneration. In the PD striatum, a similar enrichment was found, whereas in the striatum of MPTP mice, acute processes linked to epilepsy were selectively enriched shortly following MPTP treatment. More importantly, we integrated the proteins encoded by the differentially expressed mRNAs into molecular landscapes showing PD pathogenesis-implicated processes only in the SN, including vesicular trafficking, exocytosis, mitochondrial apoptosis, and DA neuron-specific transcription, but not in the striatum. We conclude that the current use of the MPTP mouse as a model for studying the molecular processes in PD pathogenesis is more valid for SN than striatal mechanisms in PD. This novel insight has important practical implications for future studies using this model to investigate PD pathogenesis and evaluate the efficacy of new treatments.

**Electronic supplementary material:**

The online version of this article (doi:10.1007/s12035-015-9103-8) contains supplementary material, which is available to authorized users.

## Introduction

1-Methyl-4-phenyl-1,2,3,6-tetrahydropyridine (MPTP), a toxic impurity that may occur during the synthesis of the opioid drug desmethylprodine, causes an irreversible parkinsonian syndrome in humans almost indistinguishable from Parkinson’s disease (PD) [1]. Therefore, MPTP toxicity in monkeys, rats, and mice has been studied to elucidate the pathogenic mechanisms implicated in PD. MPTP-treated mice are advantageous to explore the molecular background of MPTP toxicity, because lines of genetically engineered animals allow high levels of control of the experimental conditions. Mice treated with MPTP share specific biological features with PD, including loss of dopaminergic (DA) neurons in the substantia nigra (SN) and dopamine depletion in the striatum [2]. However, their pathogenetic backgrounds are different, being a toxic nature in a mouse model and a neurodegenerative process in human PD. Moreover, not all PD phenomenology is reproduced in MPTP-treated mice [3]. Therefore, the construct validity of the MPTP mouse as a model to study and elucidate the pathogenesis of PD remains unclear.

In order to identify the biological processes that are dysregulated in MPTP toxicity and their relationship to PD pathogenesis, differentially expressed messenger RNAs (mRNAs) from postmortem SN and striatum of PD patients, as well as differentially expressed mRNAs in the SN and striatum of MPTP-treated mice, were analyzed. Furthermore, based on proteins encoded by the mRNAs that were differentially expressed in both PD patients and MPTP mice, molecular landscapes of interacting proteins were built for both the SN and striatum. These landscapes represent molecular mechanisms that are shared between PD and MPTP toxicity. Together, these analyses will help to understand and value experimental findings in the MPTP mouse in the light of human PD pathogenesis.

## Materials and Methods

### Genome-Wide mRNA Expression Data

Available genome-wide mRNA expression data from multiple previously published studies were used to generate a list of differentially expressed transcripts in the postmortem SN and striatum of PD patients and MPTP-treated mice, studied at various time points following MPTP treatment. If raw expression data was available at the Gene Expression Omnibus (GEO) site, this data was reanalyzed in GeneSifter (www.genesifter.com) using robust microarray analysis (RMA). The Benjamini-Hochberg method was then used to correct for multiple comparisons, and only mRNAs with a fold change (FC) of ≥1.2 or ≤−1.2 and a corrected *p* value <0.05 were considered to be differentially expressed and used for the subsequent gene enrichment analysis, as described below. If no raw data was available, our inclusion criteria were the following: (1) correction for multiple testing was performed, with a corrected *p* value <0.05 and the correction method was explicitly mentioned; and (2) an mRNA expression FC of ≥1.2 or ≤−1.2. Only protein-coding mRNAs were included in our analyses.

### Enrichment Analysis

The Ingenuity pathway analysis software package (www.ingenuity.com) was used to identify enriched gene categories in the lists of differentially expressed mRNAs in the SN and striatum of both human PD patients and MPTP-treated mice and in the lists of overlapping mRNAs that were differentially expressed in the SN or striatum of both PD patients and MPTP mice. Ingenuity assigns genes and their corresponding mRNAs/proteins to (sub)categories of functional classes, e.g., “diseases and disorders” and “molecular and cellular functions.” For these analyses, only functional categories and pathways with significant enrichment (i.e., Benjamini-Hochberg corrected *p* < 0.05) and containing two or more genes were taken into account.

### Molecular Landscape Building

Subsequently, the mRNAs that were differentially expressed in the SN and striatum of both PD patients and MPTP-treated mice were analyzed in more depth. Guided by the results of the Ingenuity enrichment analyses, the literature was searched for the (putative) function of all the proteins encoded by the mRNAs overlapping between human PD and the MPTP mouse, as well as their functional interactions, using the UniProt Knowledgebase (http://www.uniprot.org/uniprot) [4] and PubMed (http://www.ncbi.nlm.nih.gov/sites/entrez). Based on these findings and applying an approach similar to the one we used previously to build landscapes based on genome-wide association data [5, 6], we then built two molecular landscapes comprising interacting proteins encoded by the overlapping mRNAs in the SN and striatum, respectively. To complement these protein interaction cascades, we also added a number of proteins that were not encoded by the overlapping differentially expressed mRNAs but that have been implicated in PD etiology through other lines of (genetic) evidence. In this respect, proteins encoded by familial PD candidate genes were included if they have at least one functional interaction with one or more other landscape proteins. Additional proteins were included when having at least two interactions with other landscape proteins. Serif Drawplus 4.0 (www.serif.com) was used to draw the landscape figures.

## Results

In this study, we analyzed with gene enrichment approaches and systematic literature searches published datasets of differentially expressed transcripts in SN and striatum of PD patients and MPTP-treated mice that met our criteria for inclusion (see Table 1 for dataset details).

**Table 1:** Datasets of differentially expressed transcripts in SN and striatum of PD patients and MPTP-treated mice that met the criteria for inclusion

Species	Gender	Reference (GEO accession)	Cases/controls	Substrate	Microarray platform	FC cutoff (up/down)	Statistics	Number of significant genes
Human	M/F	Zhang, 2005 [30]	11/18	SN	Affymetrix Human Genome U133A Array	1.2	B&H *p* < 0.05	26
Human	M/F	Moran, 2006 [31] (GSE8397)	15/7	Medial SN	Affymetrix Human Genome U133A Array	1.2	B&H *p* < 0.05	600
			15/7	Medial SN	Affymetrix Human Genome U133B Array	1.2	B&H *p* < 0.05	310
			9/6	Lateral SN	Affymetrix Human Genome U133A Array	1.2	B&H *p* < 0.05	170
			9/6	Lateral SN	Affymetrix Human Genome U133B Array	1.2	B&H *p* < 0.05	95
Human	M/F	Cantuti-Castelvetri, 2007 [32]	8/8	SN (LCM DA neurons)	Affymetrix Human X3P	2.0	SAM q < 0.05	31
Human	M/F	Lesnick, 2007 [33] (GSE7621)	16/9	SN	Affymetrix Human Genome U133 Plus 2.0 Array	1.2	B&H *p* < 0.05	42
Human	M/F	Bossers, 2009 [34]	4/4	SN	Agilent 22 k 60mer oligonucleotide array	1.4	Bonferoni *p* < 0.05	259
Human	M/F	Zheng, Liao, 2010 [35] (GSE20141)	10/8	SN (LCM DA neurons)	Affymetrix Human Genome U133 Plus 2.0 Array	1.2	B&H *p* < 0.05	0
		(GSE20163)	8/9	SN	Affymetrix Human Genome U133A Array	1.2	B&H *p* < 0.05	0
		(GSE20164)	6/5	SN	Affymetrix Human Genome U133A Array	1.2	B&H *p* < 0.05	0
Human	M/F	Elstner, 2011 [36]	8/9	SN (LCM DA neurons)	Illumina WG6v1 expression chip	1.2	B&H *p* < 0.05	1037
Human	M/F	Diao, 2012 [37] (GSE20333)	6/6	SN	Affymetrix Human HG-Focus Target Array	1.2	B&H *p* < 0.05	0
Human	M/F	Zhang, 2005 [30]	15/20	Putamen	Affymetrix Human Genome U133A Array	1.2	B&H *p* < 0.05	1
Human	M/F	Vogt, 2006 [38]	8/8	Putamen	Affymetrix Human Genome U133A Array	2.0	B-Y *p* < 0.05	78
Human	M/F	Botta-Orfila, 2012 [39]	5/5	Putamen	Affymetrix 1.0 Exon	2.0	B&H *p* < 0.05	186
Mouse	M	Miller, 2004 [7] (GSE4788)	24/12	SN	Affymetrix Murine Genome U74A Array	1.2	B&H *p* < 0.05	608
Mouse	F	Pattarini, 2008 [40]	3/6	Striatum	Affymetrix Mouse Genome 430 2.0 Arrays	1.5	B&H *p* < 0.05	430

*B&H* Benjami and Hochberg, *B*-*Y* Benjami-Yekutieli, *DA* dopamine, *F* female, *FC* fold change, *GEO* gene expression omnibus, *M* male, *LCM* laser capture microdissecton, *SAM* significance analysis of microarrays, *SN* substantia nigra

### Enrichment Analysis of SN mRNA Expression Data

#### Human PD

Ingenuity enrichment analysis of the mRNAs that, compared to healthy controls, were differentially expressed in the SN of human PD patients revealed the subcategories that were most significantly enriched within the two main functional classes, “diseases and disorders” and “molecular and cellular functions” (Table 2). When analyzing all differentially expressed SN mRNAs, the most significantly enriched diseases and disorders were predominantly in the movement disorders domain. Similar annotations were found for the top 5 enriched categories within the downregulated mRNAs, while the enriched annotations within the upregulated mRNAs were not specifically related to (any) neurological function (data not shown). At a more functional level (i.e., the “molecular and cellular functions” category), the enriched annotations were all related to neuronal and/or synaptic function.

**Table 2:** Gene enrichment analysis of the substantia nigra

	MPTP mouse						Human PD			Overlap			
Rank	Shorter interval	*p* value	#	Longer interval	*p* value	#		*p* value	#		*p* value	#	Overlapping genes
Category: diseases and disorders													
1	Movementdisorders	1.54E-16	89	Movement disorders	3.41E-10	58	Disorder of basal ganglia	2.35E-22	203	Dyskinesia	1.91E-07	24	ACHE, ATP5C1, BCL2, CDH2, CDK5, DDX1, FAM3C, GABRG2, GRIN1, MAP2K4, NDRG1, PFKM, RAB11A, RAB6A, RGS4, RTN2, SLC6A3, SNAP25, SOX2, ST8SIA3, TH, VAMP2, VSNL1, YWHAZ
2	Disorder of basal ganglia	1.30E-12	66	Neuromuscular disease	2.48E-07	45	Movement disorders	4.33E-20	250	Disorder of basal ganglia	2.86E-07	26	ACHE, ATP5C1, BCL2, CDH2, CDK5, DDX1, EIF4G1, FAM3C, GABRG2, GRIN1, MAP2K4, NDRG1, NR4A2, PFKM, RAB11A, RAB6A, RGS4, RTN2, SLC6A3, SNAP25, SOX2, ST8SIA3, TH, VAMP2, VSNL1, YWHAZ
3	Dyskinesia	3.02E-12	57	Neurological signs	1.26E-06	36	Neuromuscular disease	6.42E-19	211	Neuromuscular disease	5.35E-07	27	ACHE, ATP5C1, BCL2, CDH2, CDK5, DDX1, EIF4G1, FAM3C, GABRG2, GRIN1, MAP2K4, NDRG1, NR4A2, PFKM, RAB11A, RAB6A, RGS4, RPL5, RTN2, SLC6A3, SNAP25, SOX2, ST8SIA3, TH, VAMP2, VSNL1, YWHAZ
4	Neurological signs	3.02E-12	58	Disorder of basal ganglia	1.63E-06	40	Chorea	1.24E-16	154	Huntington’s disease	5.78E-07	22	ATP5C1, BCL2, CDH2, CDK5, DDX1, FAM3C, GABRG2, GRIN1, MAP2K4, NDRG1, PFKM, RAB11A, RAB6A, RGS4, RTN2, SLC6A3, SNAP25, SOX2, ST8SIA3, VAMP2, VSNL1, YWHAZ
5	Huntington’s disease	1.11E-11	54	Dyskinesia	1.74E-06	35	Neurological signs	1.41E-16	163	Movement disorders	1.70E-06	28	ACHE, ATP5C1, ATXN10, BCL2, CDH2, CDK5, DDX1, EIF4G1, FAM3C, GABRG2, GRIN1, MAP2K4, NAPB, NDRG1, NR4A2, PFKM, RAB11A, RAB6A, RGS4, RTN2, SLC6A3, SNAP25, SOX2, ST8SIA3, TH, UGT8, VAMP2, VSNL1, YWHAZ
Category: molecular and cellular functions													
1	Cell death	1.11E-12	170	Proliferation of cells	2.28E-11	128	Transport of vesicles	9.27E-11	40	Neuronal cell death	5.01E-05	20	ACHE, AKT1S1, BCL2, CDK5, FYN, GRIN1, KIFAP3, L1CAM, MAGED1, MAP2K4, MAPK8, NFKBIA, NR4A2, RET, SLC6A3,SNAP25, SOX11, SRPK2, STXBP1, YWHAZ
2	Microtubule dynamics	1.30E-12	77	Cell death	3.41E-10	118	Formation of plasma membrane projections	6.45E-08	112	Microtubule dynamics	9.35E-05	25	ACTG1, ATXN10, BCL2, CDH2, CDK5, CHP1, CRMP1, FYN, GRIN1, IFT20, KLC1, L1CAM, LPAR1, MAP2K4, MAP4, MAPK8, MARK2, NDRG1, NFIB, NFKBIA, RAB11A, RANBP9, RET, TNK2, UGT8
3	Organization of cytoskeleton	1.30E-12	85	Proliferation of tumor cell lines	8.64E-09	67	Microtubule dynamics	8.73E-08	195	Synthesis of neurotransmitter	9.67E-05 s	6	BCL2, NR4A2, SLC6A3, SNAP25, TH, YWHAZ
4	Organization of cytoplasm	4.30E-12	88	Apoptosis	1.13E-08	97	Organization of cytoplasm	1.09E-07	237	Exocytosis by cells	1.05E-04	8	CDK5, GNAI2, NAPB, NSF, RAB11A, SNAP25, STXBP1, VAMP2
5	Proliferation of cells	1.96E-11	173	Degeneration of cells	2.24E-08	24	Formation of cellular protrusions	1.47E-07	149	Production of catecholamine	1.54E-04	5	BCL2, NR4A2, SLC6A3, TH, YWHAZ

Ingenuity annotations of genes dysregulated by MPTP in the mouse substantia nigra, after short and longer intervals between treatment and analyses (608 genes for intervals combined), annotations of all dysregulated genes in the substantia nigra of PD patients (2027 genes), and those that are dysregulated in both the MPTP mouse model and human PD (i.e., 116 “overlapping” genes). Data are extracted from references in Table 1. The top 5 Ingenuity annotations of the categories “diseases and disorders” and “molecular and cellular functions” are displayed, as well as their respective *p* value and number of genes involved (#). All *p* values are corrected for multiple testing by the Benjamini-Hochberg false discovery rate. Categories with only one (target) gene were discarded. See text for further details

#### MPTP Mouse

Similar Ingenuity analyses revealed the most enriched functional categories within the mRNAs that were differentially expressed in the SN of MPTP-treated mice compared to untreated animals (Table 2). Analyzing mRNA expression profiling data at different intervals following MPTP treatment assessed temporal aspects of MPTP-induced neurotoxicity. More specifically, mice were injected four times within an 8-day period and subsequently sacrificed for analysis 1 and 7 days after the last treatment [7]. The enriched diseases and disorders were, at both intervals, predominantly in movement disorders-related domains. The enriched molecular and cellular functions categories were also similar over time, relating mainly to cell death, proliferation, and development (both intervals), as well as to structural organization of the cell (short interval only).

#### Overlap Between Human PD and MPTP Mouse

The most significantly enriched functional categories within the mRNAs that were differentially expressed in the SN of *both* PD patients and MPTP-treated mice are also shown in Table 2. Similar to the human and mouse results mentioned above, the enriched diseases and disorders encompass movement disorders-related domains, while the more functional categories related to neuronal cell death, microtubule dynamics, and cellular functions, including neurotransmitter synthesis and exocytosis, as well as (neuronal) cell growth and death.

### Molecular Landscape of Shared Processes in the SN

**Fig. 1:**
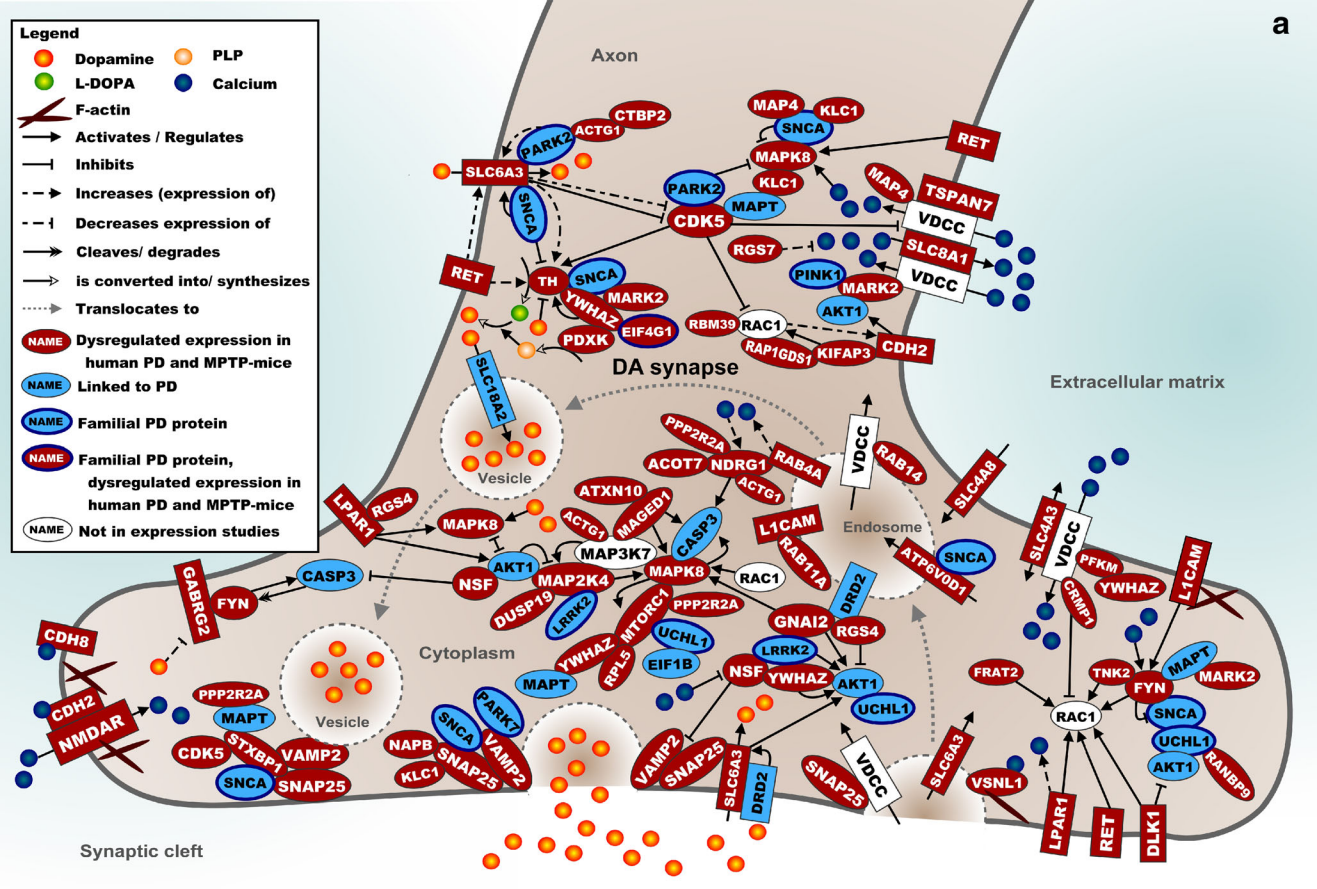

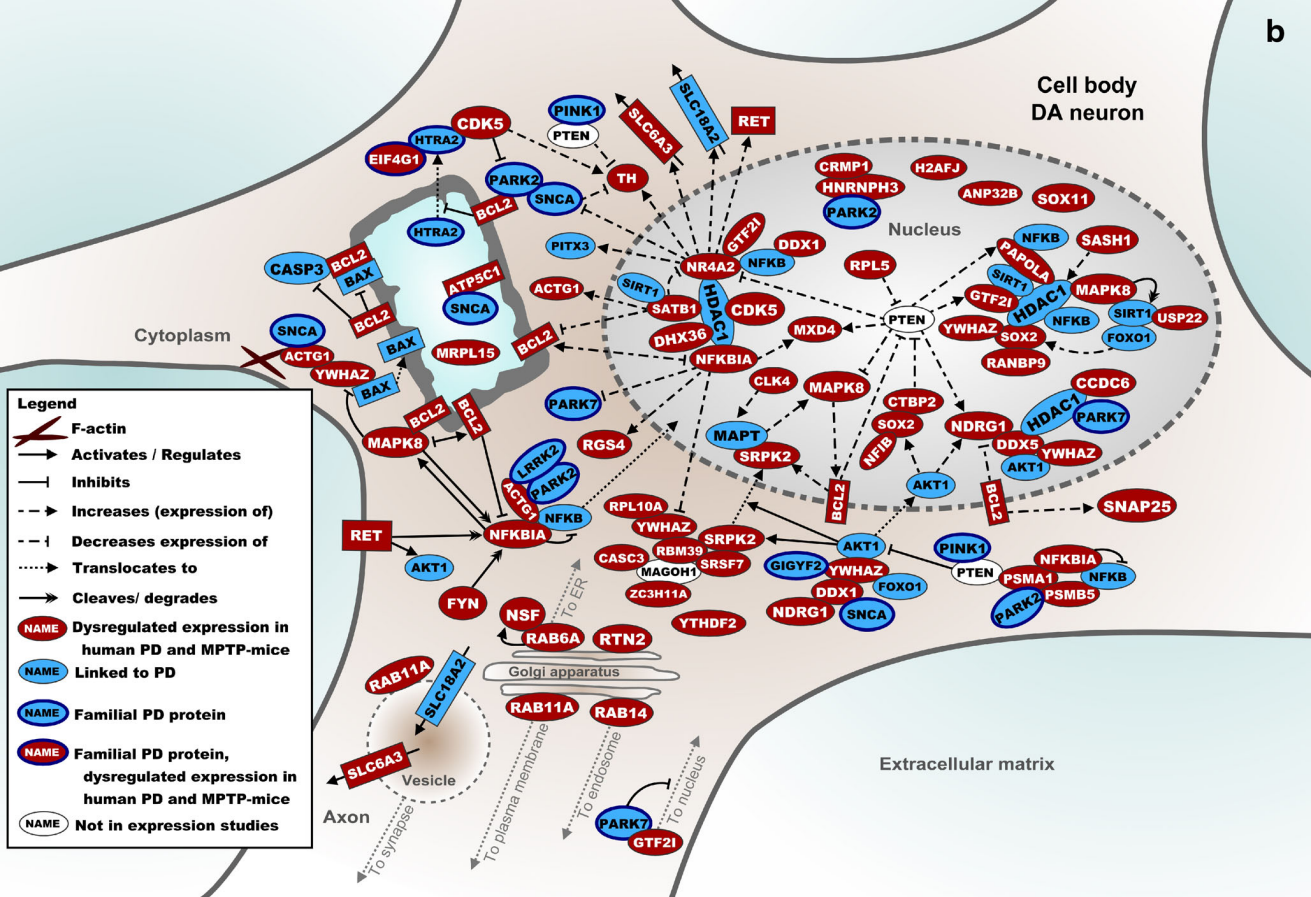
**a** Molecular landscape of interacting proteins, encoded by the mRNAs that are differentially expressed in the SN of *both* human PD patients and MPTP-treated mice, located primarily in the (pre) synapse and axon of the DA neuron. See text and Online Resource for details. **b** Molecular landscape of interacting proteins, encoded by the mRNAs that are differentially expressed in the SN of *both* human PD patients and MPTP-treated mice, located primarily in the cell body and nucleus of the DA neuron. See text and Online Resource for details

Figure 1 shows a molecular landscape of interacting proteins encoded by the mRNAs that are differentially expressed in the SN of *both* human PD patients and MPTP-treated mice. These proteins form signaling cascades that are located in the SN neuron presynapse, cell body, or nucleus. The main cascades in the presynaptic landscape regulate DA synthesis, autophagy, calcium signaling, vesicle trafficking, and exocytosis (Fig. 1a). In the cell body and nucleus, particularly mitochondrial (dys)function and transcriptional regulation through histone and nucleosome modification and its reciprocal effect on pre-mRNA splicing are present (Fig. 1b). More specifically, NR4A2 and SOX2, two of the five transcription factors that are required for a DA neuron-like expression pattern, bind to HDAC1, a histone deacetylase that interacts with many proteins in the landscape. Therefore, dysregulation of any of these processes affects DA neuron-specific expression and reduces the number of neurons with a DA phenotype. In the Online Resource, the landscape is described in full detail, and the current knowledge about the functions of all landscape proteins is summarized.

### Enrichment Analysis of Striatal mRNA Expression Data

#### Human PD

Similar to the SN data described above, the disease/disorder categories that are directly related to PD-like movement disorders and motor symptoms were significantly enriched within the mRNAs that were found to be differentially expressed in the striatum of PD patients (Table 3). Furthermore, the enriched functional categories were mainly related to neuronal functions such as (synaptic) transmission and molecular/metal ion transport.

**Table 3:** Gene enrichment analysis of the striatum

	MPTP mouse									Human PD			Overlap			
Rank	5 h	*p* value	#	24 h	*p* value	#	72 h	*p* value	#		*p* value	#		*p* value	#	Overlapping genes
Category: diseases and disorders																
1	Epileptic seizure	2.55E-21	21	Psoriasis	6.98E-14	39	Movement disorders	1.05E-09	40	Neurological signs	5.52E-28	64	Seizures	4.14E-03	5	CHGB, ENC1, KCNQ5, NPTX2, TGM2
2	Seizures	3.94E-18	25	Glucose metabolism disorder	3.37E-11	50	Schizophrenia	2.52E-08	25	Dyskinesia	5.52E-28	63	Abnormal secretion by adrenal gland	5.71E-03	2	CHGB, ITSN1
3	Epilepsy	7.14E-18	22	Inflammation of organ	3.98E-11	51	Amyloidosis	7.20E-08	25	Disorder of basal ganglia	8.34E-28	69	Epilepsy	9.93E-03	4	CHGB, ENC1, KCNQ5, NPTX2
4	Dyskinesia	1.07E-06	18	Vascular disease	7.16E-11	45	Dementia	2.41E-07	24	Chorea	7.07E-27	60	Dyskinesia	1.34E-02	5	CHGB, DIRAS2, FABP7, S100A10, TGM2
5	Endometriosis	2.70E-06	14	Inflammatory response	3.45E-10	37	Quantity of phagocytes	6.73E-07	20	Movement disorders	8.28E-27	79	Movement disorders	1.34E-02	6	CHGB, DIRAS2, FABP7, S100A10, TGM2, TMEM176B
Category: molecular and cellular functions																
1	Apoptosis	5.80E-08	39	Proliferation of cells	3.23E-17	115	Morphology of cells	6.61E-11	59	Neurotransmission	1.44E-13	35	Proliferation of endothelial cell lines	2.37E-02	2	ADAMTS1, ITSN1
2	Differentiation of cells	1.07E-06	31	Morphology of cells	7.55E-08	79	Organization of cytoskeleton	6.61E-11	45	Synaptic transmission	2.90E-11	29	Apoptosis of neuroblastoma cell lines	3.02E-02	2	ITSN1, TGM2
3	Proliferation of cells	1.89E-06	43	Necrosis	3.61E-15	91	Organization of cytoplasm	1.93E-10	46	Transport of molecule	3.95E-09	63	Cell death of cortical neurons	4.08E-02	2	ITSN1, TGM2
4	Cell death	2.58E-06	41	Apoptosis	2.05E-14	91	Formation of cellular protrusions	8.34E-10	33	Transport of metal ion	1.02E-08	24	Neuritogenesis	4.08E-02	3	DCLK1, ENC1, ITSN1
5	Cell cycle progression	4.27E-06	21	Cell movement	2.81E-14	77	Apoptosis	8.34E-10	67	Morphology of neurites	2.10E-08	17	Apoptosis of endothelial cells	4.34E-02	2	ADAMTS2, ITSN1

Ingenuity annotations of genes dysregulated by MPTP in the mouse striatum, after 5, 24, and 72 h between treatment and analyses (430 genes for time points combined), annotations of all dysregulated genes in the striatum of PD patients (259 genes), and those that are dysregulated in both the MPTP mouse model and human PD (i.e., 14 “overlapping” genes). Data are extracted from references in Table 1. The top 5 Ingenuity annotations of the categories “diseases and disorders” and “molecular and cellular functions” are displayed, as well as their respective *p* value and number of genes involved (#). All *p* values are corrected for multiple testing by the Benjamini-Hochberg false discovery rate. Categories with only one (target) gene were discarded. See text for further details

#### MPTP Mouse

Again, the most significantly enriched categories were determined at various time points following MPTP treatment. However, the respective study used a timing regimen different from the study focusing on the SN mentioned above. At 5 and 24 h after injection, the predominant functional categories were implicated in (neuronal) cell death and other acute, MPTP toxicity-related processes, including (dys)regulation of inflammatory responses and immunity-related cells, and endometriosis. At 72 h after MPTP injection, the most significantly enriched categories shift towards those enriched within the PD patient striatal data, i.e., categories related to PD-like motor symptoms and neuronal/synaptic function (Table 3).

#### Overlap Between Human PD and Mouse MPTP

The most significantly enriched categories within the mRNAs that were differentially expressed in the striatum of *both* human PD patients and the MPTP mouse model comprise a combination of the enriched “diseases and disorders” categories identified in human PD and MPTP-treated mice as summarized above, i.e., relating to both movement disorders and epilepsy (Table 3). Indeed, the “cellular and molecular functions” categories involved in both neuronal/synaptic function and neuronal cell growth/death are enriched within the overlapping PD patient/MPTP mouse striatal data.

### Molecular Landscape of Shared Processes in the Striatum

**Fig. 2:**
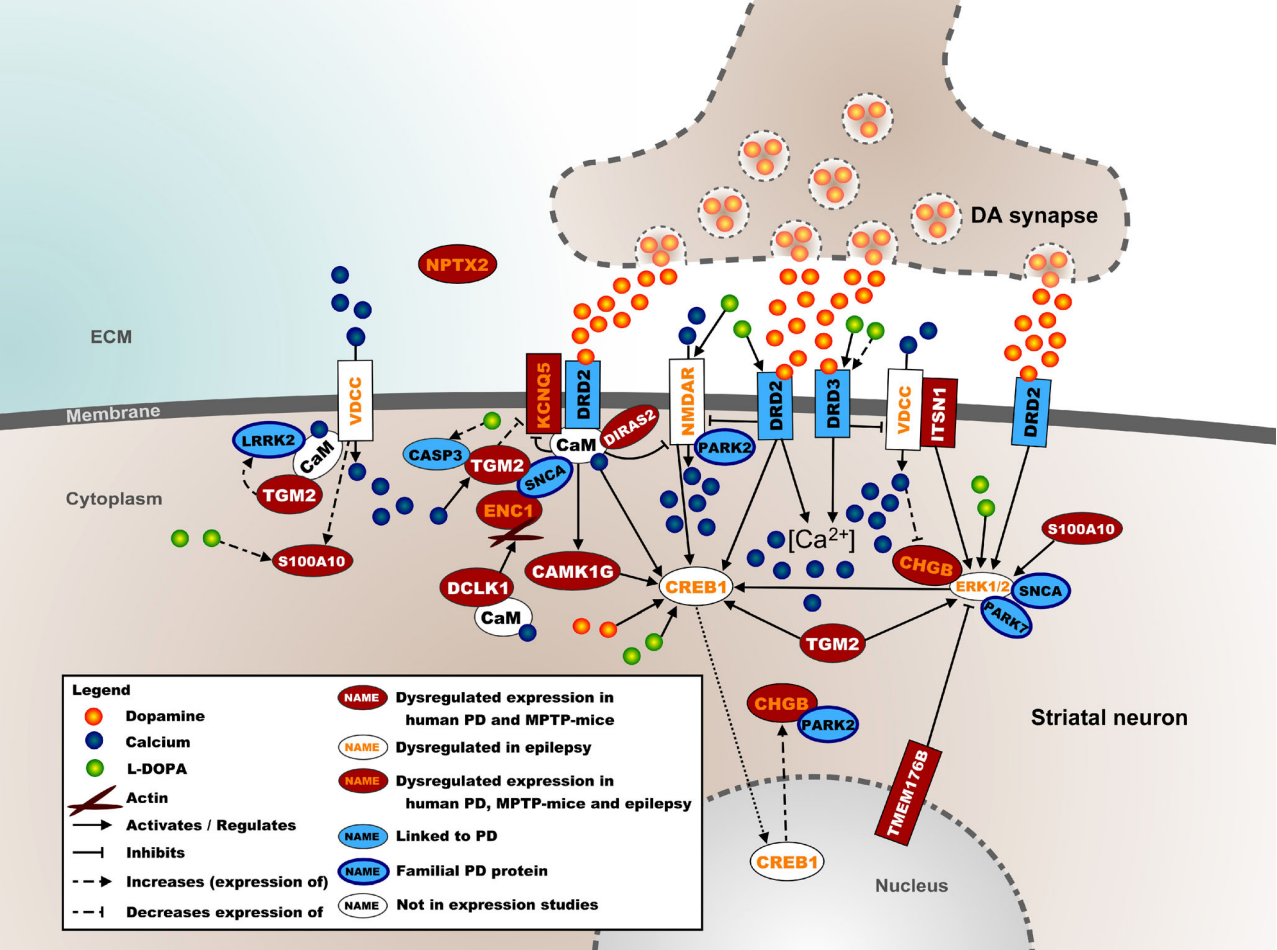
Molecular landscape of interacting proteins, encoded by the mRNAs that are differentially expressed in the striatum of *both* human PD patients and MPTP-treated mice located in the postsynapse of a striatal neuron. See text and Online Resource for details

Figure 2 shows a molecular landscape of interacting proteins encoded by the mRNAs differentially expressed in the striatum of *both* human PD patients and MPTP-treated mice. PD as well as MPTP treatment result in the degeneration of nigrostriatal DA neurons, which decreases DA release in the striatum and results in diminished activation of the DRD2 and DRD3 DA receptors, in turn affecting postsynaptic striatal protein expression and calcium signaling. Furthermore, the majority of the proteins in this landscape regulate CREB1—a transcription factor that is essential for DA-dependent gene expression in the striatum—either directly via calcium signaling or through activation of the ERK1/2 kinases. In the Online Resource, the landscape is described in full detail, and the current knowledge about the functions of the landscape proteins is presented.

## Discussion

This study aimed to determine the construct validity of the MPTP mouse as a model to study human PD pathogenesis. First, the most important dysregulated biological processes underlying both human PD and MPTP toxicity were identified by enrichment analyses of published genome-wide mRNA expression data from postmortem SN and striatum of PD patients and MPTP-treated mice. Second, proteins encoded by the mRNAs that were differentially expressed in both PD patients and MPTP-treated mice were integrated into molecular landscapes representing the main biological processes that are shared by human PD and mouse MPTP toxicity. Our findings demonstrate that, at the level of the SN, MPTP toxicity has substantial relevance for PD pathogenesis. This is less obvious for the striatum, in which important temporal effects of MPTP toxicity were noted.

Because categories related to basal ganglia-based motor dysfunction and neurodegeneration were enriched in the SN of both PD patients and MPTP-treated mice, the effects of MPTP toxicity on gene expression in the mouse SN appear to have similar phenotypic consequences as human PD. However, differences exist between PD and MPTP toxicity regarding the specific (dysregulated) biological processes involved. While in the SN of PD patients, enriched molecular and cellular functions relate to neuronal and synaptic functions, functional themes pertaining to cell growth and death predominate in the MPTP mouse model. This discrepancy could well reflect the differences between the protracted processes of neurodegeneration in PD, as well as simultaneous compensatory neuroplastic mechanisms, compared to the acute MPTP toxicity in mice. Moreover, the biological processes that overlap between the SN of both PD patients and MPTP-treated mice mainly relate to neuronal/synaptic function and (neuronal) cell death, while the molecular signaling cascades involved regulate DA synthesis and recycling, endocytosis and exocytosis of (DA-containing) synaptic vesicles, and cytoskeleton-dependent synaptic remodeling. These biological processes have been implicated in the pathogenesis of PD before [8–10]. Proteins encoded by other differentially expressed mRNAs are important players in other processes that have been implicated in DA neuronal dysregulation and death, including cytoplasmic and nuclear cascades regulating (vesicular) trafficking [11], mitochondrial function and apoptosis [12], proteosomal degradation (including the degradation of DA neuron-specific transcription factors) [13], as well as transcriptional, posttranscriptional and translational processes such as histone regulation [14] and pre-mRNA splicing [15].

As opposed to the SN findings, the categories that were most significantly enriched within the differentially expressed striatal mRNAs did not unequivocally overlap between human PD and mouse MPTP-induced toxicity. In PD, they are related to PD-associated motor symptoms, but in the MPTP-treated mouse striatum, the enriched categories depend on the length of the time period between MPTP treatment and transcriptional profiling. Early (i.e., 5 h) after MPTP injection, the most significantly enriched disease categories within the mouse striatal mRNA expression data are not related to motor dysfunction but to epilepsy. Epilepsy is a known acute side effect of MPTP injection in mice [16] and is directly linked to the temporary presence of the active MPTP metabolite MPP+ [17]. Although observational studies have reported an association between epilepsy and PD [18], an acute side effect of MPTP is more likely, as MPTP treatment does not seem to have long-lasting epileptogenic effects [19]. Indeed, in line with a gradual reduction of MPP+ levels in the mouse brain over time, at 24 h after injection, some of the significantly enriched disease categories point towards an inflammatory response, while at 72 h, they are related to motor dysfunction and neurodegeneration. A similar pattern is observed for the molecular and cellular functions, where at 5 h after injection, the enriched functions are mainly related to cell growth and death, shifting to cellular organization- and morphology-related functions at later time points. Although direct comparison of studies is challenging due to different injection regimens, these findings may suggest that in the striatum —more clearly than in the SN—the MPTP-induced expression changes and the molecular signaling cascades that are affected by these expression changes are not consistent but change towards more PD-relevant processes over time. The overlap between PD and MPTP-treated mice in biofunctions of striatal mRNAs, encompassing both “acute” toxicity-related and “chronic” PD-related categories, is corroborated by the molecular landscape. In this landscape, part of the affected biological processes and functions appears directly related to diminished DA striatal innervation through postsynaptic DA receptors. In addition, there seems to be a strong convergence on the regulation of intracellular calcium levels as well as CREB1-related signaling, which have both been functionally linked to epilepsy before [20]. Again, it remains unsure whether these signaling cascades are affected by the reduced DA innervation from the presynaptic SN neuron or that they merely reflect an acute “side effect” of MPTP rather than having much direct relevance for PD pathogenesis. The finding of the endometriosis-related enrichment in mice may be related to the fact that only female mice were used, while for the human studies, both male and female subjects were included. However, since 8 out of the 14 proteins with the annotation “endometriosis” are also present in the annotated category “dyskinesia”, the “endometriosis” enrichment may be due to either a true gender effect, an enrichment of genes involved in movement dysfunction-related processes, or both.

Thus, our transcriptome analyses and molecular landscapes indicate that the MPTP mouse constitutes a valid model for the chronic molecular and pathological changes that occur in the SN of PD patients and hence of the PD phenotype that is associated with these changes. However, this is less obvious for the striatum, because early after MPTP injection, enriched categories and functions encompass mainly processes that are not directly related to PD. It appears that human PD pathogenesis in the striatum is better recapitulated at the molecular level in the MPTP-treated mouse model 72 h postinjection and perhaps even later. Effects of the level of chronicity of the dosing regimen on markers of DA neurotransmission—e.g., TH expression and striatal DA levels—and behavioral outcome have been reported before [21–23], but specific effects on the transcriptome have not been addressed. In this respect, assessing the relationship between the temporal expression patterns in human PD patients and their disease duration would be of interest, similar to the analyses that were performed on the mouse-MPTP data. Unfortunately, for the reported human expression studies, disease duration data were not available for all cases.

In addition to the acutely toxic nature of early MPTP-induced pathology, there may be several other explanations for the apparent discrepancy between the striatal PD pathogenesis and MPTP toxicity. First, presynaptic DA denervation may result in enriched processes that are different between humans and mice due to species specificity. For example, similar degrees of DA degeneration in humans and mice do not result in similar phenotypic severity [24] and clinical phenotypes differ as mice do not show the tremor often seen in patients. Second, chronic compensating processes including adaptive neuroplasticity could play an important role in PD but less in MPTP-treated mice. In PD, these processes may be linked to the synaptic transmission- and molecular transport-related functions that are enriched in striatal mRNAs. The acutely toxic nature of MPTP would not allow for such an adaptation. Finally, it should be noted that, despite the high degree of overlap, the absolute number of differentially expressed mRNAs that overlap between human PD striatum and MPTP mouse striatum is low, perhaps prohibiting the detection of statistically relevant enrichment.

**Fig. 3:**
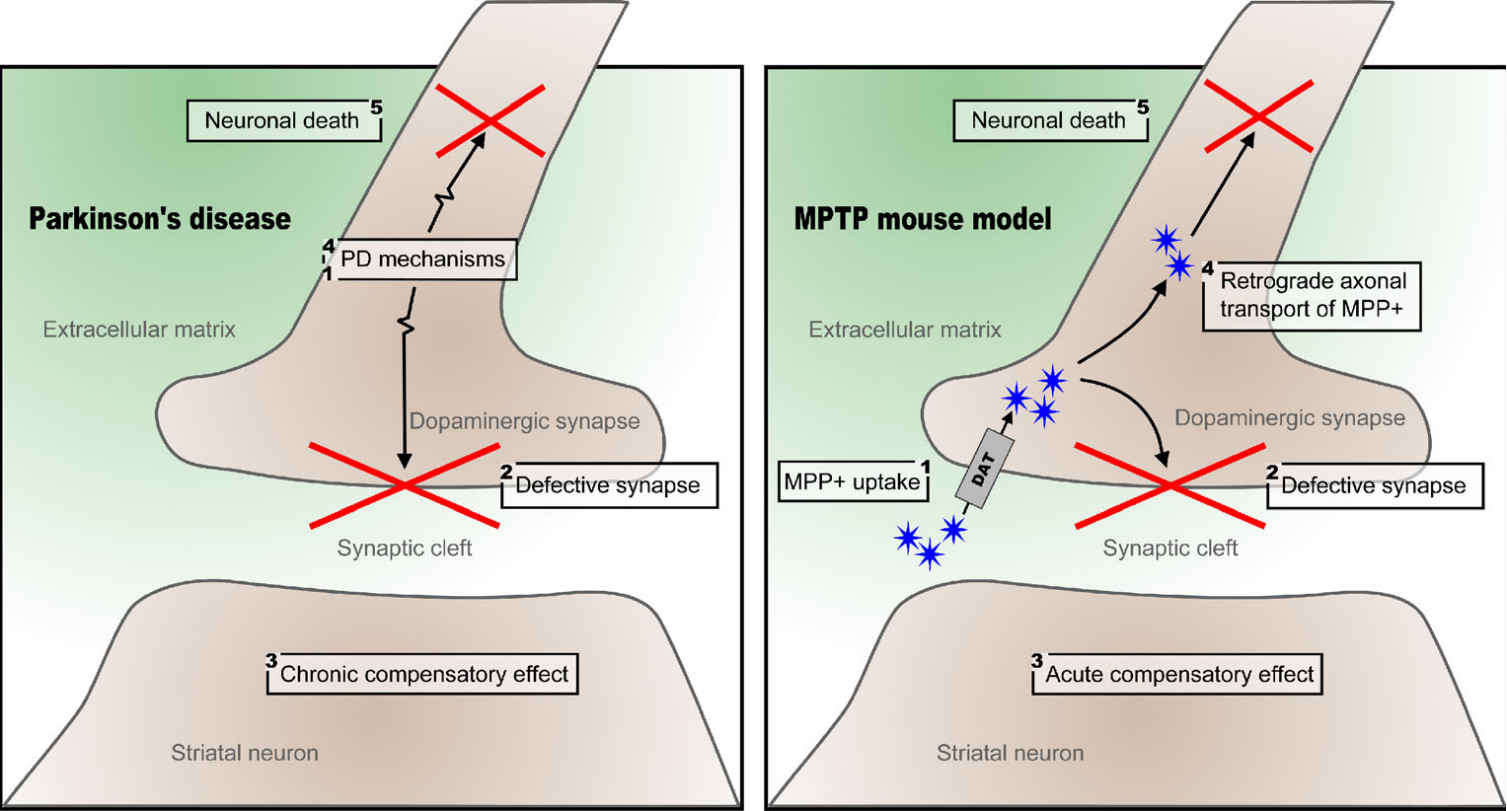
Proposed fundamental mechanisms underlying the degeneration of dopaminergic neurons in Parkinson’s disease and mouse MPTP toxicity. The *numbers* denote the sequence of events in Parkinson’s disease and the MPTP mouse, respectively. See text for further details

The principal differences between the chronological orders of events in PD-linked neurodegeneration versus MPTP-induced toxicity are summarized in Fig. 3. In PD, a number of molecular mechanisms in presynaptic SN neurons—including vesicular trafficking and exocytosis, mitochondrial apoptosis, as well as several transcriptional and translational processes—cause neuronal/synaptic dysfunction and cell death, which is followed by chronic, postsynaptic compensatory mechanisms in the striatum. In contrast, MPTP is taken up readily as MPP+ through the DA transporter (DAT) in presynaptic SN neuron terminals [25], causing toxicity and sequestration of MPP+ into synaptic vesicles [26]. MPP+ also reaches the cell body of presynaptic SN neurons through retrograde axonal transport [27], which in turn causes neuronal death through mitochondrial accumulation and electron transport chain inhibition, inducing neuronal apoptosis [28]. This relatively rapid cell death causes more acute compensatory effects in the postsynaptic striatal neurons [29].

Together, our findings provide further evidence that the molecular changes in the SN of MPTP-treated mice correspond to the observed alterations in the SN of PD patients. However, for a proper reflection of the molecular changes occurring during PD pathogenesis in the striatum, the time point of studying the changes following MPTP treatment is crucial. Further transcriptome studies are needed to determine whether waiting longer than 72 h would indeed provide a better construct validity for human PD pathogenesis and whether there is an optimal time frame following MPTP injection to assess striatal PD pathology in the MPTP mouse model. Such knowledge will have important practical implications for the use of the MPTP mouse as a model for PD and for PD drug testing.

## Electronic Supplementary Material

Below is the link to the electronic supplementary material.

ESM 1 (PDF 346 kb)
