# Supplementary material for: Validity of the MPTP-Treated Mouse as a Model for Parkinson’s Disease
Source: Mol Neurobiol. 2015 Feb 13;53(3):1625–36. doi: 10.1007/s12035-015-9103-8 (PMC4789200; doi:10.1007/s12035-015-9103-8)
Supplement: Supplementary file 1 — (PDF 346 kb) [file 12035_2015_9103_MOESM1_ESM.pdf]

ONLINE RESOURCE

Validity of the MPTP-treated mouse as a model for Parkinson's disease

Cornelius J.H.M. Klemann, M.Sc. [1], Gerard J.M. Martens, Ph.D. [1], Geert Poelmans, M.D., Ph.D. [1,2,3 *], Jasper E. Visser, M.D., Ph.D. [1,4,5 *]

[1] Department of Molecular Animal Physiology, Donders Institute for Brain, Cognition and Behaviour, Radboud University, Nijmegen, The Netherlands

[2] Department of Cognitive Neuroscience, Donders Institute for Brain, Cognition and Behaviour, Radboud University Medical Center, Nijmegen, The Netherlands

[3] Department of Human Genetics, Radboud University Medical Center, Nijmegen, The Netherlands

[4] Department of Neurology, Donders Institute for Brain, Cognition and Behaviour, Radboud University Medical Center, Nijmegen, The Netherlands

[5] Department of Neurology, Amphia Hospital, Breda, The Netherlands

[*] Equal contribution

Corresponding author: Jasper E. Visser, MD PhD
Department of Neurology
Radboud University Medical Center
P.O.Box 9101
6500 HB Nijmegen, The Netherlands
Telephone: +31 (0)24 361 66 00
Fax: +31 (0)24 354 11 22
Email: jasper.visser@radboudumc.nl

FIGURE DESCRIPTIONS

Parkinson's disease (PD) as well as 1-methyl-4-phenyl-1,2,3,6-tetrahydropyridine (MPTP) treatment result in the degeneration of dopaminergic neurons in the substantia nigra (SN), leading to a decrease of dopamine (DA) release to the striatum. Below, the molecular landscapes of biological processes shared between PD and MPTP treatment in the substantia nigra (SN) (Figures 1a and 1b) and striatum (Figure 2) are described in full detail, and the current knowledge about the functions and interactions of all landscape proteins is presented. In these descriptions, proteins that appear in **bold** are dysregulated in both human PD and the MPTP mouse model. Underlined proteins are associated with PD through either expression or genetic data from PD patients, and familial PD proteins are double underlined.

Detailed description of the biological processes depicted in Figure 1A. Molecular landscape of interacting proteins, encoded by the mRNAs that are differentially expressed in the SN of *both* human PD patients and MPTP-treated mice, located primarily in the (pre) synapse and axon of the DA neuron.

DA synthesis

TH catalyzes the rate-limiting step in DA synthesis, i.e. the conversion of the amino acid L-tyrosine into L-3,4-dihydroxyphenylalanine (L-DOPA)[1,2]. **TH** expression is increased by the receptor tyrosine-protein kinase **RET**[3] and **SLC6A3**[4]. **TH** is activated by the adaptor protein **YWHAZ**[5,6] and the cyclin-dependent kinase **CDK5**[7], and DA itself inhibits **TH** activity in a negative feedback loop[8,9]. **YWHAZ** also binds to the kinases **MARK2**[10], involved in microtubule regulation[11], and **PDXK**[12], required for the synthesis of pyridoxal-5-phosphate (PLP) from vitamin B6[11]. PLP, in turn, is an essential cofactor for the conversion of L-DOPA into DA by dopa decarboxylase[13].

(DA) release

Soluble NSF Attachment Protein Receptor (SNARE) proteins form a complex that is required for synaptic vesicle docking and subsequently the release of their contents (e.g., DA) into the synaptic cleft[11]. **SNAP25** and **VAMP2**, core components of the SNARE complex, physically interact with each other[14] and form a complex together with **STXBP1**[15], a protein that is also involved in synaptic vesicle fusion and docking[11]. **STXBP1** binds to **CDK5**[16], a protein involved in cytoskeleton regulation, synapse plasticity, exocytosis and endocytosis (see more on **CDK5** below). The vesicle fusion ATPase **NSF** increases the dissociation of the SNARE complex (dissociation of **SNAP25** and **VAMP2**) and thereby enables the vesicle to fuse with the plasma membrane[17]. **NSF** binds to **YWHAZ**[18] and AKT1[19], and inhibits CASP3 activity[20].

The DA – acetylcholine (ACh) balance may be involved in PD pathology[21] and ACh regulates DA release[22]. The extracellular protein **ACHE** hydrolyzes ACh that is released into the synaptic cleft[11]. Although **ACHE** does not directly interact with other landscape proteins, its function may well be linked to PD since its activity is reduced in the cerebral cortex and the medial occipital cortex of PD patients[23]. Moreover, **ACHE** is inactivated by MPTP[24] and **ACHE** deficiency is neuroprotective in the MPTP mouse model[25].

DA reuptake

In response to an action potential, DA is released from vesicles in the synaptic cleft by exocytosis and reuptake of extracellular DA occurs by **SLC6A3** to terminate the signal[26,27]. **SLC6A3** binds to the familial PD proteins PARK2[28] and SNCA[29], which both enhance its activity. PARK2 and **RET** increase the expression of **SLC6A3**[30,28,3], while SNCA increases **SLC6A3** translocation to the plasma membrane[29]. In addition to increasing the expression of **TH**, **SLC6A3** activates AKT1[31] and both inhibits the activity[32] and decreases the expression of **CDK5**[33]. Cytoplasmic DA (either synthesized by **TH** or after reuptake by **SLC6A3**) is transported into cytoplasmic vesicles by SLC18A2[11]. These vesicles are subsequently translocated via axoplasmic transport to the synapse for exocytosis.

Recycling of membrane-bound synaptic components

To further control DA levels in the synaptic cleft, proteins including **SLC6A3**, DRD2 and the voltage-dependent calcium channel (VDCC) are endocytosed, transported to the endosome and either recycled back to the membrane or degraded. **ATP6V0D1** is a subunit of the proton V-ATPase and increases the acidity of the endosome[11], which is required for a normal endosomal function. DRD2 binds to **SLC6A3**[34] and increases its localization in the plasma membrane[35], and activates the transport activity of **SLC6A3**[36]. **SNAP25** is also involved in this process, by binding to **SLC6A3**[37] and VDCC[38,39]. Correct regulation of the recycling and degradation of DRD2 and **SLC6A3** is essential for normal neuronal DA signaling and deregulation of the proteins that regulate their endocytosis and/or expression at the plasma membrane can disturb this signaling. The G protein **GNAI2** regulates the expression of, binds to, and prevents the plasma membrane translocation of, DRD2[40]. **RGS4** binds to both DRD2[41] and **GNAI2**[42], and increases the GTPase activity of **GNAI2** (not shown)[43]. In turn, **GNAI2** increases the recruitment of **RGS4** to the plasma membrane (not shown) [44], where it binds – among others – to **LPAR1**[41]. The Rab GTPases **RAB4A**, **RAB6A**, **RAB11A** and **RAB14** are also involved in (vesicular) recycling and endosomal function; see the section '(Vesicle) trafficking and exocytosis' in the detailed description of figure 1b below for their functions within the landscape.

Cytoskeleton / cytoplasmic cascades

Recycling, trafficking and exocytosis require cytoskeletal regulation. A substantial number of landscape proteins control cytoskeleton dynamics, e.g., by regulating RAC1 activity. RAC1 is activated by **FRAT2**[45], **TNK2**[46], **FYN**[47], **DLK1**[48], **RET**[49,50], **LPAR1**[51], and **KIFAP3**[52], and inhibited by **CDK5**[53] and **CRMP1**[54]. Further, RAC1 binds to **RBM39**[55] and **RAP1GDS1**[56]. The latter also binds to **KIFAP3**[57] and regulates the GDP/GTP exchange of GTP-binding proteins such as RAC1[58]. RAC1 has multiple functions. First, it increases the expression of **CDH2**[59] and activates **MAPK8**[60], a serine/threonine-protein kinase that is involved in cell proliferation, differentiation, migration, transformation and programmed cell death (see also the section 'Mitochondrial function and apoptosis' in the description of figure 1b for the role of **MAPK8** in apoptosis)[11]. **MAPK8** binds to MAP3K7[61], CASP3[62], **mTORC1**[63] and **KLC1**[64]. Apart from RAC1 activation, **MAPK8** is also activated by **MAGED1**[65], **GNAI2**[66], **MAP2K4**[67], **RET**[49], **LPAR1**[68] and DA[69], and inhibited by AKT1[70]. **MAGED1** also binds to MAP3K7[71] and **ATXN10**[72], and activates CASP3[65]. **MAPK8** also activates CASP3[73,74] and **mTORC1**[63], and inhibits AKT1[75,76]. **MAP2K4** is an essential part of the MAP kinases signaling pathway and is bound to the phosphatase **DUSP19**[77], AKT1[70] and MAP3K7[78], whereas AKT1 inhibits[70] and MAP3K7 activates **MAP2K4**[79]. Multiple proteins in the

landscape regulate AKT1. AKT1 binds to **NSF** and **MAP2K4**, as mentioned above, as well as to **YWHAZ**[80] and **MARK2**[81], is activated by **SLC6A3** (see above), **YWHAZ**[82], **GNAI2**[83], **LPAR1**[68], **TNK2**[84] and **CDH2**[85], and inhibited by **MAPK8** (see above), **RGS4**[86] and **DLK1**[87]. Second, RAC1 increases the polymerization of globular actin(**ACTG1**)[88] to filament actin (F-actin)[11]. **ACTG1** binds to **CTBP2**[89], MAP3K7[90] and **NDRG1**[91] (see Figure 1b and its description for more **ACTG1** interactions). Moreover, **CDH2**[92], **GNAI2**[93] (not shown), **VSNL1**[94] and the **NMDAR**[95] bind to actin, and **CDK5** increases actin polymerization[53].

Microtubule-dependent trafficking

In addition to RAC1, **FYN** is a non-receptor tyrosine-protein kinase that is involved in cell growth and survival, cell adhesion, cytoskeletal remodeling and axon guidance[11]. **FYN** is activated by **L1CAM**[96] and is itself an activator of RAC1 (see above), **TNK2**[97] and CASP3[98]. **FYN** is cleaved by CASP3[99,100]and binds to **TNK2**[97], **GABRG2**[101] and MAPT[102]. Like **FYN, TNK2** is a non-receptor tyrosine-protein kinase and is involved in cell survival, proliferation and endocytosis[11], whereas **GABRG2** is a subunit of the GABA receptor and regulates neuronal inhibition[11]. Moreover, **FYN** phosphorylates MAPT[103,104], which is a susceptibility gene for idiopathic PD[105-109] that promotes assembly and stability of microtubules[11]. Microtubule-dependent trafficking is affected in PD and, among others, affects axonal transport of autophagosomes that contain damaged mitochondria and aggregated proteins, which can lead to SNCA accumulation and synaptic dysfunction[110,111]. In the landscape, MAPT binds to **MARK2**[112], **YWHAZ**[10], **KLC1**[113], **CDK5**[114], **STXBP1**[16] and **PPP2R2A**[115]. In addition, MAPT is phosphorylated by **CDK5**[116] and **FYN**[104], whereas **PPP2R2A** dephosphorylates MAPT[117]. Furthermore, **MARK2** phosphorylates both MAPT and **MAP4** which causes microtubule detachment and disassembly[11]. **CHP1** binds to microtubules and mediates the binding of the endoplasmic reticulum (ER) and the Golgi apparatus with microtubules (not shown)[11]. Other proteins in the landscape that bind microtubules are **MAPRE2** (not shown)[118], **NDRG1** (not shown)[119], **KLC1** (not shown)[11] and **MAP4** (not shown)[120]. **KLC1** is a kinesin that regulates microtubule-associated transport of organelles[11] and **MAP4** promotes the assembly of microtubules[121]. In addition, **MAPK8** is known to increase microtubular assembly[122,123].

Cell adhesion

The proteins **L1CAM**, **FYN**, **CDH2**, **CDH8** and **RET** regulate cell adhesion[11]. **L1CAM** regulates neuron-neuron adhesion and is found in axon terminals[11] and **FYN** regulates synapse formation[124]. The cadherins **CDH2** and **CDH8** are calcium-dependent adhesion molecules[11]. **CDH2** binds **KIFAP3**[125], **NMDAR**[95] and actin[92]. Cleavage of **RET** by caspases results in a fragment that functions as a cadherin accessory protein that potentiates cadherin-mediated cell aggregation[126].

Autophagy

As indicated above, microtubule-dependent trafficking is necessary for the transport of autophagosomes and degradation of their cargo. Autophagy dysregulation is also directly implicated in PD via the familial PD proteins[127]. In the landscape, **MAPK8**, **CDK5** and **AKT1S1** (**mTORC1**) regulate autophagy, e.g., **MAPK8** phosphorylates **BCL2**, which induces dissociation of **BCL2** from Beclin-1 and autophagy activation (not shown)[128]. In addition, **mTORC1** binds to[63], and is activated by, **MAPK8**[63], and has been shown to inhibit autophagy[129,130]. Furthermore, **mTORC1** binds to **PPP2R2A**[131], **YWHAZ**[12] and **RPL5**[132], whereas **YWHAZ** and **RPL5** also bind to each

other[12]. Lastly, the kinase **CDK5** has been shown to be required for autophagy in multiple PD models[133-135].

Calcium

Calcium channels regulate neuron excitability and release of neurotransmitters such as DA. In PD, nigral DA neurons show a dysregulation of calcium[136]. In the landscape, calcium is transported into the cell by the **NMDAR**[137] and the VDCC[138], and transported out of the cell by **SLC8A1**[139,140]. The **NMDAR** binds to **CDH2**[95]and the VDCC binds to **SLC8A1**[141,39], **TSPAN7**[142], **MAP4**[39], **MARK2**[39], **SLC4A3**[39], **CRMP1**[142], **PFKM**[39], **RAB14**[39] and, as already mentioned above, **SNAP25**[38,39]. These proteins have a wide range of functions. **SLC8A1** rapidly transports Ca2+ out of the cell to prevent overloading of intracellular stores[139]. **TSPAN7** is a surface glycoprotein that may have a role in neurite outgrowth[143]. **MAP4** and **MARK2** regulate microtubular dynamics (see above). **SLC4A3** is an anion exchanger that exchanges HCO3$^-$ for Cl$^-$ and thereby regulates the intracellular pH[11]. Another protein in the PD landscape that regulates neuronal pH by transporting HCO3- into the cell is **SLC4A8**[11]. **CRMP1** regulates remodeling of the cytoskeleton[11]. **PFKM** binds to **YWHAZ**[18] and catalyzes the conversion of D-fructose 1,6-phosphate to D-fructose 1,6-biphosphate[11]. Binding of D-fructose 1,6-bisphosphate to soluble Fe2+ prevents its conversion to the insoluble Fe3+, an oxidation step that produces oxygen radicals. The availability of D-fructose 1,6-biphosphate may therefore affect iron content and oxygen radical levels[144] in the SN of PD patients. Lastly, **RAB14** and **SNAP25** are involved in intracellular trafficking (see above). Furthermore, VDCC function and thus calcium influx is inhibited by **CDK5**[145,146]. **LPAR1** increases calcium mobilization in the cytosol[147], whereas **RGS4** and **RGS7** both decrease mobilization of calcium[148,149]. In addition, **RAB4A** and **RAB11A** (not shown) increase the intracellular calcium concentration[150]. Calcium in turn activates **MAPK8**[151], **FYN**[96] and **VSNL1**[152,153], and inhibits **NSF**[154]. Moreover, calcium increases the expression of **NDRG1**[155] and binds to **VSNL1**[156], **CHP1** (not shown)[157], **CDH2**[11] and **CDH8**[11].

Familial PD proteins

The familial PD proteins have many interactions with components within the landscape. SNCA, the primary component of Lewy bodies in PD DA neurons, binds to **SLC6A3**[29], **TH**[158], **YWHAZ**[159], **MARK2**[159], **MAPK8**[160], **KLC1**[161], **MAP4**[159], **ATP6V0D1**[159], **FYN**[162,163], **STXBP1**[159], **SNAP25**[164] and **VAMP2**[165]. Further, SNCA activates **SLC6A3**[29], decreases **TH** expression (not shown)[166], inhibits **TH**[158,167] and **MAPK8**[168], and is inhibited itself by **FYN**[162]. Interestingly, SNARE (**SNAP25** and **VAMP2**) dysfunction results in mislocalization and accumulation of SNCA and could be an important pathomechanism of PD[169], which emphasizes the importance of the normal functioning of the SNARE complex. Furthermore, binding of PARK7 to **VAMP2**[170] and of LRRK2 to **NSF**[171] shows that other familial PD proteins also have a direct impact on SNARE complex function. LRRK2 also binds to **MAP2K4**[172], **GNAI2**[173] and **YWHAZ**[174], and activates AKT1[175]. PARK2, UCHL1, EIF4G1 and PINK1 are four other familial PD proteins that have interactions with proteins in the landscape, i.e., PARK2 binds to and is phosphorylated by **CDK5**[176], binds to **SLC6A3**[28] and **ACTG1**[177], inhibits **MAPK8**[178], and increases expression of **SLC6A3**[30]. UCHL1 binds to AKT1[19], SNCA[179], **RANBP9**[180], **mTORC1**[181] and **EIF1B**[72], while EIF4G1 binds to **YWHAZ**[182] and **MARK2** binds to[183], and activates, PINK1[183].

Detailed description of the biological processes depicted in Figure 1B. Molecular landscape of interacting proteins, encoded by the mRNAs that are differentially expressed in the SN of *both* human PD patients and MPTP-treated mice, located primarily in the cell body and axon of the DA neuron.

Transcriptional and translational regulation

Histone regulation. HDAC1 deacetylates core histones and thereby represses gene transcription[11]. HDAC1 expression is increased in the SN of PD patients[184] and is one of the central proteins in the landscape of SN mechanisms overlapping between PD and MPTP-treated mice. HDAC1 expression is increased by **SASH1**[185] and HDAC1 binds to **CDK5**[11], **DHX36**[186], **CCDC6**[187], PARK7[188], **MAPK8**[189], **PAPOLA**[190], the transcriptional regulators SIRT1[191], **GTF2I**[192], **DDX5**[193] and **NFKBIA**[194], and the transcription factors **SOX2**[195], **NR4A2**[196], **SATB1**[197] and NFKB[198]. HDAC1 itself activates AKT1[199], decreases the expression of BAX[200] and binds to the promoters of the genes encoding **SLC8A1**[201] and **TH**[196]. The HDAC1-associated transcriptional regulators SIRT1, **GTF2I**, **DDX5** and **NFKBIA** have multiple other landscape interactors. Variants in the SIRT1 gene promoter contribute to PD risk[202], and SIRT1 deacetylates HDAC1 and thereby increases its enzymatic activity (not shown)[191]. Further, SIRT1 binds to **USP22**[203,204], a histone deubiquitination protein that inhibits SIRT1 degradation[204] and, by removing ubiquitin from H2A and H2B, functions as a coactivator of histones[11]. Furthermore, SIRT1 binds to **SATB1**[205], **PAPOLA**[190], **GTF2I**[206], **MAPK8**[207] and the PD-associated[208]FOXO1[209]. SIRT1 inhibits FOXO1 (not shown)[210], whereas FOXO1 increases SIRT1 expression[211]. Further, **MAPK8** increases the degradation of SIRT1[212]. The familial protein PARK7 binds in the cytoplasm to **GTF2I** and thereby prevents its translocation to the nucleus in which **GTF2I**[213] is together with HDAC1 part of the deacetylation complex[11].In addition to binding to HDAC1, **DDX5** binds to **NDRG1**[91], AKT1[214] and **YWHAZ**[182]. Expression of NFKB is increased in the PD brain[215]. **NFKBIA** binds to NFKB and thereby prevents its activation and translocation to the nucleus[216]. **NFKBIA** degradation is increased by **RET**[217], **FYN**[218] and **MAPK8**[219], and inhibited by **BCL2**[220]. Increased degradation or inhibition of **NFKBIA** increases NFKB activation and translocation to the nucleus[216]. **NFKBIA** binds to **ACTG1**[90] and **PSMA1**[221], and activates **MAPK8**[222]. Further, **NFKBIA** increases the expression of the transcriptional repressor **MXD4**[223] and **RGS4**, a regulator of G proteins[224]. Furthermore, **NFKBIA** decreases the expression of the familial PD protein PARK7[223] and of adaptor protein **YWHAZ**[225]. Regulation of the expression by **NFKBIA** is probably an indirect effect of its inhibitory function on the NFKB complex. Like **NFKBIA,**NFKB binds also to **ACTG1**[90], **PSMB5**[90], **PAPOLA**[90] and **DDX1**[90]. HDAC1 deacetylates the NFKB subunit RELA and in this way inhibits the transcriptional activity of NFKB[11].

In addition to binding to **NFKBIA** and NFKB (see above), **ACTG1** also binds to LRRK2[173], PARK2[177], SNCA[159] and **YWHAZ**[182]. The adaptor protein **YWHAZ** binds (in addition to the proteins mentioned above) to the 60S ribosomal protein **RPL10A**[12], the ATP-dependent RNA helicases **DDX1**[12] and **DDX5**[182], GIGYF2[12], AKT1[80] and FOXO1[226]. GIGYF2, AKT1 and FOXO1 are all associated with PD[227,228,208] and dysreglation of **YWHAZ** may interfere with their function. On its turn, **DDX1** binds to **NDRG1**[91] and SNCA[229] and acts as a coactivator to enhance NFKB-mediated transcriptional activation[11].

*DA neuron signature.***NR4A2** and **SOX2** are important transcription factors for establishing and maintaining a DA-neuron-like expression pattern,[230] as is also apparent from their requirement for reprogramming fibroblasts towards a dopaminergic phenotype[231].
NR4A2 increases the expression of **TH**[232], **SLC6A3**[232], PITX3[232], **RET**[233] and SLC18A2[232], and decreases the expression of SNCA[234]. In addition to binding to HDAC1, **NR4A2** binds to **GTF2I**[235] and NFKB[236]. **SATB1** decreases expression of **NR4A2**[237] and increases the expression of **ACTG1**[237]. The HDAC1-binding **SOX2** (see above)[195] also binds to **YWHAZ**[238], **RANBP9**[238], **CTBP2**[195] and **NFIB**[195], and its expression is increased by AKT1[239] and FOXO1[240].

Other transcriptional regulators. PTEN is a phosphatase that dephosphorylates PIP3 to PIP2 and hence inhibits AKT1 signaling[241,11]. **RET** activates AKT1[242] that subsequently translocates to the nucleus[11] and increases the expression of **NDRG1**[243] and **SOX2** (see above)[239]. Furthermore, PTEN binds to and activates the familial PD protein PINK1[244] and affects the expression of multiple proteins in the landscape by increasing the expression of **PAPOLA**[245], **GTF2I**[245], **MXD4**[245] and **NDRG1**[246], and decreasing the expression of **NR4A2**[247], **MAPK8**[248] and **TH**[247]. The expression of PTEN itself is decreased by the 60S ribosomal protein **RPL5**[249] and the transcriptional repressor **CTBP2**[250].

Alternative pre-mRNA splicing. The polymerase **PAPOLA** creates the 3'-poly(A) tail of mRNAs[11], is required for endoribonucleolytic cleavage at poly(A) sites[11] and binds to HDAC1 (see above). **YTHDF2** has also a role in mRNA stability and splicing, by binding to N6-methyladenosine[11]. Of note, multiple other proteins involved in mRNA splicing are dysregulated in both human PD and the MPTP mouse model. MAGOH1 and **CASC3** are core components of the exon junction complex that is deposited at splice junctions on mRNAs, regulating mRNA splicing, nuclear export, cellular localization and translation efficiency[11]. MAGOH1 binds to **CASC3**[251], **ZC3H11A**[251], **SRSF7**[251], **RBM39**[251], and **SRPK2** (not shown)[252]. **RBM39** also binds to **SRSF7**[253] as well as **YWHAZ**[182] and **SRPK2**[254]. **SRPK2** is required for spliceosome complex formation[255] and, together with MAGOH1 and **RBM39**, binds to **SRSF7**[252] and MAPT[256], and increases the phosphorylation of **RBM39** (not shown)[252], **SRSF7** (not shown)[252] and MAPT (not shown)[256]. Phosphorylation of **SRPK2** at Thr-492 by AKT1 promotes its nuclear translocation and enhances its activity[11]. Like **CASC3**, MAGOH1 and **SRPK2, RBM39** and **SRSF7** are involved in pre-mRNA splicing[257,258]. For instance, **SRSF7** is involved in mRNA export out of the nucleus[259] and is known to prevent splicing of exon 10 of MAPT (not shown)[260]. **CLK4** phosphorylates proteins of the spliceosome complex[11] and regulates the alternative splicing of MAPT[261]. MAPT itself increases the expression of **MAPK8**[262].
Other proteins that also affect alternative splicing and are involved in nucleosome/ histone regulation are **HNRNPH3**, **CRMP1**, **H2AFJ** and **ANP32B**. **HNRNPH3** associates with pre-mRNA in the nucleus[11], and binds to PARK2[263] and **CRMP1**[264]. **H2AFJ** is a H2A histone variant and core component of the nucleosome[11] and **ANP32B** stimulates core histones to assemble into a nucleosome[11]. Nucleosomes define the exon-intron border and since pre-mRNA splicing occurs co-transcriptionally, nucleosome organization, transcription elongation rate or epigenetic marks can affect pre-mRNA splicing[265,266]. Moreover, histone deacetylation by HDAC1 affects pre-mRNA splicing, resulting in local repression of transcription[267,268,265]. HDAC1 is up-regulated in the SN of human PD patients and interacts with multiple proteins in the landscape (see also above).
Taken together, the central position of HDAC1 and the occurrence of multiple proteins involved in

histone regulation and pre-mRNA splicing in the SN landscape suggest that dysregulation of nucleosome organization and the splicing machinery are important factors in the biological processes that overlap between PD and the MPTP mouse model.

(Vesicle) trafficking and exocytosis

In Figure 1a, the involvement of the SNARE complex in (DA) exocytosis is shown, however, the SNARE complex also regulates intracellular transport, as is apparent from the binding of **SNAP25** to both **NAPB**[269] and **KLC1**[270]. **NAPB** is required for vesicular transport between the ER and the Golgi apparatus[11], and **KLC1** is a microtubule-associated protein that regulates the transport of organelles such as mitochondria. Like the SNARE complex, the familial PD protein SNCA may be involved in DA release and transport[11], but also in ER-to-Golgi vesicle trafficking[271,272]. SNCA modulates vesicle trafficking by binding to RABAC1 (not shown)[273], a protein that regulates the interaction between Rab GTPases and the SNARE complex[274]. Overexpression of SNCA disrupts vesicle trafficking and increases accumulation of vesicles in the cytoplasm[273]. Four Rab GTPases (**RAB4A**, **RAB6A**, **RAB11A** and **RAB14**) are overlapping between PD and the MPTP mouse. These proteins are involved in vesicular trafficking between compartments of the cell. **RAB4A** regulates localization of **VAMP2** to early endosomes and vesicles[275] and the membrane-bound form of **RAB4A** binds to **NDRG1**[276], a protein that is required for vesicular recycling[11]. **NDRG1** binds to actin filaments by binding to **ACTG1**[91] as well as to **ACOT7**[277] and **PPP2R2A**[91], and activates CASP3[278]. The RAB proteins **RAB6A**, **RAB11A** and **RAB14** are located in the Golgi complex and regulate protein trafficking to other organelles and the plasma membrane of the cell. Dysfunctioning of these proteins results in defective protein trafficking and membrane fusion, which can result in protein aggregation. **RAB6A** is located at the Golgi[279] and regulates vesicular transport from early and recycling endosomes to the Golgi (not shown)[280] but also transport from the Golgi to the ER[281]. Furthermore, **RAB6A** affects release of the SNARE (**SNAP25** and **VAMP2**) complex, which itself is involved in membrane fusion (see also Figure 1a) by binding and activating **NSF**[282]. **RAB11A** is located in recycling endosomes, the Golgi complex and on the cytoplasmic side of cytoplasmic vesicles, and regulates transport from the Golgi to the endosome[283] and from the Golgi to the plasma membrane[283]. **RAB11A** binds to the neuronal cell adhesion protein **L1CAM**[284] and therefore is probably involved in its trafficking. The RAB protein **RAB14** regulates vesicular transport between the Golgi and early endosomes, and is involved in **CDH2** shedding (not shown)[285] and as such affects cell-cell adhesion (not shown)[285]. Lastly, also the ER-shaping protein **RTN2**[286] is involved in vesicular ER to Golgi transport[287].

Dysregulated (vesicle) trafficking affects exocytosis, receptor trafficking, (membrane) recycling and ultimately decreases the viability of the neuron.

Proteasomal degradation

The proteasome is involved in removal of unwanted, damaged or aggregated proteins[288,289]. **PSMA1** and **PSMB5** are both part of the 26S proteosomal complex[290], bind to each other[291] and both bind to PARK2[292,293]. Moreover, **PSMA1** binds to PTEN[294] and **NFKBIA**[221], whereas **PSMB5** binds to the NFKB complex[291]. Dysregulation of the 26S proteasome can heavily affect the PD protein landscape, for it degrades **RGS7**[295], **NR4A2**[296], **GRIN1** (NMDAR)[297], **NFKBIA**[298], SNCA[299], NFKB[300,301], SIRT1[302], HDAC1[303], MAPT[304], MAP3K7[305], PTEN[306,307] and **SOX2**[308]. Reduced activation of the proteosomal complex could therefore increase protein (e.g., SNCA) aggregation, which would affect neuronal functioning.

Mitochondrial function and apoptosis

Mitochondrial dysfunction is associated with both familial and sporadic PD[309]. **BCL2,** located in the nuclear membrane and in the mitochondrial outer membrane**,** is an important anti-apoptotic factor that binds to, inhibits and decreases the expression of the proapoptotic protein BAX[310-313]. BAX inhibition is mediated via the inhibition of **MAPK8** that inhibits the binding of **YWHAZ** and BAX, and in this way increases the translocation of BAX to the mitochondrial membrane[314]. **BCL2** is bound and regulated by multiple proteins in the landscape, i.e., **SATB1** decreases and **NFKBIA** increases **BCL2** expression[315,316]. **MAPK8** also increases **BCL2** expression[317], but inhibits **BCL2** function[318,319]. **BCL2** in turn inhibits **MAPK8**[319], decreases expression of **NFKBIA**[320], **NDRG1**[321] and PTEN[322], increases expression of **SNAP25**[321], and decreases cleavage of **SRPK2**[323]. **BCL2** binds **MAPK8**[324], **CASP3**[325]and PARK2[326], and inhibits apoptotic pathways in that it, in addition to inhibiting BAX, also inhibits **CASP3**[327] and HTRA2[328] and HTRA2 translocation out of mitochondria[329]. In the cytoplasm, HTRA2 binds EIF4G1[330], PARK2 (not shown)[331], PINK1 (not shown)[332] and **CDK5**[332]. **CDK5** in turn inhibits PARK2[176] and increases **TH** expression[7]. SNCA binds to PARK2[333] and, in contrast to **CDK5,** decreases **TH** expression[334,166].

Other proteins in the landscape that affect mitochondrial function are **MRPL15**, **ATP5C1** and **RET**. The 39S ribosomal protein **MRPL15** is located in mitochondria and involved in mitochondrial-specific protein expression. Moreover, **MRPL15** binds to the transcription factor **SOX2** (not shown)[238] and as such may affect DA-neuron-specific expression (see paragraph 'DA neuron signature' in the section 'Transcriptional and translational regulation'). The ATPase **ATP5C1** is part of complex V of the respiratory chain that uses the proton gradient across the mitochondrial membrane to produce ATP from ADP[11]. SNCA may also affect the respiratory chain directly by binding to **ATP5C1**[159]. Lastly, the tyrosine kinase **RET** increases the expression of **TH** and **SLC6A3** (Figure 1a), and ameliorates complex I dysfunction in a PD model[335].

Detailed description of the biological processes depicted in Figure 2. Molecular landscape of interacting proteins, encoded by the mRNAs that are differentially expressed in the striatum of *both* human PD patients and MPTP-treated mice located in the post-synapse of a striatal neuron.

As a result of the dysregulation of the biological processes constituting the molecular landscape of the processes shared in the SN (summarized in figure 1), the release of DA to the striatum is decreased. Due to the lower DA release into the synaptic cleft, affecting protein expression in the striatal post-synapse, the activation of the DA receptors DRD2 and DRD3 is diminished; these receptors are associated with PD[336,337]. When activated, DRD2 (long variant) and DRD3 increase intracellular calcium[338], but they also inhibit the function of the NMDA receptor(NMDAR)[339] and the VDCC[340,341]. The VDCC binds to **ITSN1**[39], a protein involved in actin reorganization and assembly[342,343]. **DCLK1** and **ENC1** are also involved in actin regulation, i.e. **DCLK1** regulates the distribution of actin[344] and **ENC1** is an actin-binding protein[345] that also binds to SNCA[161]. DRD2 also binds to calmodulin (CaM)[346,347] and thereby exerts influence on calcium signaling in the striatal neuron. Namely, CaM binds to the VDCC[142], the NMDAR (not shown)[348], SNCA[349], LRRK2[173], **TGM2**[350], **KCNQ5**[351], **DIRAS2**[352] and **DCLK1**[352], and can thereby affect multiple proteins in the landscape. Furthermore, CaM regulates **KCNQ5**[353] and inhibits calcium flux through the NMDAR into the cell[354,355]. In addition, calcium-bound CaM activates CREB1[356,357] and **CAMK1G**[358], and regulates **TGM2** function (not shown)[350]. **CAMK1G** also activates CREB1[358], and **TGM2** activates ERK1/2[359] and CREB1[360], but also binds to CASP3[361], decreases the expression of **KCNQ5**[362] and increases the expression of LRRK2[362]. **TGM2** is also activated by calcium[363], increases the efflux of calcium out of the cell[364], binds to SNCA[365] and increases its aggregation (not shown)[365,366]. Calcium and CaM therefore affect the activity of ERK1/2 and CREB1 either directly or via the activation of **TGM2** or **CAMK1G**.
Activation of DRD2 by DA also results in the activation of ERK1/2[367] and CREB1[368]. ERK1/2 binds to **CHGB**[369] and the familial proteins SNCA[370] and PARK7[371]. Furthermore, in addition to DRD2 and **TGM2** (see above), **S100A10**[372] and **ITSN1**[373] activate ERK1/2, whereas the nuclear membrane protein **TMEM176B** inhibits ERK1/2 activation[374]. Of note, all these processes converge on CREB1. ERK1/2 activates CREB1[375,376], and CREB1 is activated by CaM, **CAMK1G**, **TGM2** and DRD2 (see above), but also by the NMDAR[356,377] and the (L-type) VDCC (not shown)[356] due to their ability to increase calcium influx, which is necessary for CREB1 activation[378,379]. Thus, CREB1 is regulated by the majority of the proteins in the striatal landscape, either directly or via ERK1/2 activation. Moreover, DA activates both ERK1/2[367] and CREB1 (via the DA receptors)[380,368], suggesting that ERK1/2 and CREB1 activation (via phosphorylation) is reduced in PD or after MPTP treatment due to the absence of DA.

These pathways also play a role in the effect of L-DOPA, the mainstay of treatment in PD. L-DOPA administration activates ERK1/2 in the striatum[381]. DA-induced, CREB1-dependent transcription in the intact striatum in a PD model[382] is further potentiated by NMDAR activation[377]. The secretory granule protein **CHGB** is one of the proteins of which the expression is regulated by CREB1, i.e. CREB1 binds to the CRE element of the **CHGB** gene promoter[383]. Furthermore, calcium decreases the expression of **CHGB**[384] and **CHGB** binds to PARK2[263]. In addition to ERK1/2 and CREB1, L-DOPA also activates DRD2[385 (DB01235#target-831)], DRD3[385 (DB01235#target-683)]

and NMDAR[385 (DB01235#target-683),386], and increases the expression of DRD3[386], CASP3[387] and **S100A10**[388]. In a PD rat model, **S100A10** is involved in L-DOPA-induced abnormal involuntary movements[389]. The activation of striatal ERK1/2 by L-DOPA also appears involved in L-DOPA-induced dyskinesias[389], but not the L-DOPA induced CREB1 activation[390,391,381]. These processes could therefore not only give insights into the PD-related disease mechanisms in the striatum, but also in the beneficial, and adverse, effects of pharmacological treatment.

CREB1 and ERK1/2 are also known for their role in epilepsy. Brain areas prone to epileptic seizures show an increased activation of CREB1 and ERK1/2[382], and an up regulation of **CHGB**[392], CREB1[392], **ENC1**[356] and **NPTX2**[392]. **NPTX2** is thought to play a role in long-term plasticity [392] and increases apoptosis[11]. Further, **KCNQ5**[393], the NMDAR[394] and the VDCC[395] are associated with epileptic seizures. Therefore, the landscape cannot only give insight in treatment outcome, but can also explain the associations seen in functional studies with PD, in this respect with epilepsy[396].

REFERENCES

1. Reguzzoni M, Cosentino M, Rasini E, Marino F, Ferrari M, Bombelli R, Congiu T, Protasoni M, Quacci D, Lecchini S, Raspanti M, Frigo G (2002) Ultrastructural localization of tyrosine hydroxylase in human peripheral blood mononuclear cells: effect of stimulation with phytohaemagglutinin. Cell Tissue Res 310 (3):297-304. doi:10.1007/s00441-002-0617-9
2. Kato T, Abe Y, Sotoyama H, Kakita A, Kominami R, Hirokawa S, Ozaki M, Takahashi H, Nawa H (2011) Transient exposure of neonatal mice to neuregulin-1 results in hyperdopaminergic states in adulthood: implication in neurodevelopmental hypothesis for schizophrenia. Mol Psychiatry 16 (3):307-320. doi:10.1038/mp.2010.10
3. Mijatovic J, Airavaara M, Planken A, Auvinen P, Raasmaja A, Piepponen TP, Costantini F, Ahtee L, Saarma M (2007) Constitutive Ret activity in knock-in multiple endocrine neoplasia type B mice induces profound elevation of brain dopamine concentration via enhanced synthesis and increases the number of TH-positive cells in the substantia nigra. J Neurosci 27 (18):4799-4809. doi:10.1523/jneurosci.5647-06.2007
4. Jones SR, Gainetdinov RR, Jaber M, Giros B, Wightman RM, Caron MG (1998) Profound neuronal plasticity in response to inactivation of the dopamine transporter. Proc Natl Acad Sci U S A 95 (7):4029-4034
5. Obsilova V, Nedbalkova E, Silhan J, Boura E, Herman P, Vecer J, Sulc M, Teisinger J, Dyda F, Obsil T (2008) The 14-3-3 protein affects the conformation of the regulatory domain of human tyrosine hydroxylase. Biochemistry 47 (6):1768-1777. doi:10.1021/bi7019468
6. Wang J, Lou H, Pedersen CJ, Smith AD, Perez RG (2009) 14-3-3zeta contributes to tyrosine hydroxylase activity in MN9D cells: localization of dopamine regulatory proteins to mitochondria. J Biol Chem 284 (21):14011-14019. doi:10.1074/jbc.M901310200
7. Moy LY, Tsai LH (2004) Cyclin-dependent kinase 5 phosphorylates serine 31 of tyrosine hydroxylase and regulates its stability. J Biol Chem 279 (52):54487-54493. doi:10.1074/jbc.M406636200
8. Wachtel SR, Bencsics C, Kang UJ (1997) Role of aromatic L-amino acid decarboxylase for dopamine replacement by genetically modified fibroblasts in a rat model of Parkinson's disease. J Neurochem 69 (5):2055-2063
9. Chang JW, Lee WY, Milstien S, Kang UJ (2002) A site-specific mutation of tyrosine hydroxylase reduces feedback inhibition by dopamine in genetically modified cells grafted in parkinsonian rats. Journal of neurochemistry 83 (1):141-149
10. Suzuki A, Hirata M, Kamimura K, Maniwa R, Yamanaka T, Mizuno K, Kishikawa M, Hirose H, Amano Y, Izumi N, Miwa Y, Ohno S (2004) aPKC acts upstream of PAR-1b in both the establishment and maintenance of mammalian epithelial polarity. Current biology : CB 14 (16):1425-1435. doi:10.1016/j.cub.2004.08.021
11. Activities at the Universal Protein Resource (UniProt) (2014). Nucleic acids research 42 (Database issue):D191-198. doi:10.1093/nar/gkt1140
12. Meek SE, Lane WS, Piwnica-Worms H (2004) Comprehensive proteomic analysis of interphase and mitotic 14-3-3-binding proteins. J Biol Chem 279 (31):32046-32054. doi:10.1074/jbc.M403044200
13. Gillbro JM, Marles LK, Hibberts NA, Schallreuter KU (2004) Autocrine catecholamine biosynthesis and the beta-adrenoceptor signal promote pigmentation in human epidermal melanocytes. The Journal of investigative dermatology 123 (2):346-353. doi:10.1111/j.0022-202X.2004.23210.x
14. Misura KM, Gonzalez LC, Jr., May AP, Scheller RH, Weis WI (2001) Crystal structure and biophysical properties of a complex between the N-terminal SNARE region of SNAP25 and syntaxin 1a. J Biol Chem 276 (44):41301-41309. doi:10.1074/jbc.M106853200
15. Gorini G, Ponomareva O, Shores KS, Person MD, Harris RA, Mayfield RD (2010) Dynamin-1 co-associates with native mouse brain BKCa channels: proteomics analysis of synaptic protein complexes. FEBS Lett 584 (5):845-851. doi:10.1016/j.febslet.2009.12.061

16. Bhaskar K, Shareef MM, Sharma VM, Shetty AP, Ramamohan Y, Pant HC, Raju TR, Shetty KT (2004) Co-purification and localization of Munc18-1 (p67) and Cdk5 with neuronal cytoskeletal proteins. Neurochem Int 44 (1):35-44
17. Yan Q, Sun W, McNew JA, Vida TA, Bean AJ (2004) Ca2+ and N-ethylmaleimide-sensitive factor differentially regulate disassembly of SNARE complexes on early endosomes. J Biol Chem 279 (18):18270-18276. doi:10.1074/jbc.M400093200
18. Angrand PO, Segura I, Volkel P, Ghidelli S, Terry R, Brajenovic M, Vintersten K, Klein R, Superti-Furga G, Drewes G, Kuster B, Bouwmeester T, Acker-Palmer A (2006) Transgenic mouse proteomics identifies new 14-3-3-associated proteins involved in cytoskeletal rearrangements and cell signaling. Mol Cell Proteomics 5 (12):2211-2227. doi:10.1074/mcp.M600147-MCP200
19. Klein JB, Barati MT, Wu R, Gozal D, Sachleben LR, Jr., Kausar H, Trent JO, Gozal E, Rane MJ (2005) Akt-mediated valosin-containing protein 97 phosphorylation regulates its association with ubiquitinated proteins. J Biol Chem 280 (36):31870-31881. doi:10.1074/jbc.M501802200
20. Rego AC, de Almeida LP (2005) Molecular targets and therapeutic strategies in Huntington's disease. Curr Drug Targets CNS Neurol Disord 4 (4):361-381
21. Zhu W, Wang D, Zheng J, An Y, Wang Q, Zhang W, Jin L, Gao H, Lin L (2008) Effect of (R)-salsolinol and N-methyl-(R)-salsolinol on the balance impairment between dopamine and acetylcholine in rat brain: involvement in pathogenesis of Parkinson disease. Clinical chemistry 54 (4):705-712. doi:10.1373/clinchem.2007.097725
22. Patel JC, Rossignol E, Rice ME, Machold RP (2012) Opposing regulation of dopaminergic activity and exploratory motor behavior by forebrain and brainstem cholinergic circuits. Nature communications 3:1172. doi:10.1038/ncomms2144
23. Shimada H, Hirano S, Shinotoh H, Aotsuka A, Sato K, Tanaka N, Ota T, Asahina M, Fukushi K, Kuwabara S, Hattori T, Suhara T, Irie T (2009) Mapping of brain acetylcholinesterase alterations in Lewy body disease by PET. Neurology 73 (4):273-278. doi:10.1212/WNL.0b013e3181ab2b58
24. Zang LY, Misra HP (2003) Inactivation of acetylcholinesterase by 1-methyl-4-phenyl-1,2,3,6-tetrahydropyridine hydrochloride. Mol Cell Biochem 254 (1-2):131-136
25. Zhang X, Lu L, Liu S, Ye W, Wu J, Zhang X (2013) Acetylcholinesterase deficiency decreases apoptosis in dopaminergic neurons in the neurotoxin model of Parkinson's disease. The international journal of biochemistry & cell biology 45 (2):265-272. doi:10.1016/j.biocel.2012.11.015
26. Figlewicz DP (1999) Endocrine regulation of neurotransmitter transporters. Epilepsy Res 37 (3):203-210
27. Yorgason JT, Jones SR, Espana RA (2011) Low and high affinity dopamine transporter inhibitors block dopamine uptake within 5 sec of intravenous injection. Neuroscience 182:125-132. doi:10.1016/j.neuroscience.2011.03.017
28. Jiang H, Jiang Q, Feng J (2004) Parkin increases dopamine uptake by enhancing the cell surface expression of dopamine transporter. J Biol Chem 279 (52):54380-54386. doi:10.1074/jbc.M409282200
29. Lee FJ, Liu F, Pristupa ZB, Niznik HB (2001) Direct binding and functional coupling of alpha-synuclein to the dopamine transporters accelerate dopamine-induced apoptosis. FASEB J 15 (6):916-926
30. Lin Z, Canales JJ, Bjorgvinsson T, Thomsen M, Qu H, Liu QR, Torres GE, Caine SB (2011) Monoamine transporters: vulnerable and vital doorkeepers. Prog Mol Biol Transl Sci 98:1-46. doi:10.1016/b978-0-12-385506-0.00001-6
31. Carter CJ (2007) eIF2B and oligodendrocyte survival: where nature and nurture meet in bipolar disorder and schizophrenia? Schizophr Bull 33 (6):1343-1353. doi:10.1093/schbul/sbm007
32. Shelton SB, Johnson GV (2004) Cyclin-dependent kinase-5 in neurodegeneration. J Neurochem 88 (6):1313-1326

33. Cyr M, Beaulieu JM, Laakso A, Sotnikova TD, Yao WD, Bohn LM, Gainetdinov RR, Caron MG (2003) Sustained elevation of extracellular dopamine causes motor dysfunction and selective degeneration of striatal GABAergic neurons. Proc Natl Acad Sci U S A 100 (19):11035-11040. doi:10.1073/pnas.1831768100
34. Bolan EA, Kivell B, Jaligam V, Oz M, Jayanthi LD, Han Y, Sen N, Urizar E, Gomes I, Devi LA, Ramamoorthy S, Javitch JA, Zapata A, Shippenberg TS (2007) D2 receptors regulate dopamine transporter function via an extracellular signal-regulated kinases 1 and 2-dependent and phosphoinositide 3 kinase-independent mechanism. Mol Pharmacol 71 (5):1222-1232. doi:10.1124/mol.106.027763
35. Lee FJ, Pei L, Moszczynska A, Vukusic B, Fletcher PJ, Liu F (2007) Dopamine transporter cell surface localization facilitated by a direct interaction with the dopamine D2 receptor. EMBO J 26 (8):2127-2136. doi:10.1038/sj.emboj.7601656
36. Blakely RD, DeFelice LJ (2007) All aglow about presynaptic receptor regulation of neurotransmitter transporters. Mol Pharmacol 71 (5):1206-1208. doi:10.1124/mol.107.035493
37. Torres GE (2006) The dopamine transporter proteome. J Neurochem 97 Suppl 1:3-10. doi:10.1111/j.1471-4159.2006.03719.x
38. Rettig J, Sheng ZH, Kim DK, Hodson CD, Snutch TP, Catterall WA (1996) Isoform-specific interaction of the alpha1A subunits of brain Ca2+ channels with the presynaptic proteins syntaxin and SNAP-25. Proc Natl Acad Sci U S A 93 (14):7363-7368
39. Muller CS, Haupt A, Bildl W, Schindler J, Knaus HG, Meissner M, Rammner B, Striessnig J, Flockerzi V, Fakler B, Schulte U (2010) Quantitative proteomics of the Cav2 channel nano-environments in the mammalian brain. Proc Natl Acad Sci U S A 107 (34):14950-14957. doi:10.1073/pnas.1005940107
40. Lopez-Aranda MF, Acevedo MJ, Gutierrez A, Koulen P, Khan ZU (2007) Role of a Galphai2 protein splice variant in the formation of an intracellular dopamine D2 receptor pool. J Cell Sci 120 (Pt 13):2171-2178. doi:10.1242/jcs.005611
41. Jaen C, Doupnik CA (2006) RGS3 and RGS4 differentially associate with G protein-coupled receptor-Kir3 channel signaling complexes revealing two modes of RGS modulation. Precoupling and collision coupling. J Biol Chem 281 (45):34549-34560. doi:10.1074/jbc.M603177200
42. Druey KM, Sullivan BM, Brown D, Fischer ER, Watson N, Blumer KJ, Gerfen CR, Scheschonka A, Kehrl JH (1998) Expression of GTPase-deficient Gialpha2 results in translocation of cytoplasmic RGS4 to the plasma membrane. J Biol Chem 273 (29):18405-18410
43. Heximer SP, Watson N, Linder ME, Blumer KJ, Hepler JR (1997) RGS2/G0S8 is a selective inhibitor of Gqalpha function. Proc Natl Acad Sci U S A 94 (26):14389-14393
44. Roy AA, Lemberg KE, Chidiac P (2003) Recruitment of RGS2 and RGS4 to the plasma membrane by G proteins and receptors reflects functional interactions. Mol Pharmacol 64 (3):587-593. doi:10.1124/mol.64.3.587
45. Walf-Vorderwulbecke V, de Boer J, Horton SJ, van Amerongen R, Proost N, Berns A, Williams O (2012) Frat2 mediates the oncogenic activation of Rac by MLL fusions. Blood 120 (24):4819-4828. doi:10.1182/blood-2012-05-432534
46. Liu Z, Adams HC, 3rd, Whitehead IP (2009) The rho-specific guanine nucleotide exchange factor Dbs regulates breast cancer cell migration. The Journal of biological chemistry 284 (23):15771-15780. doi:10.1074/jbc.M901853200
47. Feng H, Hu B, Jarzynka MJ, Li Y, Keezer S, Johns TG, Tang CK, Hamilton RL, Vuori K, Nishikawa R, Sarkaria JN, Fenton T, Cheng T, Furnari FB, Cavenee WK, Cheng SY (2012) Phosphorylation of dedicator of cytokinesis 1 (Dock180) at tyrosine residue Y722 by Src family kinases mediates EGFRvIII-driven glioblastoma tumorigenesis. Proc Natl Acad Sci U S A 109 (8):3018-3023. doi:10.1073/pnas.1121457109
48. Wang Y, Zhao L, Smas C, Sul HS (2010) Pref-1 interacts with fibronectin to inhibit adipocyte differentiation. Mol Cell Biol 30 (14):3480-3492. doi:10.1128/mcb.00057-10

49. Chiariello M, Visconti R, Carlomagno F, Melillo RM, Bucci C, de Franciscis V, Fox GM, Jing S, Coso OA, Gutkind JS, Fusco A, Santoro M (1998) Signalling of the Ret receptor tyrosine kinase through the c-Jun NH2-terminal protein kinases (JNKS): evidence for a divergence of the ERKs and JNKs pathways induced by Ret. Oncogene 16 (19):2435-2445. doi:10.1038/sj.onc.1201778
50. Fukuda T, Kiuchi K, Takahashi M (2002) Novel mechanism of regulation of Rac activity and lamellipodia formation by RET tyrosine kinase. J Biol Chem 277 (21):19114-19121. doi:10.1074/jbc.M200643200
51. Hama K, Aoki J, Fukaya M, Kishi Y, Sakai T, Suzuki R, Ohta H, Yamori T, Watanabe M, Chun J, Arai H (2004) Lysophosphatidic acid and autotaxin stimulate cell motility of neoplastic and non-neoplastic cells through LPA1. J Biol Chem 279 (17):17634-17639. doi:10.1074/jbc.M313927200
52. Kaibuchi K, Kuroda S, Amano M (1999) Regulation of the cytoskeleton and cell adhesion by the Rho family GTPases in mammalian cells. Annu Rev Biochem 68:459-486. doi:10.1146/annurev.biochem.68.1.459
53. Alexander K, Yang HS, Hinds PW (2004) Cellular senescence requires CDK5 repression of Rac1 activity. Mol Cell Biol 24 (7):2808-2819
54. Mukherjee J, DeSouza LV, Micallef J, Karim Z, Croul S, Siu KW, Guha A (2009) Loss of collapsin response mediator Protein1, as detected by iTRAQ analysis, promotes invasion of human gliomas expressing mutant EGFRvIII. Cancer Res 69 (22):8545-8554. doi:10.1158/0008-5472.can-09-1778
55. Papin J, Subramaniam S (2004) Bioinformatics and cellular signaling. Curr Opin Biotechnol 15 (1):78-81. doi:10.1016/j.copbio.2004.01.003
56. Bandyopadhyay S, Chiang CY, Srivastava J, Gersten M, White S, Bell R, Kurschner C, Martin C, Smoot M, Sahasrabudhe S, Barber DL, Chanda SK, Ideker T (2010) A human MAP kinase interactome. Nat Methods 7 (10):801-805
57. Shimizu K, Kawabe H, Minami S, Honda T, Takaishi K, Shirataki H, Takai Y (1996) SMAP, an Smg GDS-associating protein having arm repeats and phosphorylated by Src tyrosine kinase. J Biol Chem 271 (43):27013-27017
58. Chuang TH, Xu X, Quilliam LA, Bokoch GM (1994) SmgGDS stabilizes nucleotide-bound and -free forms of the Rac1 GTP-binding protein and stimulates GTP/GDP exchange through a substituted enzyme mechanism. The Biochemical journal 303 (Pt 3):761-767
59. Woods A, Wang G, Dupuis H, Shao Z, Beier F (2007) Rac1 signaling stimulates N-cadherin expression, mesenchymal condensation, and chondrogenesis. J Biol Chem 282 (32):23500-23508. doi:10.1074/jbc.M700680200
60. Saoncella S, Calautti E, Neveu W, Goetinck PF (2004) Syndecan-4 regulates ATF-2 transcriptional activity in a Rac1-dependent manner. J Biol Chem 279 (45):47172-47176. doi:10.1074/jbc.C400299200
61. Sanna MG, da Silva Correia J, Ducrey O, Lee J, Nomoto K, Schrantz N, Deveraux QL, Ulevitch RJ (2002) IAP suppression of apoptosis involves distinct mechanisms: the TAK1/JNK1 signaling cascade and caspase inhibition. Mol Cell Biol 22 (6):1754-1766
62. Enomoto A, Suzuki N, Morita A, Ito M, Liu CQ, Matsumoto Y, Yoshioka K, Shiba T, Hosoi Y (2003) Caspase-mediated cleavage of JNK during stress-induced apoptosis. Biochem Biophys Res Commun 306 (4):837-842
63. Kwak D, Choi S, Jeong H, Jang JH, Lee Y, Jeon H, Lee MN, Noh J, Cho K, Yoo JS, Hwang D, Suh PG, Ryu SH (2012) Osmotic stress regulates mammalian target of rapamycin (mTOR) complex 1 via c-Jun N-terminal Kinase (JNK)-mediated Raptor protein phosphorylation. J Biol Chem 287 (22):18398-18407. doi:10.1074/jbc.M111.326538
64. Nguyen Q, Lee CM, Le A, Reddy EP (2005) JLP associates with kinesin light chain 1 through a novel leucine zipper-like domain. J Biol Chem 280 (34):30185-30191. doi:10.1074/jbc.M505499200

65. Salehi AH, Xanthoudakis S, Barker PA (2002) NRAGE, a p75 neurotrophin receptor-interacting protein, induces caspase activation and cell death through a JNK-dependent mitochondrial pathway. J Biol Chem 277 (50):48043-48050. doi:10.1074/jbc.M205324200
66. Yamauchi J, Kawano T, Nagao M, Kaziro Y, Itoh H (2000) G(i)-dependent activation of c-Jun N-terminal kinase in human embryonal kidney 293 cells. J Biol Chem 275 (11):7633-7640
67. Villanueva A, Lozano J, Morales A, Lin X, Deng X, Hengartner MO, Kolesnick RN (2001) jkk-1 and mek-1 regulate body movement coordination and response to heavy metals through jnk-1 in Caenorhabditis elegans. EMBO J 20 (18):5114-5128. doi:10.1093/emboj/20.18.5114
68. Contos JJ, Ishii I, Fukushima N, Kingsbury MA, Ye X, Kawamura S, Brown JH, Chun J (2002) Characterization of lpa(2) (Edg4) and lpa(1)/lpa(2) (Edg2/Edg4) lysophosphatidic acid receptor knockout mice: signaling deficits without obvious phenotypic abnormality attributable to lpa(2). Mol Cell Biol 22 (19):6921-6929
69. Luo Y, Umegaki H, Wang X, Abe R, Roth GS (1998) Dopamine induces apoptosis through an oxidation-involved SAPK/JNK activation pathway. J Biol Chem 273 (6):3756-3764
70. Park HS, Kim MS, Huh SH, Park J, Chung J, Kang SS, Choi EJ (2002) Akt (protein kinase B) negatively regulates SEK1 by means of protein phosphorylation. J Biol Chem 277 (4):2573-2578. doi:10.1074/jbc.M110299200
71. Kendall SE, Battelli C, Irwin S, Mitchell JG, Glackin CA, Verdi JM (2005) NRAGE mediates p38 activation and neural progenitor apoptosis via the bone morphogenetic protein signaling cascade. Mol Cell Biol 25 (17):7711-7724. doi:10.1128/mcb.25.17.7711-7724.2005
72. Ewing RM, Chu P, Elisma F, Li H, Taylor P, Climie S, McBroom-Cerajewski L, Robinson MD, O'Connor L, Li M, Taylor R, Dharsee M, Ho Y, Heilbut A, Moore L, Zhang S, Ornatsky O, Bukhman YV, Ethier M, Sheng Y, Vasilescu J, Abu-Farha M, Lambert JP, Duewel HS, Stewart, II, Kuehl B, Hogue K, Colwill K, Gladwish K, Muskat B, Kinach R, Adams SL, Moran MF, Morin GB, Topaloglou T, Figeys D (2007) Large-scale mapping of human protein-protein interactions by mass spectrometry. Mol Syst Biol 3:89. doi:10.1038/msb4100134
73. Garcia-Fernandez LF, Losada A, Alcaide V, Alvarez AM, Cuadrado A, Gonzalez L, Nakayama K, Nakayama KI, Fernandez-Sousa JM, Munoz A, Sanchez-Puelles JM (2002) Aplidin induces the mitochondrial apoptotic pathway via oxidative stress-mediated JNK and p38 activation and protein kinase C delta. Oncogene 21 (49):7533-7544. doi:10.1038/sj.onc.1205972
74. Wu SS, Chen LG, Lin RJ, Lin SY, Lo YE, Liang YC (2013) Cytotoxicity of (-)-vitisin B in human leukemia cells. Drug Chem Toxicol 36 (3):313-319. doi:10.3109/01480545.2012.720990
75. Abdelli S, Bonny C (2012) JNK3 maintains expression of the insulin receptor substrate 2 (IRS2) in insulin-secreting cells: functional consequences for insulin signaling. PLoS One 7 (5):e35997. doi:10.1371/journal.pone.0035997
76. Yang R, Wilcox DM, Haasch DL, Jung PM, Nguyen PT, Voorbach MJ, Doktor S, Brodjian S, Bush EN, Lin E, Jacobson PB, Collins CA, Landschulz KT, Trevillyan JM, Rondinone CM, Surowy TK (2007) Liver-specific knockdown of JNK1 up-regulates proliferator-activated receptor gamma coactivator 1 beta and increases plasma triglyceride despite reduced glucose and insulin levels in diet-induced obese mice. J Biol Chem 282 (31):22765-22774. doi:10.1074/jbc.M700790200
77. Zama T, Aoki R, Kamimoto T, Inoue K, Ikeda Y, Hagiwara M (2002) A novel dual specificity phosphatase SKRP1 interacts with the MAPK kinase MKK7 and inactivates the JNK MAPK pathway. Implication for the precise regulation of the particular MAPK pathway. The Journal of biological chemistry 277 (26):23909-23918. doi:10.1074/jbc.M200837200
78. Li MG, Katsura K, Nomiyama H, Komaki K, Ninomiya-Tsuji J, Matsumoto K, Kobayashi T, Tamura S (2003) Regulation of the interleukin-1-induced signaling pathways by a novel member of the protein phosphatase 2C family (PP2Cepsilon). J Biol Chem 278 (14):12013-12021. doi:10.1074/jbc.M211474200
79. Tibbles LA, Woodgett JR (1999) The stress-activated protein kinase pathways. Cell Mol Life Sci 55 (10):1230-1254

80. Powell DW, Rane MJ, Chen Q, Singh S, McLeish KR (2002) Identification of 14-3-3zeta as a protein kinase B/Akt substrate. J Biol Chem 277 (24):21639-21642. doi:10.1074/jbc.M203167200
81. Dickey CA, Koren J, Zhang YJ, Xu YF, Jinwal UK, Birnbaum MJ, Monks B, Sun M, Cheng JQ, Patterson C, Bailey RM, Dunmore J, Soresh S, Leon C, Morgan D, Petrucelli L (2008) Akt and CHIP coregulate tau degradation through coordinated interactions. Proceedings of the National Academy of Sciences of the United States of America 105 (9):3622-3627. doi:10.1073/pnas.0709180105
82. Barry EF, Felquer FA, Powell JA, Biggs L, Stomski FC, Urbani A, Ramshaw H, Hoffmann P, Wilce MC, Grimbaldeston MA, Lopez AF, Guthridge MA (2009) 14-3-3:Shc scaffolds integrate phosphoserine and phosphotyrosine signaling to regulate phosphatidylinositol 3-kinase activation and cell survival. J Biol Chem 284 (18):12080-12090. doi:10.1074/jbc.M807637200
83. Woulfe D, Jiang H, Morgans A, Monks R, Birnbaum M, Brass LF (2004) Defects in secretion, aggregation, and thrombus formation in platelets from mice lacking Akt2. J Clin Invest 113 (3):441-450. doi:10.1172/jci20267
84. Mahajan K, Coppola D, Chen YA, Zhu W, Lawrence HR, Lawrence NJ, Mahajan NP (2012) Ack1 tyrosine kinase activation correlates with pancreatic cancer progression. The American journal of pathology 180 (4):1386-1393. doi:10.1016/j.ajpath.2011.12.028
85. Ponnusamy MP, Lakshmanan I, Jain M, Das S, Chakraborty S, Dey P, Batra SK (2010) MUC4 mucin-induced epithelial to mesenchymal transition: a novel mechanism for metastasis of human ovarian cancer cells. Oncogene 29 (42):5741-5754. doi:10.1038/onc.2010.309
86. Aguilar B, Choi I, Choi D, Chung HK, Lee S, Yoo J, Lee YS, Maeng YS, Lee HN, Park E, Kim KE, Kim NY, Baik JM, Jung JU, Koh CJ, Hong YK (2012) Lymphatic reprogramming by Kaposi sarcoma herpes virus promotes the oncogenic activity of the virus-encoded G-protein-coupled receptor. Cancer Res 72 (22):5833-5842. doi:10.1158/0008-5472.can-12-1229
87. Chen L, Qanie D, Jafari A, Taipaleenmaki H, Jensen CH, Saamanen AM, Sanz ML, Laborda J, Abdallah BM, Kassem M (2011) Delta-like 1/fetal antigen-1 (Dlk1/FA1) is a novel regulator of chondrogenic cell differentiation via inhibition of the Akt kinase-dependent pathway. J Biol Chem 286 (37):32140-32149. doi:10.1074/jbc.M111.230110
88. Negishi M, Katoh H (2002) Rho family GTPases as key regulators for neuronal network formation. Journal of biochemistry 132 (2):157-166
89. Wang J, Huo K, Ma L, Tang L, Li D, Huang X, Yuan Y, Li C, Wang W, Guan W, Chen H, Jin C, Wei J, Zhang W, Yang Y, Liu Q, Zhou Y, Zhang C, Wu Z, Xu W, Zhang Y, Liu T, Yu D, Zhang Y, Chen L, Zhu D, Zhong X, Kang L, Gan X, Yu X, Ma Q, Yan J, Zhou L, Liu Z, Zhu Y, Zhou T, He F, Yang X (2011) Toward an understanding of the protein interaction network of the human liver. Molecular systems biology 7:536. doi:10.1038/msb.2011.67
90. Bouwmeester T, Bauch A, Ruffner H, Angrand PO, Bergamini G, Croughton K, Cruciat C, Eberhard D, Gagneur J, Ghidelli S, Hopf C, Huhse B, Mangano R, Michon AM, Schirle M, Schlegl J, Schwab M, Stein MA, Bauer A, Casari G, Drewes G, Gavin AC, Jackson DB, Joberty G, Neubauer G, Rick J, Kuster B, Superti-Furga G (2004) A physical and functional map of the human TNF-alpha/NF-kappa B signal transduction pathway. Nat Cell Biol 6 (2):97-105. doi:10.1038/ncb1086
91. Tu LC, Yan X, Hood L, Lin B (2007) Proteomics analysis of the interactome of N-myc downstream regulated gene 1 and its interactions with the androgen response program in prostate cancer cells. Molecular & cellular proteomics : MCP 6 (4):575-588. doi:10.1074/mcp.M600249-MCP200
92. Taddei ML, Chiarugi P, Cirri P, Buricchi F, Fiaschi T, Giannoni E, Talini D, Cozzi G, Formigli L, Raugei G, Ramponi G (2002) Beta-catenin interacts with low-molecular-weight protein tyrosine phosphatase leading to cadherin-mediated cell-cell adhesion increase. Cancer research 62 (22):6489-6499
93. Wang YJ, Gregory RB, Barritt GJ (2000) Regulation of F-actin and endoplasmic reticulum organization by the trimeric G-protein Gi2 in rat hepatocytes. Implication for the activation of

store-operated Ca2+ inflow. J Biol Chem 275 (29):22229-22237. doi:10.1074/jbc.M001563200
94. Lenz SE, Braunewell KH, Weise C, Nedlina-Chittka A, Gundelfinger ED (1996) The neuronal EF-hand Ca(2+)-binding protein VILIP: interaction with cell membrane and actin-based cytoskeleton. Biochem Biophys Res Commun 225 (3):1078-1083. doi:10.1006/bbrc.1996.1298
95. Husi H, Ward MA, Choudhary JS, Blackstock WP, Grant SG (2000) Proteomic analysis of NMDA receptor-adhesion protein signaling complexes. Nat Neurosci 3 (7):661-669. doi:10.1038/76615
96. Thomas SM, Brugge JS (1997) Cellular functions regulated by Src family kinases. Annu Rev Cell Dev Biol 13:513-609. doi:10.1146/annurev.cellbio.13.1.513
97. Linseman DA, Heidenreich KA, Fisher SK (2001) Stimulation of M3 muscarinic receptors induces phosphorylation of the Cdc42 effector activated Cdc42Hs-associated kinase-1 via a Fyn tyrosine kinase signaling pathway. The Journal of biological chemistry 276 (8):5622-5628. doi:10.1074/jbc.M006812200
98. Jimenez B, Volpert OV, Crawford SE, Febbraio M, Silverstein RL, Bouck N (2000) Signals leading to apoptosis-dependent inhibition of neovascularization by thrombospondin-1. Nat Med 6 (1):41-48. doi:10.1038/71517
99. Ricci JE, Maulon L, Luciano F, Guerin S, Livolsi A, Mari B, Breittmayer JP, Peyron JF, Auberger P (1999) Cleavage and relocation of the tyrosine kinase P59FYN during Fas-mediated apoptosis in T lymphocytes. Oncogene 18 (27):3963-3969. doi:10.1038/sj.onc.1202782
100. Luciano F, Ricci JE, Auberger P (2001) Cleavage of Fyn and Lyn in their N-terminal unique regions during induction of apoptosis: a new mechanism for Src kinase regulation. Oncogene 20 (36):4935-4941. doi:10.1038/sj.onc.1204661
101. Jurd R, Tretter V, Walker J, Brandon NJ, Moss SJ (2010) Fyn kinase contributes to tyrosine phosphorylation of the GABA(A) receptor gamma2 subunit. Mol Cell Neurosci 44 (2):129-134. doi:10.1016/j.mcn.2010.03.002
102. Reynolds CH, Garwood CJ, Wray S, Price C, Kellie S, Perera T, Zvelebil M, Yang A, Sheppard PW, Varndell IM, Hanger DP, Anderton BH (2008) Phosphorylation regulates tau interactions with Src homology 3 domains of phosphatidylinositol 3-kinase, phospholipase Cgamma1, Grb2, and Src family kinases. J Biol Chem 283 (26):18177-18186. doi:10.1074/jbc.M709715200
103. Lee G, Thangavel R, Sharma VM, Litersky JM, Bhaskar K, Fang SM, Do LH, Andreadis A, Van Hoesen G, Ksiezak-Reding H (2004) Phosphorylation of tau by fyn: implications for Alzheimer's disease. J Neurosci 24 (9):2304-2312. doi:10.1523/jneurosci.4162-03.2004
104. Yang K, Belrose J, Trepanier CH, Lei G, Jackson MF, MacDonald JF (2011) Fyn, a potential target for Alzheimer's disease. J Alzheimers Dis 27 (2):243-252. doi:10.3233/jad-2011-110353
105. Tobin JE, Latourelle JC, Lew MF, Klein C, Suchowersky O, Shill HA, Golbe LI, Mark MH, Growdon JH, Wooten GF, Racette BA, Perlmutter JS, Watts R, Guttman M, Baker KB, Goldwurm S, Pezzoli G, Singer C, Saint-Hilaire MH, Hendricks AE, Williamson S, Nagle MW, Wilk JB, Massood T, Laramie JM, DeStefano AL, Litvan I, Nicholson G, Corbett A, Isaacson S, Burn DJ, Chinnery PF, Pramstaller PP, Sherman S, Al-hinti J, Drasby E, Nance M, Moller AT, Ostergaard K, Roxburgh R, Snow B, Slevin JT, Cambi F, Gusella JF, Myers RH (2008) Haplotypes and gene expression implicate the MAPT region for Parkinson disease: the GenePD Study. Neurology 71 (1):28-34. doi:10.1212/01.wnl.0000304051.01650.23
106. Wider C, Vilarino-Guell C, Jasinska-Myga B, Heckman MG, Soto-Ortolaza AI, Cobb SA, Aasly JO, Gibson JM, Lynch T, Uitti RJ, Wszolek ZK, Farrer MJ, Ross OA (2010) Association of the MAPT locus with Parkinson's disease. European journal of neurology : the official journal of the European Federation of Neurological Societies 17 (3):483-486. doi:10.1111/j.1468-1331.2009.02847.x
107. Elbaz A, Ross OA, Ioannidis JP, Soto-Ortolaza AI, Moisan F, Aasly J, Annesi G, Bozi M, Brighina L, Chartier-Harlin MC, Destee A, Ferrarese C, Ferraris A, Gibson JM, Gispert S, Hadjigeorgiou GM, Jasinska-Myga B, Klein C, Kruger R, Lambert JC, Lohmann K, van de Loo S, Loriot MA,

Lynch T, Mellick GD, Mutez E, Nilsson C, Opala G, Puschmann A, Quattrone A, Sharma M, Silburn PA, Stefanis L, Uitti RJ, Valente EM, Vilarino-Guell C, Wirdefeldt K, Wszolek ZK, Xiromerisiou G, Maraganore DM, Farrer MJ (2011) Independent and joint effects of the MAPT and SNCA genes in Parkinson disease. Ann Neurol 69 (5):778-792. doi:10.1002/ana.22321

108. Trotta L, Guella I, Solda G, Sironi F, Tesei S, Canesi M, Pezzoli G, Goldwurm S, Duga S, Asselta R (2012) SNCA and MAPT genes: Independent and joint effects in Parkinson disease in the Italian population. Parkinsonism Relat Disord 18 (3):257-262. doi:10.1016/j.parkreldis.2011.10.014
109. Gan-Or Z, Bar-Shira A, Mirelman A, Gurevich T, Giladi N, Orr-Urtreger A (2012) The age at motor symptoms onset in LRRK2-associated Parkinson's disease is affected by a variation in the MAPT locus: a possible interaction. Journal of molecular neuroscience : MN 46 (3):541-544. doi:10.1007/s12031-011-9641-0
110. Esteves AR, Gozes I, Cardoso SM (2014) The rescue of microtubule-dependent traffic recovers mitochondrial function in Parkinson's disease. Biochimica et biophysica acta 1842 (1):7-21. doi:10.1016/j.bbadis.2013.10.003
111. Arduino DM, Esteves AR, Cardoso SM (2013) Mitochondria drive autophagy pathology via microtubule disassembly: a new hypothesis for Parkinson disease. Autophagy 9 (1):112-114. doi:10.4161/auto.22443
112. Gu GJ, Wu D, Lund H, Sunnemark D, Kvist AJ, Milner R, Eckersley S, Nilsson LN, Agerman K, Landegren U, Kamali-Moghaddam M (2013) Elevated MARK2-dependent phosphorylation of Tau in Alzheimer's disease. Journal of Alzheimer's disease : JAD 33 (3):699-713. doi:10.3233/jad-2012-121357
113. Utton MA, Noble WJ, Hill JE, Anderton BH, Hanger DP (2005) Molecular motors implicated in the axonal transport of tau and alpha-synuclein. J Cell Sci 118 (Pt 20):4645-4654. doi:10.1242/jcs.02558
114. Sengupta A, Kabat J, Novak M, Wu Q, Grundke-Iqbal I, Iqbal K (1998) Phosphorylation of tau at both Thr 231 and Ser 262 is required for maximal inhibition of its binding to microtubules. Arch Biochem Biophys 357 (2):299-309. doi:10.1006/abbi.1998.0813
115. Nykanen NP, Kysenius K, Sakha P, Tammela P, Huttunen HJ (2012) gamma-Aminobutyric acid type A (GABAA) receptor activation modulates tau phosphorylation. The Journal of biological chemistry 287 (9):6743-6752. doi:10.1074/jbc.M111.309385
116. Fatemi SH (2001) Reelin mutations in mouse and man: from reeler mouse to schizophrenia, mood disorders, autism and lissencephaly. Mol Psychiatry 6 (2):129-133. doi:10.1038/sj.mp.4000129
117. Louis JV, Martens E, Borghgraef P, Lambrecht C, Sents W, Longin S, Zwaenepoel K, Pijnenborg R, Landrieu I, Lippens G, Ledermann B, Gotz J, Van Leuven F, Goris J, Janssens V (2011) Mice lacking phosphatase PP2A subunit PR61/B'delta (Ppp2r5d) develop spatially restricted tauopathy by deregulation of CDK5 and GSK3beta. Proceedings of the National Academy of Sciences of the United States of America 108 (17):6957-6962. doi:10.1073/pnas.1018777108
118. Skube SB, Chaverri JM, Goodson HV (2010) Effect of GFP tags on the localization of EB1 and EB1 fragments in vivo. Cytoskeleton (Hoboken, NJ) 67 (1):1-12. doi:10.1002/cm.20409
119. Kim KT, Ongusaha PP, Hong YK, Kurdistani SK, Nakamura M, Lu KP, Lee SW (2004) Function of Drg1/Rit42 in p53-dependent mitotic spindle checkpoint. The Journal of biological chemistry 279 (37):38597-38602. doi:10.1074/jbc.M400781200
120. Wang XM, Peloquin JG, Zhai Y, Bulinski JC, Borisy GG (1996) Removal of MAP4 from microtubules in vivo produces no observable phenotype at the cellular level. The Journal of cell biology 132 (3):345-357
121. Kitazawa H, Iida J, Uchida A, Haino-Fukushima K, Itoh TJ, Hotani H, Ookata K, Murofushi H, Bulinski JC, Kishimoto T, Hisanaga S (2000) Ser787 in the proline-rich region of human MAP4 is a critical phosphorylation site that reduces its activity to promote tubulin polymerization. Cell structure and function 25 (1):33-39

122. Bogoyevitch MA, Boehm I, Oakley A, Ketterman AJ, Barr RK (2004) Targeting the JNK MAPK cascade for inhibition: basic science and therapeutic potential. Biochim Biophys Acta 1697 (1-2):89-101. doi:10.1016/j.bbapap.2003.11.016
123. Tararuk T, Ostman N, Li W, Bjorkblom B, Padzik A, Zdrojewska J, Hongisto V, Herdegen T, Konopka W, Courtney MJ, Coffey ET (2006) JNK1 phosphorylation of SCG10 determines microtubule dynamics and axodendritic length. J Cell Biol 173 (2):265-277. doi:10.1083/jcb.200511055
124. Lim SH, Kwon SK, Lee MK, Moon J, Jeong DG, Park E, Kim SJ, Park BC, Lee SC, Ryu SE, Yu DY, Chung BH, Kim E, Myung PK, Lee JR (2009) Synapse formation regulated by protein tyrosine phosphatase receptor T through interaction with cell adhesion molecules and Fyn. EMBO J 28 (22):3564-3578. doi:10.1038/emboj.2009.289
125. Teng J, Rai T, Tanaka Y, Takei Y, Nakata T, Hirasawa M, Kulkarni AB, Hirokawa N (2005) The KIF3 motor transports N-cadherin and organizes the developing neuroepithelium. Nature cell biology 7 (5):474-482. doi:10.1038/ncb1249
126. Cabrera JR, Bouzas-Rodriguez J, Tauszig-Delamasure S, Mehlen P (2011) RET modulates cell adhesion via its cleavage by caspase in sympathetic neurons. The Journal of biological chemistry 286 (16):14628-14638. doi:10.1074/jbc.M110.195461
127. Pan PY, Yue Z (2014) Genetic causes of Parkinson's disease and their links to autophagy regulation. Parkinsonism & related disorders 20 Suppl 1:S154-157. doi:10.1016/s1353-8020(13)70037-3
128. Wei Y, Pattingre S, Sinha S, Bassik M, Levine B (2008) JNK1-mediated phosphorylation of Bcl-2 regulates starvation-induced autophagy. Molecular cell 30 (6):678-688. doi:10.1016/j.molcel.2008.06.001
129. Li M, Khambu B, Zhang H, Kang JH, Chen X, Chen D, Vollmer L, Liu PQ, Vogt A, Yin XM (2013) Suppression of lysosome function induces autophagy via a feedback down-regulation of MTOR complex 1 (MTORC1) activity. The Journal of biological chemistry 288 (50):35769-35780. doi:10.1074/jbc.M113.511212
130. Watanabe-Asano T, Kuma A, Mizushima N (2014) Cycloheximide inhibits starvation-induced autophagy through mTORC1 activation. Biochemical and biophysical research communications 445 (2):334-339. doi:10.1016/j.bbrc.2014.01.180
131. Peterson RT, Desai BN, Hardwick JS, Schreiber SL (1999) Protein phosphatase 2A interacts with the 70-kDa S6 kinase and is activated by inhibition of FKBP12-rapamycinassociated protein. Proceedings of the National Academy of Sciences of the United States of America 96 (8):4438-4442
132. Oh WJ, Wu CC, Kim SJ, Facchinetti V, Julien LA, Finlan M, Roux PP, Su B, Jacinto E (2010) mTORC2 can associate with ribosomes to promote cotranslational phosphorylation and stability of nascent Akt polypeptide. EMBO J 29 (23):3939-3951. doi:10.1038/emboj.2010.271
133. Smith PD, Crocker SJ, Jackson-Lewis V, Jordan-Sciutto KL, Hayley S, Mount MP, O'Hare MJ, Callaghan S, Slack RS, Przedborski S, Anisman H, Park DS (2003) Cyclin-dependent kinase 5 is a mediator of dopaminergic neuron loss in a mouse model of Parkinson's disease. Proc Natl Acad Sci U S A 100 (23):13650-13655. doi:10.1073/pnas.2232515100
134. Qu D, Rashidian J, Mount MP, Aleyasin H, Parsanejad M, Lira A, Haque E, Zhang Y, Callaghan S, Daigle M, Rousseaux MW, Slack RS, Albert PR, Vincent I, Woulfe JM, Park DS (2007) Role of Cdk5-mediated phosphorylation of Prx2 in MPTP toxicity and Parkinson's disease. Neuron 55 (1):37-52. doi:10.1016/j.neuron.2007.05.033
135. Wong AS, Lee RH, Cheung AY, Yeung PK, Chung SK, Cheung ZH, Ip NY (2011) Cdk5-mediated phosphorylation of endophilin B1 is required for induced autophagy in models of Parkinson's disease. Nature cell biology 13 (5):568-579. doi:10.1038/ncb2217
136. Schapira AH (2013) Calcium dysregulation in Parkinson's disease. Brain : a journal of neurology 136 (Pt 7):2015-2016. doi:10.1093/brain/awt180
137. Lipton SA (2004) Failures and successes of NMDA receptor antagonists: molecular basis for the use of open-channel blockers like memantine in the treatment of acute and chronic

neurologic insults. NeuroRx : the journal of the American Society for Experimental NeuroTherapeutics 1 (1):101-110. doi:10.1602/neurorx.1.1.101
138. Catterall WA, Perez-Reyes E, Snutch TP, Striessnig J (2005) International Union of Pharmacology. XLVIII. Nomenclature and structure-function relationships of voltage-gated calcium channels. Pharmacological reviews 57 (4):411-425. doi:10.1124/pr.57.4.5
139. Kofuji P, Hadley RW, Kieval RS, Lederer WJ, Schulze DH (1992) Expression of the Na-Ca exchanger in diverse tissues: a study using the cloned human cardiac Na-Ca exchanger. The American journal of physiology 263 (6 Pt 1):C1241-1249
140. Brini M, Manni S, Carafoli E (2002) Recombinant expression of the plasma membrane Na(+)/Ca(2+) exchanger affects local and global Ca(2+) homeostasis in Chinese hamster ovary cells. J Biol Chem 277 (41):38693-38699. doi:10.1074/jbc.M206075200
141. Lencesova L, O'Neill A, Resneck WG, Bloch RJ, Blaustein MP (2004) Plasma membrane-cytoskeleton-endoplasmic reticulum complexes in neurons and astrocytes. J Biol Chem 279 (4):2885-2893. doi:10.1074/jbc.M310365200
142. Kahle JJ, Gulbahce N, Shaw CA, Lim J, Hill DE, Barabasi AL, Zoghbi HY (2011) Comparison of an expanded ataxia interactome with patient medical records reveals a relationship between macular degeneration and ataxia. Hum Mol Genet 20 (3):510-527. doi:10.1093/hmg/ddq496
143. Zemni R, Bienvenu T, Vinet MC, Sefiani A, Carrie A, Billuart P, McDonell N, Couvert P, Francis F, Chafey P, Fauchereau F, Friocourt G, des Portes V, Cardona A, Frints S, Meindl A, Brandau O, Ronce N, Moraine C, van Bokhoven H, Ropers HH, Sudbrak R, Kahn A, Fryns JP, Beldjord C, Chelly J (2000) A new gene involved in X-linked mental retardation identified by analysis of an X;2 balanced translocation. Nature genetics 24 (2):167-170. doi:10.1038/72829
144. Bajic A, Zakrzewska J, Godjevac D, Andjus P, Jones DR, Spasic M, Spasojevic I (2011) Relevance of the ability of fructose 1,6-bis(phosphate) to sequester ferrous but not ferric ions. Carbohydrate research 346 (3):416-420. doi:10.1016/j.carres.2010.12.008
145. Wei FY, Nagashima K, Ohshima T, Saheki Y, Lu YF, Matsushita M, Yamada Y, Mikoshiba K, Seino Y, Matsui H, Tomizawa K (2005) Cdk5-dependent regulation of glucose-stimulated insulin secretion. Nat Med 11 (10):1104-1108. doi:10.1038/nm1299
146. Tomizawa K, Ohta J, Matsushita M, Moriwaki A, Li ST, Takei K, Matsui H (2002) Cdk5/p35 regulates neurotransmitter release through phosphorylation and downregulation of P/Q-type voltage-dependent calcium channel activity. J Neurosci 22 (7):2590-2597. doi:20026252
147. An S, Bleu T, Zheng Y, Goetzl EJ (1998) Recombinant human G protein-coupled lysophosphatidic acid receptors mediate intracellular calcium mobilization. Mol Pharmacol 54 (5):881-888
148. Richardson RM, Marjoram RJ, Barr AJ, Snyderman R (2001) RGS4 inhibits platelet-activating factor receptor phosphorylation and cellular responses. Biochemistry 40 (12):3583-3588
149. De Vries L, Zheng B, Fischer T, Elenko E, Farquhar MG (2000) The regulator of G protein signaling family. Annual review of pharmacology and toxicology 40:235-271. doi:10.1146/annurev.pharmtox.40.1.235
150. Cottrell GS, Padilla B, Pikios S, Roosterman D, Steinhoff M, Grady EF, Bunnett NW (2007) Post-endocytic sorting of calcitonin receptor-like receptor and receptor activity-modifying protein 1. The Journal of biological chemistry 282 (16):12260-12271. doi:10.1074/jbc.M606338200
151. Ko HW, Park KY, Kim H, Han PL, Kim YU, Gwag BJ, Choi EJ (1998) Ca2+-mediated activation of c-Jun N-terminal kinase and nuclear factor kappa B by NMDA in cortical cell cultures. J Neurochem 71 (4):1390-1395
152. Mathisen PM, Johnson JM, Kawczak JA, Tuohy VK (1999) Visinin-like protein (VILIP) is a neuron-specific calcium-dependent double-stranded RNA-binding protein. J Biol Chem 274 (44):31571-31576
153. Braunewell KH (2012) The visinin-like proteins VILIP-1 and VILIP-3 in Alzheimer's disease-old wine in new bottles. Front Mol Neurosci 5:20. doi:10.3389/fnmol.2012.00020
154. Matveeva EA, Whiteheart SW, Vanaman TC, Slevin JT (2001) Phosphorylation of the N-ethylmaleimide-sensitive factor is associated with depolarization-dependent

neurotransmitter release from synaptosomes. J Biol Chem 276 (15):12174-12181. doi:10.1074/jbc.M007394200
155. Salnikow K, Kluz T, Costa M, Piquemal D, Demidenko ZN, Xie K, Blagosklonny MV (2002) The regulation of hypoxic genes by calcium involves c-Jun/AP-1, which cooperates with hypoxia-inducible factor 1 in response to hypoxia. Mol Cell Biol 22 (6):1734-1741
156. Cox JA, Durussel I, Comte M, Nef S, Nef P, Lenz SE, Gundelfinger ED (1994) Cation binding and conformational changes in VILIP and NCS-1, two neuron-specific calcium-binding proteins. J Biol Chem 269 (52):32807-32813
157. Lee-Young RS, Griffee SR, Lynes SE, Bracy DP, Ayala JE, McGuinness OP, Wasserman DH (2009) Skeletal muscle AMP-activated protein kinase is essential for the metabolic response to exercise in vivo. J Biol Chem 284 (36):23925-23934. doi:10.1074/jbc.M109.021048
158. Perez RG, Waymire JC, Lin E, Liu JJ, Guo F, Zigmond MJ (2002) A role for alpha-synuclein in the regulation of dopamine biosynthesis. J Neurosci 22 (8):3090-3099. doi:20026307
159. McFarland MA, Ellis CE, Markey SP, Nussbaum RL (2008) Proteomics analysis identifies phosphorylation-dependent alpha-synuclein protein interactions. Mol Cell Proteomics 7 (11):2123-2137. doi:10.1074/mcp.M800116-MCP200
160. Pan ZZ, Bruening W, Giasson BI, Lee VM, Godwin AK (2002) Gamma-synuclein promotes cancer cell survival and inhibits stress- and chemotherapy drug-induced apoptosis by modulating MAPK pathways. J Biol Chem 277 (38):35050-35060. doi:10.1074/jbc.M201650200
161. Woods WS, Boettcher JM, Zhou DH, Kloepper KD, Hartman KL, Ladror DT, Qi Z, Rienstra CM, George JM (2007) Conformation-specific binding of alpha-synuclein to novel protein partners detected by phage display and NMR spectroscopy. J Biol Chem 282 (47):34555-34567. doi:10.1074/jbc.M705283200
162. Nakamura T, Yamashita H, Takahashi T, Nakamura S (2001) Activated Fyn phosphorylates alpha-synuclein at tyrosine residue 125. Biochem Biophys Res Commun 280 (4):1085-1092. doi:10.1006/bbrc.2000.4253
163. Ellis CE, Schwartzberg PL, Grider TL, Fink DW, Nussbaum RL (2001) alpha-synuclein is phosphorylated by members of the Src family of protein-tyrosine kinases. J Biol Chem 276 (6):3879-3884. doi:10.1074/jbc.M010316200
164. Burre J, Sharma M, Tsetsenis T, Buchman V, Etherton MR, Sudhof TC (2010) Alpha-synuclein promotes SNARE-complex assembly in vivo and in vitro. Science 329 (5999):1663-1667. doi:10.1126/science.1195227
165. Diao J, Burre J, Vivona S, Cipriano DJ, Sharma M, Kyoung M, Sudhof TC, Brunger AT (2013) Native alpha-synuclein induces clustering of synaptic-vesicle mimics via binding to phospholipids and synaptobrevin-2/VAMP2. Elife 2:e00592. doi:10.7554/eLife.00592
166. Hashimoto M, Rockenstein E, Mante M, Mallory M, Masliah E (2001) beta-Synuclein inhibits alpha-synuclein aggregation: a possible role as an anti-parkinsonian factor. Neuron 32 (2):213-223
167. Volles MJ, Lansbury PT, Jr. (2003) Zeroing in on the pathogenic form of alpha-synuclein and its mechanism of neurotoxicity in Parkinson's disease. Biochemistry 42 (26):7871-7878. doi:10.1021/bi030086j
168. Hashimoto M, Hsu LJ, Rockenstein E, Takenouchi T, Mallory M, Masliah E (2002) alpha-Synuclein protects against oxidative stress via inactivation of the c-Jun N-terminal kinase stress-signaling pathway in neuronal cells. J Biol Chem 277 (13):11465-11472. doi:10.1074/jbc.M111428200
169. Nakata Y, Yasuda T, Fukaya M, Yamamori S, Itakura M, Nihira T, Hayakawa H, Kawanami A, Kataoka M, Nagai M, Sakagami H, Takahashi M, Mizuno Y, Mochizuki H (2012) Accumulation of alpha-synuclein triggered by presynaptic dysfunction. J Neurosci 32 (48):17186-17196. doi:10.1523/jneurosci.2220-12.2012
170. Usami Y, Hatano T, Imai S, Kubo S, Sato S, Saiki S, Fujioka Y, Ohba Y, Sato F, Funayama M, Eguchi H, Shiba K, Ariga H, Shen J, Hattori N (2011) DJ-1 associates with synaptic membranes. Neurobiol Dis 43 (3):651-662. doi:10.1016/j.nbd.2011.05.014

171. Piccoli G, Condliffe SB, Bauer M, Giesert F, Boldt K, De Astis S, Meixner A, Sarioglu H, Vogt-Weisenhorn DM, Wurst W, Gloeckner CJ, Matteoli M, Sala C, Ueffing M (2011) LRRK2 controls synaptic vesicle storage and mobilization within the recycling pool. J Neurosci 31 (6):2225-2237. doi:10.1523/jneurosci.3730-10.2011
172. Gloeckner CJ, Schumacher A, Boldt K, Ueffing M (2009) The Parkinson disease-associated protein kinase LRRK2 exhibits MAPKKK activity and phosphorylates MKK3/6 and MKK4/7, in vitro. J Neurochem 109 (4):959-968. doi:10.1111/j.1471-4159.2009.06024.x
173. Meixner A, Boldt K, Van Troys M, Askenazi M, Gloeckner CJ, Bauer M, Marto JA, Ampe C, Kinkl N, Ueffing M (2011) A QUICK screen for Lrrk2 interaction partners--leucine-rich repeat kinase 2 is involved in actin cytoskeleton dynamics. Molecular & cellular proteomics : MCP 10 (1):M110.001172. doi:10.1074/mcp.M110.001172
174. Nichols RJ, Dzamko N, Morrice NA, Campbell DG, Deak M, Ordureau A, Macartney T, Tong Y, Shen J, Prescott AR, Alessi DR (2010) 14-3-3 binding to LRRK2 is disrupted by multiple Parkinson's disease-associated mutations and regulates cytoplasmic localization. Biochem J 430 (3):393-404. doi:10.1042/bj20100483
175. Ohta E, Kawakami F, Kubo M, Obata F (2011) LRRK2 directly phosphorylates Akt1 as a possible physiological substrate: impairment of the kinase activity by Parkinson's disease-associated mutations. FEBS Lett 585 (14):2165-2170. doi:10.1016/j.febslet.2011.05.044
176. Avraham E, Rott R, Liani E, Szargel R, Engelender S (2007) Phosphorylation of Parkin by the cyclin-dependent kinase 5 at the linker region modulates its ubiquitin-ligase activity and aggregation. J Biol Chem 282 (17):12842-12850. doi:10.1074/jbc.M608243200
177. Van Humbeeck C, Cornelissen T, Hofkens H, Mandemakers W, Gevaert K, De Strooper B, Vandenberghe W (2011) Parkin interacts with Ambra1 to induce mitophagy. The Journal of neuroscience : the official journal of the Society for Neuroscience 31 (28):10249-10261. doi:10.1523/jneurosci.1917-11.2011
178. Cha GH, Kim S, Park J, Lee E, Kim M, Lee SB, Kim JM, Chung J, Cho KS (2005) Parkin negatively regulates JNK pathway in the dopaminergic neurons of Drosophila. Proc Natl Acad Sci U S A 102 (29):10345-10350. doi:10.1073/pnas.0500346102
179. Liu Y, Fallon L, Lashuel HA, Liu Z, Lansbury PT, Jr. (2002) The UCH-L1 gene encodes two opposing enzymatic activities that affect alpha-synuclein degradation and Parkinson's disease susceptibility. Cell 111 (2):209-218
180. Caballero OL, Resto V, Patturajan M, Meerzaman D, Guo MZ, Engles J, Yochem R, Ratovitski E, Sidransky D, Jen J (2002) Interaction and colocalization of PGP9.5 with JAB1 and p27(Kip1). Oncogene 21 (19):3003-3010. doi:10.1038/sj.onc.1205390
181. Hussain S, Feldman AL, Das C, Ziesmer SC, Ansell SM, Galardy PJ (2013) Ubiquitin hydrolase UCH-L1 destabilizes mTOR complex 1 by antagonizing DDB1-CUL4-mediated ubiquitination of raptor. Mol Cell Biol 33 (6):1188-1197. doi:10.1128/mcb.01389-12
182. Wilker EW, van Vugt MA, Artim SA, Huang PH, Petersen CP, Reinhardt HC, Feng Y, Sharp PA, Sonenberg N, White FM, Yaffe MB (2007) 14-3-3sigma controls mitotic translation to facilitate cytokinesis. Nature 446 (7133):329-332. doi:10.1038/nature05584
183. Matenia D, Hempp C, Timm T, Eikhof A, Mandelkow EM (2012) Microtubule affinity-regulating kinase 2 (MARK2) turns on phosphatase and tensin homolog (PTEN)-induced kinase 1 (PINK1) at Thr-313, a mutation site in Parkinson disease: effects on mitochondrial transport. J Biol Chem 287 (11):8174-8186. doi:10.1074/jbc.M111.262287
184. Moran LB, Duke DC, Deprez M, Dexter DT, Pearce RK, Graeber MB (2006) Whole genome expression profiling of the medial and lateral substantia nigra in Parkinson's disease. Neurogenetics 7 (1):1-11. doi:10.1007/s10048-005-0020-2
185. Chevrier N, Mertins P, Artyomov MN, Shalek AK, Iannacone M, Ciaccio MF, Gat-Viks I, Tonti E, DeGrace MM, Clauser KR, Garber M, Eisenhaure TM, Yosef N, Robinson J, Sutton A, Andersen MS, Root DE, von Andrian U, Jones RB, Park H, Carr SA, Regev A, Amit I, Hacohen N (2011) Systematic discovery of TLR signaling components delineates viral-sensing circuits. Cell 147 (4):853-867. doi:10.1016/j.cell.2011.10.022

186. Kim HN, Lee JH, Bae SC, Ryoo HM, Kim HH, Ha H, Lee ZH (2011) Histone deacetylase inhibitor MS-275 stimulates bone formation in part by enhancing Dhx36-mediated TNAP transcription. J Bone Miner Res 26 (9):2161-2173. doi:10.1002/jbmr.426
187. Leone V, Mansueto G, Pierantoni GM, Tornincasa M, Merolla F, Cerrato A, Santoro M, Grieco M, Scaloni A, Celetti A, Fusco A (2010) CCDC6 represses CREB1 activity by recruiting histone deacetylase 1 and protein phosphatase 1. Oncogene 29 (30):4341-4351. doi:10.1038/onc.2010.179
188. Opsahl JA, Hjornevik LV, Bull VH, Fismen L, Froyset AK, Gromyko D, Solstad T, Fladmark KE (2010) Increased interaction between DJ-1 and the Mi-2/ nucleosome remodelling and deacetylase complex during cellular stress. Proteomics 10 (7):1494-1504. doi:10.1002/pmic.200900586
189. Morrison BE, Majdzadeh N, Zhang X, Lyles A, Bassel-Duby R, Olson EN, D'Mello SR (2006) Neuroprotection by histone deacetylase-related protein. Mol Cell Biol 26 (9):3550-3564. doi:10.1128/mcb.26.9.3550-3564.2006
190. Shimazu T, Horinouchi S, Yoshida M (2007) Multiple histone deacetylases and the CREB-binding protein regulate pre-mRNA 3'-end processing. J Biol Chem 282 (7):4470-4478. doi:10.1074/jbc.M609745200
191. Dobbin MM, Madabhushi R, Pan L, Chen Y, Kim D, Gao J, Ahanonu B, Pao PC, Qiu Y, Zhao Y, Tsai LH (2013) SIRT1 collaborates with ATM and HDAC1 to maintain genomic stability in neurons. Nature neuroscience 16 (8):1008-1015. doi:10.1038/nn.3460
192. Wen YD, Cress WD, Roy AL, Seto E (2003) Histone deacetylase 3 binds to and regulates the multifunctional transcription factor TFII-I. J Biol Chem 278 (3):1841-1847. doi:10.1074/jbc.M206528200
193. Wilson BJ, Bates GJ, Nicol SM, Gregory DJ, Perkins ND, Fuller-Pace FV (2004) The p68 and p72 DEAD box RNA helicases interact with HDAC1 and repress transcription in a promoter-specific manner. BMC molecular biology 5:11. doi:10.1186/1471-2199-5-11
194. Aguilera C, Hoya-Arias R, Haegeman G, Espinosa L, Bigas A (2004) Recruitment of IkappaBalpha to the hes1 promoter is associated with transcriptional repression. Proceedings of the National Academy of Sciences of the United States of America 101 (47):16537-16542. doi:10.1073/pnas.0404429101
195. Engelen E, Akinci U, Bryne JC, Hou J, Gontan C, Moen M, Szumska D, Kockx C, van Ijcken W, Dekkers DH, Demmers J, Rijkers EJ, Bhattacharya S, Philipsen S, Pevny LH, Grosveld FG, Rottier RJ, Lenhard B, Poot RA (2011) Sox2 cooperates with Chd7 to regulate genes that are mutated in human syndromes. Nature genetics 43 (6):607-611. doi:10.1038/ng.825
196. Kitagawa H, Ray WJ, Glantschnig H, Nantermet PV, Yu Y, Leu CT, Harada S, Kato S, Freedman LP (2007) A regulatory circuit mediating convergence between Nurr1 transcriptional regulation and Wnt signaling. Mol Cell Biol 27 (21):7486-7496. doi:10.1128/mcb.00409-07
197. Kumar PP, Purbey PK, Ravi DS, Mitra D, Galande S (2005) Displacement of SATB1-bound histone deacetylase 1 corepressor by the human immunodeficiency virus type 1 transactivator induces expression of interleukin-2 and its receptor in T cells. Molecular and cellular biology 25 (5):1620-1633. doi:10.1128/mcb.25.5.1620-1633.2005
198. Zhong H, May MJ, Jimi E, Ghosh S (2002) The phosphorylation status of nuclear NF-kappa B determines its association with CBP/p300 or HDAC-1. Molecular cell 9 (3):625-636
199. Chen CS, Weng SC, Tseng PH, Lin HP, Chen CS (2005) Histone acetylation-independent effect of histone deacetylase inhibitors on Akt through the reshuffling of protein phosphatase 1 complexes. The Journal of biological chemistry 280 (46):38879-38887. doi:10.1074/jbc.M505733200
200. Bandyopadhyay D, Mishra A, Medrano EE (2004) Overexpression of histone deacetylase 1 confers resistance to sodium butyrate-mediated apoptosis in melanoma cells through a p53-mediated pathway. Cancer research 64 (21):7706-7710. doi:10.1158/0008-5472.can-03-3897
201. Menick DR, Li MS, Chernysh O, Renaud L, Kimbrough D, Kasiganesan H, Mani SK (2013) Transcriptional pathways and potential therapeutic targets in the regulation of Ncx1

expression in cardiac hypertrophy and failure. Advances in experimental medicine and biology 961:125-135. doi:10.1007/978-1-4614-4756-6_11
202. Zhang A, Wang H, Qin X, Pang S, Yan B (2012) Genetic analysis of SIRT1 gene promoter in sporadic Parkinson's disease. Biochem Biophys Res Commun 422 (4):693-696. doi:10.1016/j.bbrc.2012.05.059
203. Sowa ME, Bennett EJ, Gygi SP, Harper JW (2009) Defining the human deubiquitinating enzyme interaction landscape. Cell 138 (2):389-403. doi:10.1016/j.cell.2009.04.042
204. Lin Z, Yang H, Kong Q, Li J, Lee SM, Gao B, Dong H, Wei J, Song J, Zhang DD, Fang D (2012) USP22 antagonizes p53 transcriptional activation by deubiquitinating Sirt1 to suppress cell apoptosis and is required for mouse embryonic development. Mol Cell 46 (4):484-494. doi:10.1016/j.molcel.2012.03.024
205. Xue Z, Lv X, Song W, Wang X, Zhao GN, Wang WT, Xiong J, Mao BB, Yu W, Yang B, Wu J, Zhou LQ, Hao DL, Dong WJ, Liu DP, Liang CC (2012) SIRT1 deacetylates SATB1 to facilitate MAR HS2-MAR epsilon interaction and promote epsilon-globin expression. Nucleic acids research 40 (11):4804-4815. doi:10.1093/nar/gks064
206. Tanikawa M, Wada-Hiraike O, Nakagawa S, Shirane A, Hiraike H, Koyama S, Miyamoto Y, Sone K, Tsuruga T, Nagasaka K, Matsumoto Y, Ikeda Y, Shoji K, Oda K, Fukuhara H, Nakagawa K, Kato S, Yano T, Taketani Y (2011) Multifunctional transcription factor TFII-I is an activator of BRCA1 function. Br J Cancer 104 (8):1349-1355. doi:10.1038/bjc.2011.75
207. Nasrin N, Kaushik VK, Fortier E, Wall D, Pearson KJ, de Cabo R, Bordone L (2009) JNK1 phosphorylates SIRT1 and promotes its enzymatic activity. PLoS One 4 (12):e8414. doi:10.1371/journal.pone.0008414
208. Dumitriu A, Latourelle JC, Hadzi TC, Pankratz N, Garza D, Miller JP, Vance JM, Foroud T, Beach TG, Myers RH (2012) Gene expression profiles in Parkinson disease prefrontal cortex implicate FOXO1 and genes under its transcriptional regulation. PLoS Genet 8 (6):e1002794. doi:10.1371/journal.pgen.1002794
209. Daitoku H, Hatta M, Matsuzaki H, Aratani S, Ohshima T, Miyagishi M, Nakajima T, Fukamizu A (2004) Silent information regulator 2 potentiates Foxo1-mediated transcription through its deacetylase activity. Proc Natl Acad Sci U S A 101 (27):10042-10047. doi:10.1073/pnas.0400593101
210. Yang Y, Hou H, Haller EM, Nicosia SV, Bai W (2005) Suppression of FOXO1 activity by FHL2 through SIRT1-mediated deacetylation. EMBO J 24 (5):1021-1032. doi:10.1038/sj.emboj.7600570
211. Xiong S, Salazar G, Patrushev N, Alexander RW (2011) FoxO1 mediates an autofeedback loop regulating SIRT1 expression. J Biol Chem 286 (7):5289-5299. doi:10.1074/jbc.M110.163667
212. Gao Z, Zhang J, Kheterpal I, Kennedy N, Davis RJ, Ye J (2011) Sirtuin 1 (SIRT1) protein degradation in response to persistent c-Jun N-terminal kinase 1 (JNK1) activation contributes to hepatic steatosis in obesity. J Biol Chem 286 (25):22227-22234. doi:10.1074/jbc.M111.228874
213. Inberg A, Linial M (2010) Protection of pancreatic beta-cells from various stress conditions is mediated by DJ-1. J Biol Chem 285 (33):25686-25698. doi:10.1074/jbc.M110.109751
214. Zhu QS, Rosenblatt K, Huang KL, Lahat G, Brobey R, Bolshakov S, Nguyen T, Ding Z, Belousov R, Bill K, Luo X, Lazar A, Dicker A, Mills GB, Hung MC, Lev D (2011) Vimentin is a novel AKT1 target mediating motility and invasion. Oncogene 30 (4):457-470. doi:10.1038/onc.2010.421
215. Mogi M, Kondo T, Mizuno Y, Nagatsu T (2007) p53 protein, interferon-gamma, and NF-kappaB levels are elevated in the parkinsonian brain. Neurosci Lett 414 (1):94-97. doi:10.1016/j.neulet.2006.12.003
216. Huxford T, Huang DB, Malek S, Ghosh G (1998) The crystal structure of the IkappaBalpha/NF-kappaB complex reveals mechanisms of NF-kappaB inactivation. Cell 95 (6):759-770
217. Ludwig L, Kessler H, Wagner M, Hoang-Vu C, Dralle H, Adler G, Bohm BO, Schmid RM (2001) Nuclear factor-kappaB is constitutively active in C-cell carcinoma and required for RET-induced transformation. Cancer research 61 (11):4526-4535

218. Cannons JL, Yu LJ, Hill B, Mijares LA, Dombroski D, Nichols KE, Antonellis A, Koretzky GA, Gardner K, Schwartzberg PL (2004) SAP regulates T(H)2 differentiation and PKC-theta-mediated activation of NF-kappaB1. Immunity 21 (5):693-706. doi:10.1016/j.immuni.2004.09.012
219. Kang KW, Choi SY, Cho MK, Lee CH, Kim SG (2003) Thrombin induces nitric-oxide synthase via Galpha12/13-coupled protein kinase C-dependent I-kappaBalpha phosphorylation and JNK-mediated I-kappaBalpha degradation. J Biol Chem 278 (19):17368-17378. doi:10.1074/jbc.M300471200
220. de Moissac D, Zheng H, Kirshenbaum LA (1999) Linkage of the BH4 domain of Bcl-2 and the nuclear factor kappaB signaling pathway for suppression of apoptosis. The Journal of biological chemistry 274 (41):29505-29509
221. Hotta K, Nashimoto A, Yasumura E, Suzuki M, Azuma M, Iizumi Y, Shima D, Nabeshima R, Hiramoto M, Okada A, Sakata-Sogawa K, Tokunaga M, Ito T, Ando H, Sakamoto S, Kabe Y, Aizawa S, Imai T, Yamaguchi Y, Watanabe H, Handa H (2013) Vesnarinone suppresses TNFalpha mRNA expression by inhibiting valosin-containing protein. Molecular pharmacology 83 (5):930-938. doi:10.1124/mol.112.081935
222. Wang Q, Zhou Y, Wang X, Chung DH, Evers BM (2007) Regulation of PTEN expression in intestinal epithelial cells by c-Jun NH2-terminal kinase activation and nuclear factor-kappaB inhibition. Cancer Res 67 (16):7773-7781. doi:10.1158/0008-5472.can-07-0187
223. Loercher A, Lee TL, Ricker JL, Howard A, Geoghegen J, Chen Z, Sunwoo JB, Sitcheran R, Chuang EY, Mitchell JB, Baldwin AS, Jr., Van Waes C (2004) Nuclear factor-kappaB is an important modulator of the altered gene expression profile and malignant phenotype in squamous cell carcinoma. Cancer research 64 (18):6511-6523. doi:10.1158/0008-5472.can-04-0852
224. Zhang HG, Hyde K, Page GP, Brand JP, Zhou J, Yu S, Allison DB, Hsu HC, Mountz JD (2004) Novel tumor necrosis factor alpha-regulated genes in rheumatoid arthritis. Arthritis and rheumatism 50 (2):420-431. doi:10.1002/art.20037
225. Guo G, Yan-Sanders Y, Lyn-Cook BD, Wang T, Tamae D, Ogi J, Khaletskiy A, Li Z, Weydert C, Longmate JA, Huang TT, Spitz DR, Oberley LW, Li JJ (2003) Manganese superoxide dismutase-mediated gene expression in radiation-induced adaptive responses. Mol Cell Biol 23 (7):2362-2378
226. Rena G, Prescott AR, Guo S, Cohen P, Unterman TG (2001) Roles of the forkhead in rhabdomyosarcoma (FKHR) phosphorylation sites in regulating 14-3-3 binding, transactivation and nuclear targetting. Biochem J 354 (Pt 3):605-612
227. Lautier C, Goldwurm S, Durr A, Giovannone B, Tsiaras WG, Pezzoli G, Brice A, Smith RJ (2008) Mutations in the GIGYF2 (TNRC15) gene at the PARK11 locus in familial Parkinson disease. American journal of human genetics 82 (4):822-833. doi:10.1016/j.ajhg.2008.01.015
228. Xiromerisiou G, Hadjigeorgiou GM, Papadimitriou A, Katsarogiannis E, Gourbali V, Singleton AB (2008) Association between AKT1 gene and Parkinson's disease: a protective haplotype. Neurosci Lett 436 (2):232-234. doi:10.1016/j.neulet.2008.03.026
229. Zhou Y, Gu G, Goodlett DR, Zhang T, Pan C, Montine TJ, Montine KS, Aebersold RH, Zhang J (2004) Analysis of alpha-synuclein-associated proteins by quantitative proteomics. The Journal of biological chemistry 279 (37):39155-39164. doi:10.1074/jbc.M405456200
230. Jankovic J, Chen S, Le WD (2005) The role of Nurr1 in the development of dopaminergic neurons and Parkinson's disease. Progress in neurobiology 77 (1-2):128-138. doi:10.1016/j.pneurobio.2005.09.001
231. Liu X, Li F, Stubblefield EA, Blanchard B, Richards TL, Larson GA, He Y, Huang Q, Tan AC, Zhang D, Benke TA, Sladek JR, Zahniser NR, Li CY (2012) Direct reprogramming of human fibroblasts into dopaminergic neuron-like cells. Cell research 22 (2):321-332. doi:10.1038/cr.2011.181
232. Kadkhodaei B, Ito T, Joodmardi E, Mattsson B, Rouillard C, Carta M, Muramatsu S, Sumi-Ichinose C, Nomura T, Metzger D, Chambon P, Lindqvist E, Larsson NG, Olson L, Bjorklund A, Ichinose H, Perlmann T (2009) Nurr1 is required for maintenance of maturing and adult midbrain

dopamine neurons. The Journal of neuroscience : the official journal of the Society for Neuroscience 29 (50):15923-15932. doi:10.1523/jneurosci.3910-09.2009
233. Isacson O (2003) The production and use of cells as therapeutic agents in neurodegenerative diseases. Lancet neurology 2 (7):417-424
234. Yang YX, Latchman DS (2008) Nurr1 transcriptionally regulates the expression of alpha-synuclein. Neuroreport 19 (8):867-871. doi:10.1097/WNR.0b013e3282ffda48
235. Miyamoto-Sato E, Fujimori S, Ishizaka M, Hirai N, Masuoka K, Saito R, Ozawa Y, Hino K, Washio T, Tomita M, Yamashita T, Oshikubo T, Akasaka H, Sugiyama J, Matsumoto Y, Yanagawa H (2010) A comprehensive resource of interacting protein regions for refining human transcription factor networks. PLoS One 5 (2):e9289. doi:10.1371/journal.pone.0009289
236. Saijo K, Winner B, Carson CT, Collier JG, Boyer L, Rosenfeld MG, Gage FH, Glass CK (2009) A Nurr1/CoREST pathway in microglia and astrocytes protects dopaminergic neurons from inflammation-induced death. Cell 137 (1):47-59. doi:10.1016/j.cell.2009.01.038
237. Ahlfors H, Limaye A, Elo LL, Tuomela S, Burute M, Gottimukkala KV, Notani D, Rasool O, Galande S, Lahesmaa R (2010) SATB1 dictates expression of multiple genes including IL-5 involved in human T helper cell differentiation. Blood 116 (9):1443-1453. doi:10.1182/blood-2009-11-252205
238. Cox JL, Wilder PJ, Gilmore JM, Wuebben EL, Washburn MP, Rizzino A (2013) The SOX2-interactome in brain cancer cells identifies the requirement of MSI2 and USP9X for the growth of brain tumor cells. PloS one 8 (5):e62857. doi:10.1371/journal.pone.0062857
239. Peltier J, Conway A, Keung AJ, Schaffer DV (2011) Akt increases sox2 expression in adult hippocampal neural progenitor cells, but increased sox2 does not promote proliferation. Stem cells and development 20 (7):1153-1161. doi:10.1089/scd.2010.0130
240. Zhang X, Yalcin S, Lee DF, Yeh TY, Lee SM, Su J, Mungamuri SK, Rimmele P, Kennedy M, Sellers R, Landthaler M, Tuschl T, Chi NW, Lemischka I, Keller G, Ghaffari S (2011) FOXO1 is an essential regulator of pluripotency in human embryonic stem cells. Nature cell biology 13 (9):1092-1099. doi:10.1038/ncb2293
241. Simpson L, Li J, Liaw D, Hennessy I, Oliner J, Christians F, Parsons R (2001) PTEN expression causes feedback upregulation of insulin receptor substrate 2. Mol Cell Biol 21 (12):3947-3958. doi:10.1128/mcb.21.12.3947-3958.2001
242. Melillo RM, Carlomagno F, De Vita G, Formisano P, Vecchio G, Fusco A, Billaud M, Santoro M (2001) The insulin receptor substrate (IRS)-1 recruits phosphatidylinositol 3-kinase to Ret: evidence for a competition between Shc and IRS-1 for the binding to Ret. Oncogene 20 (2):209-218. doi:10.1038/sj.onc.1204049
243. Li J, Davidson G, Huang Y, Jiang BH, Shi X, Costa M, Huang C (2004) Nickel compounds act through phosphatidylinositol-3-kinase/Akt-dependent, p70(S6k)-independent pathway to induce hypoxia inducible factor transactivation and Cap43 expression in mouse epidermal Cl41 cells. Cancer Res 64 (1):94-101
244. Ge Y, Yoon MS, Chen J (2011) Raptor and Rheb negatively regulate skeletal myogenesis through suppression of insulin receptor substrate 1 (IRS1). J Biol Chem 286 (41):35675-35682. doi:10.1074/jbc.M111.262881
245. Matsushima-Nishiu M, Unoki M, Ono K, Tsunoda T, Minaguchi T, Kuramoto H, Nishida M, Satoh T, Tanaka T, Nakamura Y (2001) Growth and gene expression profile analyses of endometrial cancer cells expressing exogenous PTEN. Cancer Res 61 (9):3741-3749
246. Bandyopadhyay S, Pai SK, Hirota S, Hosobe S, Tsukada T, Miura K, Takano Y, Saito K, Commes T, Piquemal D, Watabe M, Gross S, Wang Y, Huggenvik J, Watabe K (2004) PTEN up-regulates the tumor metastasis suppressor gene Drg-1 in prostate and breast cancer. Cancer Res 64 (21):7655-7660. doi:10.1158/0008-5472.can-04-1623
247. Domanskyi A, Geissler C, Vinnikov IA, Alter H, Schober A, Vogt MA, Gass P, Parlato R, Schutz G (2011) Pten ablation in adult dopaminergic neurons is neuroprotective in Parkinson's disease models. FASEB J 25 (9):2898-2910. doi:10.1096/fj.11-181958

248. Dupont J, Renou JP, Shani M, Hennighausen L, LeRoith D (2002) PTEN overexpression suppresses proliferation and differentiation and enhances apoptosis of the mouse mammary epithelium. J Clin Invest 110 (6):815-825. doi:10.1172/jci13829
249. Janas MM, Wang E, Love T, Harris AS, Stevenson K, Semmelmann K, Shaffer JM, Chen PH, Doench JG, Yerramilli SV, Neuberg DS, Iliopoulos D, Housman DE, Burge CB, Novina CD (2012) Reduced expression of ribosomal proteins relieves microRNA-mediated repression. Mol Cell 46 (2):171-186. doi:10.1016/j.molcel.2012.04.008
250. Paliwal S, Kovi RC, Nath B, Chen YW, Lewis BC, Grossman SR (2007) The alternative reading frame tumor suppressor antagonizes hypoxia-induced cancer cell migration via interaction with the COOH-terminal binding protein corepressor. Cancer Res 67 (19):9322-9329. doi:10.1158/0008-5472.can-07-1743
251. Singh G, Kucukural A, Cenik C, Leszyk JD, Shaffer SA, Weng Z, Moore MJ (2012) The cellular EJC interactome reveals higher-order mRNP structure and an EJC-SR protein nexus. Cell 151 (4):750-764. doi:10.1016/j.cell.2012.10.007
252. Varjosalo M, Keskitalo S, Van Drogen A, Nurkkala H, Vichalkovski A, Aebersold R, Gstaiger M (2013) The protein interaction landscape of the human CMGC kinase group. Cell reports 3 (4):1306-1320. doi:10.1016/j.celrep.2013.03.027
253. Jung SY, Malovannaya A, Wei J, O'Malley BW, Qin J (2005) Proteomic analysis of steady-state nuclear hormone receptor coactivator complexes. Molecular endocrinology (Baltimore, Md) 19 (10):2451-2465. doi:10.1210/me.2004-0476
254. Hegele A, Kamburov A, Grossmann A, Sourlis C, Wowro S, Weimann M, Will CL, Pena V, Luhrmann R, Stelzl U (2012) Dynamic protein-protein interaction wiring of the human spliceosome. Mol Cell 45 (4):567-580. doi:10.1016/j.molcel.2011.12.034
255. Wang HY, Lin W, Dyck JA, Yeakley JM, Songyang Z, Cantley LC, Fu XD (1998) SRPK2: a differentially expressed SR protein-specific kinase involved in mediating the interaction and localization of pre-mRNA splicing factors in mammalian cells. The Journal of cell biology 140 (4):737-750
256. Hong Y, Chan CB, Kwon IS, Li X, Song M, Lee HP, Liu X, Sompol P, Jin P, Lee HG, Yu SP, Ye K (2012) SRPK2 phosphorylates tau and mediates the cognitive defects in Alzheimer's disease. J Neurosci 32 (48):17262-17272. doi:10.1523/jneurosci.3300-12.2012
257. Nordin A, Larsson E, Holmberg M (2012) The defective splicing caused by the ISCU intron mutation in patients with myopathy with lactic acidosis is repressed by PTBP1 but can be derepressed by IGF2BP1. Human mutation 33 (3):467-470. doi:10.1002/humu.22002
258. Popielarz M, Cavaloc Y, Mattei MG, Gattoni R, Stevenin J (1995) The gene encoding human splicing factor 9G8. Structure, chromosomal localization, and expression of alternatively processed transcripts. The Journal of biological chemistry 270 (30):17830-17835
259. Huang Y, Steitz JA (2001) Splicing factors SRp20 and 9G8 promote the nucleocytoplasmic export of mRNA. Molecular cell 7 (4):899-905
260. Wang J, Gao QS, Wang Y, Lafyatis R, Stamm S, Andreadis A (2004) Tau exon 10, whose missplicing causes frontotemporal dementia, is regulated by an intricate interplay of cis elements and trans factors. Journal of neurochemistry 88 (5):1078-1090
261. Hartmann AM, Rujescu D, Giannakouros T, Nikolakaki E, Goedert M, Mandelkow EM, Gao QS, Andreadis A, Stamm S (2001) Regulation of alternative splicing of human tau exon 10 by phosphorylation of splicing factors. Molecular and cellular neurosciences 18 (1):80-90. doi:10.1006/mcne.2001.1000
262. Kovac A, Zilka N, Kazmerova Z, Cente M, Zilkova M, Novak M (2011) Misfolded truncated protein tau induces innate immune response via MAPK pathway. J Immunol 187 (5):2732-2739. doi:10.4049/jimmunol.1100216
263. Zanon A, Rakovic A, Blankenburg H, Doncheva NT, Schwienbacher C, Serafin A, Alexa A, Weichenberger CX, Albrecht M, Klein C, Hicks AA, Pramstaller PP, Domingues FS, Pichler I (2013) Profiling of Parkin-binding partners using tandem affinity purification. PloS one 8 (11):e78648. doi:10.1371/journal.pone.0078648

264. Stelzl U, Worm U, Lalowski M, Haenig C, Brembeck FH, Goehler H, Stroedicke M, Zenkner M, Schoenherr A, Koeppen S, Timm J, Mintzlaff S, Abraham C, Bock N, Kietzmann S, Goedde A, Toksoz E, Droege A, Krobitsch S, Korn B, Birchmeier W, Lehrach H, Wanker EE (2005) A human protein-protein interaction network: a resource for annotating the proteome. Cell 122 (6):957-968. doi:10.1016/j.cell.2005.08.029
265. Brown SJ, Stoilov P, Xing Y (2012) Chromatin and epigenetic regulation of pre-mRNA processing. Human molecular genetics 21 (R1):R90-96. doi:10.1093/hmg/dds353
266. De Conti L, Baralle M, Buratti E (2013) Exon and intron definition in pre-mRNA splicing. Wiley interdisciplinary reviews RNA 4 (1):49-60. doi:10.1002/wrna.1140
267. Schor IE, Allo M, Kornblihtt AR (2010) Intragenic chromatin modifications: A new layer in alternative splicing regulation. Epigenetics : official journal of the DNA Methylation Society 5 (3)
268. Hnilicova J, Hozeifi S, Duskova E, Icha J, Tomankova T, Stanek D (2011) Histone deacetylase activity modulates alternative splicing. PloS one 6 (2):e16727. doi:10.1371/journal.pone.0016727
269. Hanley JG, Khatri L, Hanson PI, Ziff EB (2002) NSF ATPase and alpha-/beta-SNAPs disassemble the AMPA receptor-PICK1 complex. Neuron 34 (1):53-67
270. Diefenbach RJ, Diefenbach E, Douglas MW, Cunningham AL (2002) The heavy chain of conventional kinesin interacts with the SNARE proteins SNAP25 and SNAP23. Biochemistry 41 (50):14906-14915
271. Lashuel HA, Hirling H (2006) Rescuing defective vesicular trafficking protects against alpha-synuclein toxicity in cellular and animal models of Parkinson's disease. ACS chemical biology 1 (7):420-424. doi:10.1021/cb600331e
272. Su LJ, Auluck PK, Outeiro TF, Yeger-Lotem E, Kritzer JA, Tardiff DF, Strathearn KE, Liu F, Cao S, Hamamichi S, Hill KJ, Caldwell KA, Bell GW, Fraenkel E, Cooper AA, Caldwell GA, McCaffery JM, Rochet JC, Lindquist S (2010) Compounds from an unbiased chemical screen reverse both ER-to-Golgi trafficking defects and mitochondrial dysfunction in Parkinson's disease models. Disease models & mechanisms 3 (3-4):194-208. doi:10.1242/dmm.004267
273. Lee HJ, Kang SJ, Lee K, Im H (2011) Human alpha-synuclein modulates vesicle trafficking through its interaction with prenylated Rab acceptor protein 1. Biochemical and biophysical research communications 412 (4):526-531. doi:10.1016/j.bbrc.2011.07.028
274. Martincic I, Peralta ME, Ngsee JK (1997) Isolation and characterization of a dual prenylated Rab and VAMP2 receptor. The Journal of biological chemistry 272 (43):26991-26998
275. de Wit H, Lichtenstein Y, Kelly RB, Geuze HJ, Klumperman J, van der Sluijs P (2001) Rab4 regulates formation of synaptic-like microvesicles from early endosomes in PC12 cells. Molecular biology of the cell 12 (11):3703-3715
276. Kachhap SK, Faith D, Qian DZ, Shabbeer S, Galloway NL, Pili R, Denmeade SR, DeMarzo AM, Carducci MA (2007) The N-Myc down regulated Gene1 (NDRG1) Is a Rab4a effector involved in vesicular recycling of E-cadherin. PloS one 2 (9):e844. doi:10.1371/journal.pone.0000844
277. Kristensen AR, Gsponer J, Foster LJ (2012) A high-throughput approach for measuring temporal changes in the interactome. Nat Methods 9 (9):907-909. doi:10.1038/nmeth.2131
278. Stein S, Thomas EK, Herzog B, Westfall MD, Rocheleau JV, Jackson RS, 2nd, Wang M, Liang P (2004) NDRG1 is necessary for p53-dependent apoptosis. J Biol Chem 279 (47):48930-48940. doi:10.1074/jbc.M400386200
279. Fernandes H, Franklin E, Jollivet F, Bliedtner K, Khan AR (2012) Mapping the interactions between a RUN domain from DENND5/Rab6IP1 and sorting nexin 1. PloS one 7 (4):e35637. doi:10.1371/journal.pone.0035637
280. Mallard F, Tang BL, Galli T, Tenza D, Saint-Pol A, Yue X, Antony C, Hong W, Goud B, Johannes L (2002) Early/recycling endosomes-to-TGN transport involves two SNARE complexes and a Rab6 isoform. The Journal of cell biology 156 (4):653-664. doi:10.1083/jcb.200110081

281. Young J, Stauber T, del Nery E, Vernos I, Pepperkok R, Nilsson T (2005) Regulation of microtubule-dependent recycling at the trans-Golgi network by Rab6A and Rab6A'. Molecular biology of the cell 16 (1):162-177. doi:10.1091/mbc.E04-03-0260
282. Han SY, Park DY, Park SD, Hong SH (2000) Identification of Rab6 as an N-ethylmaleimide-sensitive fusion protein-binding protein. The Biochemical journal 352 Pt 1:165-173
283. Schwartz SL, Cao C, Pylypenko O, Rak A, Wandinger-Ness A (2007) Rab GTPases at a glance. Journal of cell science 120 (Pt 22):3905-3910. doi:10.1242/jcs.015909
284. Steuble M, Gerrits B, Ludwig A, Mateos JM, Diep TM, Tagaya M, Stephan A, Schatzle P, Kunz B, Streit P, Sonderegger P (2010) Molecular characterization of a trafficking organelle: dissecting the axonal paths of calsyntenin-1 transport vesicles. Proteomics 10 (21):3775-3788. doi:10.1002/pmic.201000384
285. Linford A, Yoshimura S, Nunes Bastos R, Langemeyer L, Gerondopoulos A, Rigden DJ, Barr FA (2012) Rab14 and its exchange factor FAM116 link endocytic recycling and adherens junction stability in migrating cells. Developmental cell 22 (5):952-966. doi:10.1016/j.devcel.2012.04.010
286. Montenegro G, Rebelo AP, Connell J, Allison R, Babalini C, D'Aloia M, Montieri P, Schule R, Ishiura H, Price J, Strickland A, Gonzalez MA, Baumbach-Reardon L, Deconinck T, Huang J, Bernardi G, Vance JM, Rogers MT, Tsuji S, De Jonghe P, Pericak-Vance MA, Schols L, Orlacchio A, Reid E, Zuchner S (2012) Mutations in the ER-shaping protein reticulon 2 cause the axon-degenerative disorder hereditary spastic paraplegia type 12. The Journal of clinical investigation 122 (2):538-544. doi:10.1172/jci60560
287. Liu Y, Vidensky S, Ruggiero AM, Maier S, Sitte HH, Rothstein JD (2008) Reticulon RTN2B regulates trafficking and function of neuronal glutamate transporter EAAC1. The Journal of biological chemistry 283 (10):6561-6571. doi:10.1074/jbc.M708096200
288. Dal Vechio FH, Cerqueira F, Augusto O, Lopes R, Demasi M (2014) Peptides that activate the 20S proteasome by gate opening increased oxidized protein removal and reduced protein aggregation. Free radical biology & medicine 67:304-313. doi:10.1016/j.freeradbiomed.2013.11.017
289. Pellom ST, Jr., Shanker A (2012) Development of Proteasome Inhibitors as Therapeutic Drugs. Journal of clinical & cellular immunology S5:5
290. Wang X, Chen CF, Baker PR, Chen PL, Kaiser P, Huang L (2007) Mass spectrometric characterization of the affinity-purified human 26S proteasome complex. Biochemistry 46 (11):3553-3565. doi:10.1021/bi061994u
291. Jayarapu K, Griffin TA (2004) Protein-protein interactions among human 20S proteasome subunits and proteassemblin. Biochemical and biophysical research communications 314 (2):523-528
292. Imai Y, Soda M, Hatakeyama S, Akagi T, Hashikawa T, Nakayama KI, Takahashi R (2002) CHIP is associated with Parkin, a gene responsible for familial Parkinson's disease, and enhances its ubiquitin ligase activity. Mol Cell 10 (1):55-67
293. Sarraf SA, Raman M, Guarani-Pereira V, Sowa ME, Huttlin EL, Gygi SP, Harper JW (2013) Landscape of the PARKIN-dependent ubiquitylome in response to mitochondrial depolarization. Nature 496 (7445):372-376. doi:10.1038/nature12043
294. Kim JS, Xu X, Li H, Solomon D, Lane WS, Jin T, Waldman T (2011) Mechanistic analysis of a DNA damage-induced, PTEN-dependent size checkpoint in human cells. Molecular and cellular biology 31 (13):2756-2771. doi:10.1128/mcb.01323-10
295. Kim E, Arnould T, Sellin L, Benzing T, Comella N, Kocher O, Tsiokas L, Sukhatme VP, Walz G (1999) Interaction between RGS7 and polycystin. Proceedings of the National Academy of Sciences of the United States of America 96 (11):6371-6376
296. Alvarez-Castelao B, Losada F, Ahicart P, Castano JG (2013) The N-terminal region of Nurr1 (a.a 1-31) is essential for its efficient degradation by the ubiquitin proteasome pathway. PloS one 8 (2):e55999. doi:10.1371/journal.pone.0055999

297. Yuen EY, Wei J, Liu W, Zhong P, Li X, Yan Z (2012) Repeated stress causes cognitive impairment by suppressing glutamate receptor expression and function in prefrontal cortex. Neuron 73 (5):962-977. doi:10.1016/j.neuron.2011.12.033
298. Hiscott J, Beauparlant P, Crepieux P, DeLuca C, Kwon H, Lin R, Petropoulos L (1997) Cellular and viral protein interactions regulating I kappa B alpha activity during human retrovirus infection. Journal of leukocyte biology 62 (1):82-92
299. Webb JL, Ravikumar B, Atkins J, Skepper JN, Rubinsztein DC (2003) Alpha-Synuclein is degraded by both autophagy and the proteasome. The Journal of biological chemistry 278 (27):25009-25013. doi:10.1074/jbc.M300227200
300. Chen E, Hrdlickova R, Nehyba J, Longo DL, Bose HR, Jr., Li CC (1998) Degradation of proto-oncoprotein c-Rel by the ubiquitin-proteasome pathway. The Journal of biological chemistry 273 (52):35201-35207
301. Tanaka T, Grusby MJ, Kaisho T (2007) PDLIM2-mediated termination of transcription factor NF-kappaB activation by intranuclear sequestration and degradation of the p65 subunit. Nature immunology 8 (6):584-591. doi:10.1038/ni1464
302. Chen HC, Jeng YM, Yuan RH, Hsu HC, Chen YL (2012) SIRT1 promotes tumorigenesis and resistance to chemotherapy in hepatocellular carcinoma and its expression predicts poor prognosis. Annals of surgical oncology 19 (6):2011-2019. doi:10.1245/s10434-011-2159-4
303. Wiper-Bergeron N, Wu D, Pope L, Schild-Poulter C, Hache RJ (2003) Stimulation of preadipocyte differentiation by steroid through targeting of an HDAC1 complex. The EMBO journal 22 (9):2135-2145. doi:10.1093/emboj/cdg218
304. Min SW, Cho SH, Zhou Y, Schroeder S, Haroutunian V, Seeley WW, Huang EJ, Shen Y, Masliah E, Mukherjee C, Meyers D, Cole PA, Ott M, Gan L (2010) Acetylation of tau inhibits its degradation and contributes to tauopathy. Neuron 67 (6):953-966. doi:10.1016/j.neuron.2010.08.044
305. Shi L, Zhang Z, Fang S, Xu J, Liu J, Shen J, Fang F, Luo L, Yin Z (2009) Heat shock protein 90 (Hsp90) regulates the stability of transforming growth factor beta-activated kinase 1 (TAK1) in interleukin-1beta-induced cell signaling. Molecular immunology 46 (4):541-550. doi:10.1016/j.molimm.2008.07.019
306. Baker SJ (2007) PTEN enters the nuclear age. Cell 128 (1):25-28. doi:10.1016/j.cell.2006.12.023
307. Dong Y, Zhang L, Zhang S, Bai Y, Chen H, Sun X, Yong W, Li W, Colvin SC, Rhodes SJ, Shou W, Zhang ZY (2012) Phosphatase of regenerating liver 2 (PRL2) is essential for placental development by down-regulating PTEN (Phosphatase and Tensin Homologue Deleted on Chromosome 10) and activating Akt protein. The Journal of biological chemistry 287 (38):32172-32179. doi:10.1074/jbc.M112.393462
308. Teske BF, Fusakio ME, Zhou D, Shan J, McClintick JN, Kilberg MS, Wek RC (2013) CHOP induces activating transcription factor 5 (ATF5) to trigger apoptosis in response to perturbations in protein homeostasis. Molecular biology of the cell 24 (15):2477-2490. doi:10.1091/mbc.E13-01-0067
309. Subramaniam SR, Chesselet MF (2013) Mitochondrial dysfunction and oxidative stress in Parkinson's disease. Progress in neurobiology 106-107:17-32. doi:10.1016/j.pneurobio.2013.04.004
310. Zhan Q, Kontny U, Iglesias M, Alamo I, Jr., Yu K, Hollander MC, Woodworth CD, Fornace AJ, Jr. (1999) Inhibitory effect of Bcl-2 on p53-mediated transactivation following genotoxic stress. Oncogene 18 (2):297-304. doi:10.1038/sj.onc.1202310
311. Panaretakis T, Pokrovskaja K, Shoshan MC, Grander D (2003) Interferon-alpha-induced apoptosis in U266 cells is associated with activation of the proapoptotic Bcl-2 family members Bak and Bax. Oncogene 22 (29):4543-4556. doi:10.1038/sj.onc.1206503
312. Bassik MC, Scorrano L, Oakes SA, Pozzan T, Korsmeyer SJ (2004) Phosphorylation of BCL-2 regulates ER Ca2+ homeostasis and apoptosis. The EMBO journal 23 (5):1207-1216. doi:10.1038/sj.emboj.7600104

313. Gross A, McDonnell JM, Korsmeyer SJ (1999) BCL-2 family members and the mitochondria in apoptosis. Genes & development 13 (15):1899-1911
314. Tsuruta F, Sunayama J, Mori Y, Hattori S, Shimizu S, Tsujimoto Y, Yoshioka K, Masuyama N, Gotoh Y (2004) JNK promotes Bax translocation to mitochondria through phosphorylation of 14-3-3 proteins. The EMBO journal 23 (8):1889-1899. doi:10.1038/sj.emboj.7600194
315. Yeh TY, Chuang JZ, Sung CH (2005) Dynein light chain rp3 acts as a nuclear matrix-associated transcriptional modulator in a dynein-independent pathway. Journal of cell science 118 (Pt 15):3431-3443. doi:10.1242/jcs.02472
316. Feng Z, Porter AG (1999) NF-kappaB/Rel proteins are required for neuronal differentiation of SH-SY5Y neuroblastoma cells. The Journal of biological chemistry 274 (43):30341-30344
317. Hess P, Pihan G, Sawyers CL, Flavell RA, Davis RJ (2002) Survival signaling mediated by c-Jun NH(2)-terminal kinase in transformed B lymphoblasts. Nature genetics 32 (1):201-205. doi:10.1038/ng946
318. Yamamoto K, Ichijo H, Korsmeyer SJ (1999) BCL-2 is phosphorylated and inactivated by an ASK1/Jun N-terminal protein kinase pathway normally activated at G(2)/M. Molecular and cellular biology 19 (12):8469-8478
319. Park J, Kim I, Oh YJ, Lee K, Han PL, Choi EJ (1997) Activation of c-Jun N-terminal kinase antagonizes an anti-apoptotic action of Bcl-2. The Journal of biological chemistry 272 (27):16725-16728
320. de Moissac D, Mustapha S, Greenberg AH, Kirshenbaum LA (1998) Bcl-2 activates the transcription factor NFkappaB through the degradation of the cytoplasmic inhibitor IkappaBalpha. The Journal of biological chemistry 273 (37):23946-23951
321. Liang Y, Mirnics ZK, Yan C, Nylander KD, Schor NF (2003) Bcl-2 mediates induction of neural differentiation. Oncogene 22 (35):5515-5518. doi:10.1038/sj.onc.1206844
322. Huang H, Cheville JC, Pan Y, Roche PC, Schmidt LJ, Tindall DJ (2001) PTEN induces chemosensitivity in PTEN-mutated prostate cancer cells by suppression of Bcl-2 expression. The Journal of biological chemistry 276 (42):38830-38836. doi:10.1074/jbc.M103632200
323. Kamachi M, Le TM, Kim SJ, Geiger ME, Anderson P, Utz PJ (2002) Human autoimmune sera as molecular probes for the identification of an autoantigen kinase signaling pathway. J Exp Med 196 (9):1213-1225
324. Kang CB, Tai J, Chia J, Yoon HS (2005) The flexible loop of Bcl-2 is required for molecular interaction with immunosuppressant FK-506 binding protein 38 (FKBP38). FEBS letters 579 (6):1469-1476. doi:10.1016/j.febslet.2005.01.053
325. Liang Y, Nylander KD, Yan C, Schor NF (2002) Role of caspase 3-dependent Bcl-2 cleavage in potentiation of apoptosis by Bcl-2. Molecular pharmacology 61 (1):142-149
326. Chen D, Gao F, Li B, Wang H, Xu Y, Zhu C, Wang G (2010) Parkin mono-ubiquitinates Bcl-2 and regulates autophagy. The Journal of biological chemistry 285 (49):38214-38223. doi:10.1074/jbc.M110.101469
327. Swanton E, Savory P, Cosulich S, Clarke P, Woodman P (1999) Bcl-2 regulates a caspase-3/caspase-2 apoptotic cascade in cytosolic extracts. Oncogene 18 (10):1781-1787. doi:10.1038/sj.onc.1202490
328. Kuwana T, Newmeyer DD (2003) Bcl-2-family proteins and the role of mitochondria in apoptosis. Current opinion in cell biology 15 (6):691-699
329. Sutton VR, Wowk ME, Cancilla M, Trapani JA (2003) Caspase activation by granzyme B is indirect, and caspase autoprocessing requires the release of proapoptotic mitochondrial factors. Immunity 18 (3):319-329
330. Vande Walle L, Van Damme P, Lamkanfi M, Saelens X, Vandekerckhove J, Gevaert K, Vandenabeele P (2007) Proteome-wide Identification of HtrA2/Omi Substrates. J Proteome Res 6 (3):1006-1015. doi:10.1021/pr060510d
331. Park HM, Kim GY, Nam MK, Seong GH, Han C, Chung KC, Kang S, Rhim H (2009) The serine protease HtrA2/Omi cleaves Parkin and irreversibly inactivates its E3 ubiquitin ligase activity. Biochem Biophys Res Commun 387 (3):537-542. doi:10.1016/j.bbrc.2009.07.079

332. Plun-Favreau H, Klupsch K, Moisoi N, Gandhi S, Kjaer S, Frith D, Harvey K, Deas E, Harvey RJ, McDonald N, Wood NW, Martins LM, Downward J (2007) The mitochondrial protease HtrA2 is regulated by Parkinson's disease-associated kinase PINK1. Nat Cell Biol 9 (11):1243-1252. doi:10.1038/ncb1644
333. Kawahara K, Hashimoto M, Bar-On P, Ho GJ, Crews L, Mizuno H, Rockenstein E, Imam SZ, Masliah E (2008) alpha-Synuclein aggregates interfere with Parkin solubility and distribution: role in the pathogenesis of Parkinson disease. J Biol Chem 283 (11):6979-6987. doi:10.1074/jbc.M710418200
334. Masliah E, Rockenstein E, Veinbergs I, Mallory M, Hashimoto M, Takeda A, Sagara Y, Sisk A, Mucke L (2000) Dopaminergic loss and inclusion body formation in alpha-synuclein mice: implications for neurodegenerative disorders. Science 287 (5456):1265-1269
335. Klein P, Muller-Rischart AK, Motori E, Schonbauer C, Schnorrer F, Winklhofer KF, Klein R (2014) Ret rescues mitochondrial morphology and muscle degeneration of Drosophila Pink1 mutants. The EMBO journal 33 (4):341-355. doi:10.1002/embj.201284290
336. McGuire V, Van Den Eeden SK, Tanner CM, Kamel F, Umbach DM, Marder K, Mayeux R, Ritz B, Ross GW, Petrovitch H, Topol B, Popat RA, Costello S, Manthripragada AD, Southwick A, Myers RM, Nelson LM (2011) Association of DRD2 and DRD3 polymorphisms with Parkinson's disease in a multiethnic consortium. J Neurol Sci 307 (1-2):22-29. doi:10.1016/j.jns.2011.05.031
337. Grevle L, Guzey C, Hadidi H, Brennersted R, Idle JR, Aasly J (2000) Allelic association between the DRD2 TaqI A polymorphism and Parkinson's disease. Mov Disord 15 (6):1070-1074
338. Hayes G, Biden TJ, Selbie LA, Shine J (1992) Structural subtypes of the dopamine D2 receptor are functionally distinct: expression of the cloned D2A and D2B subtypes in a heterologous cell line. Molecular endocrinology (Baltimore, Md) 6 (6):920-926. doi:10.1210/mend.6.6.1323056
339. Hattori K, Uchino S, Isosaka T, Maekawa M, Iyo M, Sato T, Kohsaka S, Yagi T, Yuasa S (2006) Fyn is required for haloperidol-induced catalepsy in mice. J Biol Chem 281 (11):7129-7135. doi:10.1074/jbc.M511608200
340. Page KM, Canti C, Stephens GJ, Berrow NS, Dolphin AC (1998) Identification of the amino terminus of neuronal Ca2+ channel alpha1 subunits alpha1B and alpha1E as an essential determinant of G-protein modulation. J Neurosci 18 (13):4815-4824
341. Kuzhikandathil EV, Oxford GS (1999) Activation of human D3 dopamine receptor inhibits P/Q-type calcium channels and secretory activity in AtT-20 cells. J Neurosci 19 (5):1698-1707
342. Hussain NK, Jenna S, Glogauer M, Quinn CC, Wasiak S, Guipponi M, Antonarakis SE, Kay BK, Stossel TP, Lamarche-Vane N, McPherson PS (2001) Endocytic protein intersectin-l regulates actin assembly via Cdc42 and N-WASP. Nat Cell Biol 3 (10):927-932. doi:10.1038/ncb1001-927
343. Shen G, Whittington A, Wang P (2011) Wsp1, a GBD/CRIB domain-containing WASP homolog, is required for growth, morphogenesis, and virulence of Cryptococcus neoformans. Eukaryot Cell 10 (4):521-529. doi:10.1128/ec.00274-10
344. Fu X, Brown KJ, Yap CC, Winckler B, Jaiswal JK, Liu JS (2013) Doublecortin (Dcx) family proteins regulate filamentous actin structure in developing neurons. The Journal of neuroscience : the official journal of the Society for Neuroscience 33 (2):709-721. doi:10.1523/jneurosci.4603-12.2013
345. Liang XQ, Avraham HK, Jiang S, Avraham S (2004) Genetic alterations of the NRP/B gene are associated with human brain tumors. Oncogene 23 (35):5890-5900. doi:10.1038/sj.onc.1207776
346. Park SK, Nguyen MD, Fischer A, Luke MP, Affar el B, Dieffenbach PB, Tseng HC, Shi Y, Tsai LH (2005) Par-4 links dopamine signaling and depression. Cell 122 (2):275-287. doi:10.1016/j.cell.2005.05.031
347. Navarro G, Aymerich MS, Marcellino D, Cortes A, Casado V, Mallol J, Canela EI, Agnati L, Woods AS, Fuxe K, Lluis C, Lanciego JL, Ferre S, Franco R (2009) Interactions between calmodulin,

adenosine A2A, and dopamine D2 receptors. J Biol Chem 284 (41):28058-28068. doi:10.1074/jbc.M109.034231
348. Leonard AS, Bayer KU, Merrill MA, Lim IA, Shea MA, Schulman H, Hell JW (2002) Regulation of calcium/calmodulin-dependent protein kinase II docking to N-methyl-D-aspartate receptors by calcium/calmodulin and alpha-actinin. J Biol Chem 277 (50):48441-48448. doi:10.1074/jbc.M205164200
349. Martinez J, Moeller I, Erdjument-Bromage H, Tempst P, Lauring B (2003) Parkinson's disease-associated alpha-synuclein is a calmodulin substrate. The Journal of biological chemistry 278 (19):17379-17387. doi:10.1074/jbc.M209020200
350. Zainelli GM, Ross CA, Troncoso JC, Fitzgerald JK, Muma NA (2004) Calmodulin regulates transglutaminase 2 cross-linking of huntingtin. The Journal of neuroscience : the official journal of the Society for Neuroscience 24 (8):1954-1961. doi:10.1523/jneurosci.4424-03.2004
351. Yus-Najera E, Santana-Castro I, Villarroel A (2002) The identification and characterization of a noncontinuous calmodulin-binding site in noninactivating voltage-dependent KCNQ potassium channels. J Biol Chem 277 (32):28545-28553. doi:10.1074/jbc.M204130200
352. Berggard T, Arrigoni G, Olsson O, Fex M, Linse S, James P (2006) 140 mouse brain proteins identified by Ca2+-calmodulin affinity chromatography and tandem mass spectrometry. Journal of proteome research 5 (3):669-687. doi:10.1021/pr050421l
353. Bal M, Zhang J, Hernandez CC, Zaika O, Shapiro MS (2010) Ca2+/calmodulin disrupts AKAP79/150 interactions with KCNQ (M-Type) K+ channels. The Journal of neuroscience : the official journal of the Society for Neuroscience 30 (6):2311-2323. doi:10.1523/jneurosci.5175-09.2010
354. Krupp JJ, Vissel B, Thomas CG, Heinemann SF, Westbrook GL (1999) Interactions of calmodulin and alpha-actinin with the NR1 subunit modulate Ca2+-dependent inactivation of NMDA receptors. J Neurosci 19 (4):1165-1178
355. Lu WY, Jackson MF, Bai D, Orser BA, MacDonald JF (2000) In CA1 pyramidal neurons of the hippocampus protein kinase C regulates calcium-dependent inactivation of NMDA receptors. J Neurosci 20 (12):4452-4461
356. Herdegen T, Leah JD (1998) Inducible and constitutive transcription factors in the mammalian nervous system: control of gene expression by Jun, Fos and Krox, and CREB/ATF proteins. Brain Res Brain Res Rev 28 (3):370-490
357. Muthusamy N, Leiden JM (1998) A protein kinase C-, Ras-, and RSK2-dependent signal transduction pathway activates the cAMP-responsive element-binding protein transcription factor following T cell receptor engagement. J Biol Chem 273 (35):22841-22847
358. Takemoto-Kimura S, Terai H, Takamoto M, Ohmae S, Kikumura S, Segi E, Arakawa Y, Furuyashiki T, Narumiya S, Bito H (2003) Molecular cloning and characterization of CLICK-III/CaMKIgamma, a novel membrane-anchored neuronal Ca2+/calmodulin-dependent protein kinase (CaMK). J Biol Chem 278 (20):18597-18605. doi:10.1074/jbc.M300578200
359. Wang Z, Collighan RJ, Pytel K, Rathbone DL, Li X, Griffin M (2012) Characterization of heparin-binding site of tissue transglutaminase: its importance in cell surface targeting, matrix deposition, and cell signaling. The Journal of biological chemistry 287 (16):13063-13083. doi:10.1074/jbc.M111.294819
360. Satpathy M, Shao M, Emerson R, Donner DB, Matei D (2009) Tissue transglutaminase regulates matrix metalloproteinase-2 in ovarian cancer by modulating cAMP-response element-binding protein activity. The Journal of biological chemistry 284 (23):15390-15399. doi:10.1074/jbc.M808331200
361. Yamaguchi H, Wang HG (2006) Tissue transglutaminase serves as an inhibitor of apoptosis by cross-linking caspase 3 in thapsigargin-treated cells. Molecular and cellular biology 26 (2):569-579. doi:10.1128/mcb.26.2.569-579.2006

362. Csomos K, Nemet I, Fesus L, Balajthy Z (2010) Tissue transglutaminase contributes to the all-trans-retinoic acid-induced differentiation syndrome phenotype in the NB4 model of acute promyelocytic leukemia. Blood 116 (19):3933-3943. doi:10.1182/blood-2010-01-266064
363. Lesort M, Chun W, Tucholski J, Johnson GV (2002) Does tissue transglutaminase play a role in Huntington's disease? Neurochemistry international 40 (1):37-52
364. Johnson K, Hashimoto S, Lotz M, Pritzker K, Terkeltaub R (2001) Interleukin-1 induces pro-mineralizing activity of cartilage tissue transglutaminase and factor XIIIa. The American journal of pathology 159 (1):149-163. doi:10.1016/s0002-9440(10)61682-3
365. Junn E, Ronchetti RD, Quezado MM, Kim SY, Mouradian MM (2003) Tissue transglutaminase-induced aggregation of alpha-synuclein: Implications for Lewy body formation in Parkinson's disease and dementia with Lewy bodies. Proceedings of the National Academy of Sciences of the United States of America 100 (4):2047-2052. doi:10.1073/pnas.0438021100
366. Bailey CD, Tucholski J, Johnson GV (2005) Transglutaminases in neurodegenerative disorders. Progress in experimental tumor research 38:139-157. doi:10.1159/000084238
367. Kim SJ, Kim MY, Lee EJ, Ahn YS, Baik JH (2004) Distinct regulation of internalization and mitogen-activated protein kinase activation by two isoforms of the dopamine D2 receptor. Mol Endocrinol 18 (3):640-652. doi:10.1210/me.2003-0066
368. Yan Z, Feng J, Fienberg AA, Greengard P (1999) D(2) dopamine receptors induce mitogen-activated protein kinase and cAMP response element-binding protein phosphorylation in neurons. Proc Natl Acad Sci U S A 96 (20):11607-11612
369. von Kriegsheim A, Baiocchi D, Birtwistle M, Sumpton D, Bienvenut W, Morrice N, Yamada K, Lamond A, Kalna G, Orton R, Gilbert D, Kolch W (2009) Cell fate decisions are specified by the dynamic ERK interactome. Nat Cell Biol 11 (12):1458-1464. doi:10.1038/ncb1994
370. Iwata A, Maruyama M, Kanazawa I, Nukina N (2001) alpha-Synuclein affects the MAPK pathway and accelerates cell death. J Biol Chem 276 (48):45320-45329. doi:10.1074/jbc.M103736200
371. Wang Z, Liu J, Chen S, Wang Y, Cao L, Zhang Y, Kang W, Li H, Gui Y, Chen S, Ding J (2011) DJ-1 modulates the expression of Cu/Zn-superoxide dismutase-1 through the Erk1/2-Elk1 pathway in neuroprotection. Annals of neurology 70 (4):591-599. doi:10.1002/ana.22514
372. Song C, Zhou X, Dong Q, Fan R, Wu G, Ji B, Meng Q, Zheng M (2012) Regulation of inflammatory response in human chondrocytes by lentiviral mediated RNA interference against S100A10. Inflammation research : official journal of the European Histamine Research Society [et al] 61 (11):1219-1227. doi:10.1007/s00011-012-0519-6
373. Predescu SA, Predescu DN, Knezevic I, Klein IK, Malik AB (2007) Intersectin-1s regulates the mitochondrial apoptotic pathway in endothelial cells. J Biol Chem 282 (23):17166-17178. doi:10.1074/jbc.M608996200
374. Ryu SH, Kim KH, Kim HB, Kim MH, Kim NH, Kang Y, Hyun JW, Seo HJ, Jun JY, You HJ (2010) Oncogenic Ras-mediated downregulation of Clast1/LR8 is involved in Ras-mediated neoplastic transformation and tumorigenesis in NIH3T3 cells. Cancer science 101 (9):1990-1996. doi:10.1111/j.1349-7006.2010.01626.x
375. Roberson ED, English JD, Adams JP, Selcher JC, Kondratick C, Sweatt JD (1999) The mitogen-activated protein kinase cascade couples PKA and PKC to cAMP response element binding protein phosphorylation in area CA1 of hippocampus. J Neurosci 19 (11):4337-4348
376. He Z, Jiang J, Kokkinaki M, Golestaneh N, Hofmann MC, Dym M (2008) Gdnf upregulates c-Fos transcription via the Ras/Erk1/2 pathway to promote mouse spermatogonial stem cell proliferation. Stem Cells 26 (1):266-278. doi:10.1634/stemcells.2007-0436
377. Almeida LE, Murray PD, Zielke HR, Roby CD, Kingsbury TJ, Krueger BK (2009) Autocrine activation of neuronal NMDA receptors by aspartate mediates dopamine- and cAMP-induced CREB-dependent gene transcription. The Journal of neuroscience : the official journal of the Society for Neuroscience 29 (40):12702-12710. doi:10.1523/jneurosci.1166-09.2009
378. Pende M, Fisher TL, Simpson PB, Russell JT, Blenis J, Gallo V (1997) Neurotransmitter- and growth factor-induced cAMP response element binding protein phosphorylation in glial cell progenitors: role of calcium ions, protein kinase C, and mitogen-activated protein

kinase/ribosomal S6 kinase pathway. The Journal of neuroscience : the official journal of the Society for Neuroscience 17 (4):1291-1301
379. Tao X, Finkbeiner S, Arnold DB, Shaywitz AJ, Greenberg ME (1998) Ca2+ influx regulates BDNF transcription by a CREB family transcription factor-dependent mechanism. Neuron 20 (4):709-726
380. Cole DG, Kobierski LA, Konradi C, Hyman SE (1994) 6-Hydroxydopamine lesions of rat substantia nigra up-regulate dopamine-induced phosphorylation of the cAMP-response element-binding protein in striatal neurons. Proc Natl Acad Sci U S A 91 (20):9631-9635
381. Westin JE, Vercammen L, Strome EM, Konradi C, Cenci MA (2007) Spatiotemporal pattern of striatal ERK1/2 phosphorylation in a rat model of L-DOPA-induced dyskinesia and the role of dopamine D1 receptors. Biological psychiatry 62 (7):800-810. doi:10.1016/j.biopsych.2006.11.032
382. Andersson M, Konradi C, Cenci MA (2001) cAMP response element-binding protein is required for dopamine-dependent gene expression in the intact but not the dopamine-denervated striatum. The Journal of neuroscience : the official journal of the Society for Neuroscience 21 (24):9930-9943
383. Wadle A, Thiel G, Mischo A, Jung V, Pfreundschuh M, Renner C (2001) Chromosomal localization and promoter analysis of the adenomatous polyposis coli binding protein RP1. Oncogene 20 (41):5920-5929. doi:10.1038/sj.onc.1204797
384. Lang T, Wacker I, Steyer J, Kaether C, Wunderlich I, Soldati T, Gerdes HH, Almers W (1997) Ca2+-triggered peptide secretion in single cells imaged with green fluorescent protein and evanescent-wave microscopy. Neuron 18 (6):857-863
385. Law V, Knox C, Djoumbou Y, Jewison T, Guo AC, Liu Y, Maciejewski A, Arndt D, Wilson M, Neveu V, Tang A, Gabriel G, Ly C, Adamjee S, Dame ZT, Han B, Zhou Y, Wishart DS (2014) DrugBank 4.0: shedding new light on drug metabolism. Nucleic acids research 42 (Database issue):D1091-1097. doi:10.1093/nar/gkt1068
386. Quintana A, Sgambato-Faure V, Savasta M (2012) Effects of L-DOPA and STN-HFS dyskinesiogenic treatments on NR2B regulation in basal ganglia in the rat model of Parkinson's disease. Neurobiology of disease 48 (3):379-390. doi:10.1016/j.nbd.2012.06.009
387. Bordet R, Ridray S, Carboni S, Diaz J, Sokoloff P, Schwartz JC (1997) Induction of dopamine D3 receptor expression as a mechanism of behavioral sensitization to levodopa. Proceedings of the National Academy of Sciences of the United States of America 94 (7):3363-3367
388. Liu WG, Chen Y, Li B, Lu GQ, Chen SD (2004) Neuroprotection by pergolide against levodopa-induced cytotoxicity of neural stem cells. Neurochem Res 29 (12):2207-2214
389. Zhang X, Andren PE, Greengard P, Svenningsson P (2008) Evidence for a role of the 5-HT1B receptor and its adaptor protein, p11, in L-DOPA treatment of an animal model of Parkinsonism. Proceedings of the National Academy of Sciences of the United States of America 105 (6):2163-2168. doi:10.1073/pnas.0711839105
390. Pavon N, Martin AB, Mendialdua A, Moratalla R (2006) ERK phosphorylation and FosB expression are associated with L-DOPA-induced dyskinesia in hemiparkinsonian mice. Biological psychiatry 59 (1):64-74. doi:10.1016/j.biopsych.2005.05.044
391. Santini E, Valjent E, Usiello A, Carta M, Borgkvist A, Girault JA, Herve D, Greengard P, Fisone G (2007) Critical involvement of cAMP/DARPP-32 and extracellular signal-regulated protein kinase signaling in L-DOPA-induced dyskinesia. The Journal of neuroscience : the official journal of the Society for Neuroscience 27 (26):6995-7005. doi:10.1523/jneurosci.0852-07.2007
392. Beaumont TL, Yao B, Shah A, Kapatos G, Loeb JA (2012) Layer-specific CREB target gene induction in human neocortical epilepsy. J Neurosci 32 (41):14389-14401. doi:10.1523/jneurosci.3408-12.2012
393. Zhang L, Gao J, Li L, Li Z, Du Y, Gong Y (2011) The neuronal pentraxin II gene (NPTX2) inhibit proliferation and invasion of pancreatic cancer cells in vitro. Mol Biol Rep 38 (8):4903-4911. doi:10.1007/s11033-010-0632-y

394. Yus-Najera E, Munoz A, Salvador N, Jensen BS, Rasmussen HB, Defelipe J, Villarroel A (2003) Localization of KCNQ5 in the normal and epileptic human temporal neocortex and hippocampal formation. Neuroscience 120 (2):353-364
395. Clasadonte J, Dong J, Hines DJ, Haydon PG (2013) Astrocyte control of synaptic NMDA receptors contributes to the progressive development of temporal lobe epilepsy. Proceedings of the National Academy of Sciences of the United States of America 110 (43):17540-17545. doi:10.1073/pnas.1311967110
396. Andrade DM (2009) Genetic basis in epilepsies caused by malformations of cortical development and in those with structurally normal brain. Human genetics 126 (1):173-193. doi:10.1007/s00439-009-0702-1

LIST OF ABBREVIATIONS

Abbreviation	Protein name
ACHE	acetylcholinesterase (Yt blood group)
ACOT7	acyl-CoA thioesterase 7
ACTG1	actin, gamma 1
AKT1	v-akt murine thymoma viral oncogene homolog 1
AKT1S1	AKT1 substrate 1 (proline-rich)
ANP32B	acidic (leucine-rich) nuclear phosphoprotein 32 family, member B
ATP5C1	ATP synthase, H+ transporting, mitochondrial F1 complex, gamma polypeptide 1
ATP6V0D1	ATPase, H+ transporting, lysosomal 38kDa, V0 subunit d1
ATXN10	ataxin 10
BAX	BCL2-associated X protein
BCL2	B-cell CLL/lymphoma 2
CaM	calmodulin
CAMK1G	calcium/calmodulin-dependent protein kinase IG
CASC3	cancer susceptibility candidate 3
CASP3	caspase 3, apoptosis-related cysteine peptidase
CCDC6	coiled-coil domain containing 6
CDH2	cadherin 2, type 1, N-cadherin (neuronal)
CDH8	cadherin 8, type 2
CDK5	cyclin-dependent kinase 5
CHGB	chromogranin B (secretogranin 1)
CHP1	calcineurin-like EF-hand protein 1
CLK4	CDC-like kinase 4
CREB1	cAMP responsive element binding protein 1
CRMP1	collapsin response mediator protein 1
CTBP2	C-terminal binding protein 2
DCLK1	doublecortin-like kinase 1
DDX1	DEAD (Asp-Glu-Ala-Asp) box helicase 1
DDX5	DEAD (Asp-Glu-Ala-Asp) box helicase 5
DHX36	DEAH (Asp-Glu-Ala-His) box polypeptide 36
DIRAS2	DIRAS family, GTP-binding RAS-like 2
DLK1	delta-like 1 homolog (Drosophila)
DRD2	dopamine receptor D2
DRD3	dopamine receptor D3
DUSP19	dual specificity phosphatase 19
EIF1B	eukaryotic translation initiation factor 1B
EIF4G1	eukaryotic translation initiation factor 4 gamma, 1
ENC1	ectodermal-neural cortex 1 (with BTB domain)
ERK1/2	extracellular-signal-regulated kinase 1/2
FOXO1	forkhead box O1
FRAT2	frequently rearranged in advanced T-cell lymphomas 2
FYN	FYN proto-oncogene, Src family tyrosine kinase
GABRG2	gamma-aminobutyric acid (GABA) A receptor, gamma 2
GIGYF2	GRB10 interacting GYF protein 2
GNAI2	guanine nucleotide binding protein (G protein), alpha inhibiting activity polypeptide 2

GRIN1	glutamate receptor, ionotropic, N-methyl D-aspartate 1
GTF2I	general transcription factor IIi
H2AFJ	H2A histone family, member J
HDAC1	histone deacetylase 1
HNRNPH3	heterogeneous nuclear ribonucleoprotein H3 (2H9)
HTRA2	HtrA serine peptidase 2
ITSN1	intersectin 1 (SH3 domain protein)
KCNQ5	potassium voltage-gated channel, KQT-like subfamily, member 5
KIFAP3	kinesin-associated protein 3
KLC1	kinesin light chain 1
L1CAM	L1 cell adhesion molecule
LPAR1	lysophosphatidic acid receptor 1
LRRK2	leucine-rich repeat kinase 2
MAGED1	melanoma antigen family D, 1
MAGOH1	Protein mago nashi homolog 1
MAP2K4	mitogen-activated protein kinase kinase 4
MAP3K7	mitogen-activated protein kinase kinase kinase 7
MAP4	microtubule-associated protein 4
MAPK8	mitogen-activated protein kinase 8
MAPRE2	microtubule-associated protein, RP/EB family, member 2
MAPT	microtubule-associated protein tau
MARK2	MAP/microtubule affinity-regulating kinase 2
MRPL15	mitochondrial ribosomal protein L15
mTORC1	mammalian target of rapamycin complex 1
MXD4	MAX dimerization protein 4
NAPB	N-ethylmaleimide-sensitive factor attachment protein, beta
NDRG1	N-myc downstream regulated 1
NFIB	nuclear factor I/B
NFKB	nuclear factor kappa-light-chain-enhancer of activated B cells
NFKBIA	nuclear factor of kappa light polypeptide gene enhancer in B-cells inhibitor, alpha
NMDAR	N-methyl-D-aspartate receptor
NPTX2	neuronal pentraxin II
NR4A2	nuclear receptor subfamily 4, group A, member 2
NSF	N-ethylmaleimide-sensitive factor
PAPOLA	poly(A) polymerase alpha
PARK2	parkin RBR E3 ubiquitin protein ligase
PARK7	parkinson protein 7
PDXK	pyridoxal (pyridoxine, vitamin B6) kinase
PFKM	phosphofructokinase, muscle
PINK1	PTEN induced putative kinase 1
PITX3	paired-like homeodomain 3
PPP2R2A	protein phosphatase 2, regulatory subunit B, alpha
PSMA1	proteasome (prosome, macropain) subunit, alpha type, 1
PSMB5	proteasome (prosome, macropain) subunit, beta type, 5
PTEN	phosphatase and tensin homolog
RAB11A	RAB11A, member RAS oncogene family
RAB14	RAB14, member RAS oncogene family
RAB4A	RAB4A, member RAS oncogene family
RAB6A	RAB6A, member RAS oncogene family
RABAC1	Rab acceptor 1 (prenylated)

RAC1	ras-related C3 botulinum toxin substrate 1 (rho family, small GTP binding protein Rac1)
RANBP9	RAN binding protein 9
RAP1GDS1	RAP1, GTP-GDP dissociation stimulator 1
RBM39	RNA binding motif protein 39
RELA	v-rel avian reticuloendotheliosis viral oncogene homolog A
RET	ret proto-oncogene
RGS4	regulator of G-protein signaling 4
RGS7	regulator of G-protein signaling 7
RPL10A	ribosomal protein L10a
RPL5	ribosomal protein L5
RTN2	reticulon 2
S100A10	S100 calcium binding protein A10
SASH1	SAM and SH3 domain containing 1
SATB1	SATB homeobox 1
SIRT1	sirtuin 1
SLC18A2	solute carrier family 18 (vesicular monoamine transporter), member 2
SLC4A3	solute carrier family 4 (anion exchanger), member 3
SLC4A8	solute carrier family 4, sodium bicarbonate cotransporter, member 8
SLC6A3	solute carrier family 6 (neurotransmitter transporter), member 3
SLC8A1	solute carrier family 8 (sodium/calcium exchanger), member 1
SNAP25	synaptosomal-associated protein, 25kDa
SNCA	synuclein, alpha (non A4 component of amyloid precursor)
SOX2	SRY (sex determining region Y)-box 2
SRPK2	SRSF protein kinase 2
SRSF7	serine/arginine-rich splicing factor 7
STXBP1	syntaxin binding protein 1
TGM2	transglutaminase 2
TH	tyrosine hydroxylase
TMEM176B	transmembrane protein 176B
TNK2	tyrosine kinase, non-receptor, 2
TSPAN7	tetraspanin 7
UCHL1	ubiquitin carboxyl-terminal esterase L1 (ubiquitin thiolesterase)
USP22	ubiquitin specific peptidase 22
VAMP2	vesicle-associated membrane protein 2 (synaptobrevin 2)
VDCC	voltage-dependent calcium channel
VSNL1	visinin-like 1
YTHDF2	YTH domain family, member 2
YWHAZ	tyrosine 3-monooxygenase/tryptophan 5-monooxygenase activation protein, zeta
ZC3H11A	zinc finger CCCH-type containing 11A
